# Frontier Advances of Terpyridine–Zn(II) Complexes: From Molecular Design to Smart Functional Materials

**DOI:** 10.1002/advs.76295

**Published:** 2026-06-29

**Authors:** Lixin Duan, Qingshu Zheng, Yifan Fan, Caiyun Fang, Tao Tu

**Affiliations:** ^1^ State Key Laboratory of High–efficiency Utilization of Coal and Green Chemical Engineering School of Chemistry and Chemical Engineering Ningxia University Yinchuan China; ^2^ State Key Laboratory of Green Chemical Synthesis and Conversion Department of Chemistry Fudan University Shanghai China; ^3^ Zhejiang Key Laboratory of Extreme Environment Functional Materials Yiwu Research Institute of Fudan University Yiwu China; ^4^ State Key Laboratory of Organometallic Chemistry, Shanghai Institute of Organic Chemistry Chinese Academy of Sciences Shanghai China

**Keywords:** functional materials, photocatalysis, supramolecular assembly, Terpyridine–Zn complex, visual recognition

## Abstract

As one of the most important pincer coordination molecules, terpyridine (Tpy) metal complexes have emerged as a vibrant research frontier in coordination chemistry, catalysis and materials science due to their unique chelation versatility and modular structural tunability. In particular, Tpy–Zn complexes exhibit exceptional photochemical and photophysical characteristics, low toxicity, and self‐assembly propensity, making them a novel and versatile platform for functional material design. This review comprehensively summarizes the design strategies of various Tpy–Zn complexes and their corresponding materials, including discrete molecular complexes, supramolecular assemblies (e.g., gels, metal organic frameworks, cages) and their composites, while highlighting their recent advances in three key domains: (1) visual molecular recognition and sensing, (2) stimuli‐responsive smart functional materials, and (3) photocatalytic organic transformations. Additionally, remaining synthetic obstacles, unresolved controversies in structure–property relationships, and future perspectives for the development of novel Tpy–Zn complexes are thoroughly discussed. This review aims to establish a rational design paradigm for next–generation Tpy–Zn–based functional materials, thereby providing in‐depth mechanistic insights and sparking their innovative applications functional materials science.

## Graphical abstract

Tpy–Zn complexes serve as versatile building blocks for the modular assembly of functional materials, including polymers, gels, MOFs, cages, and composites through programmed noncovalent interactions, empowering practical applications in visual molecular recognition, smart functional materials, and photocatalytic transformations.

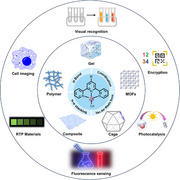

## 1 Introduction

**FIGURE 1:**
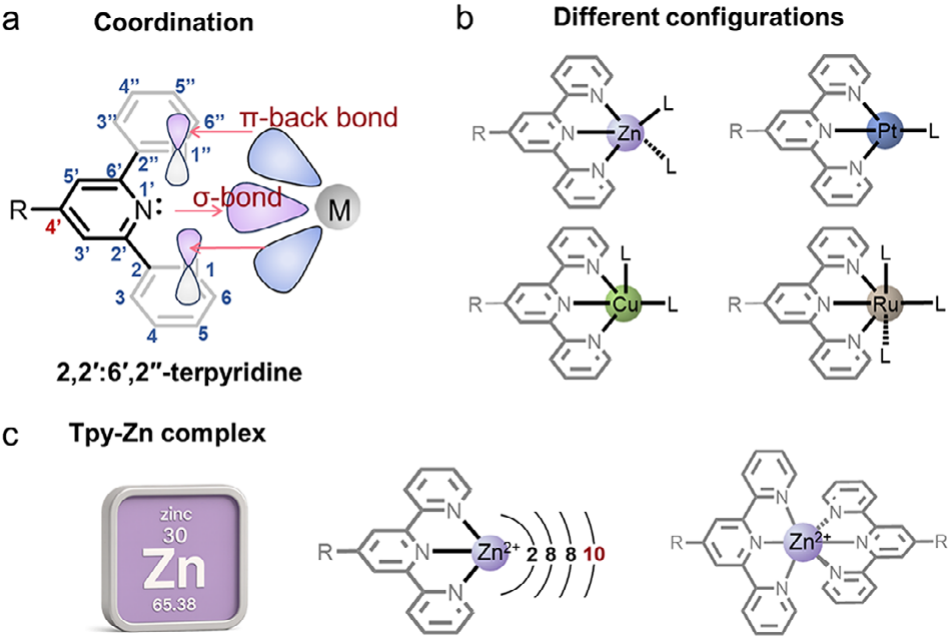
(a) Schematic representation of σ–bond and π–back bonds formation during coordination of 2,2′:6′,2″–Tpy with various metal ions. (b) Possible coordination geometry of Tpy ligands with selected metal ions. (c) The properties and characteristics of Zn atom and related Tpy compounds.

Functional materials derived from low molecular–weight molecules play essential roles in molecular recognition [1, 2, 3, 4], gas separation [5, 6, 7, 8], catalysis [9, 10, 11], sensing [12, 13, 14, 15], data storage [16, 17, 18], and smart devices [19, 20, 21]. Among these, metal coordination complexes have garnered extensive research interest for their well–defined structures, tunable coordination geometry, stimuli–responsive properties, metal‐to‐ligand charge transfer (MLCT) characteristics, as well as distinct photophysical and photochemical features [22, 23, 24]. In particular, 2,2′:6′,2″–terpyridine (Tpy) has become a foundational building block in this research field, owing to its strong σ–donating ability, high affinity to various metal ions, and extended π–conjugated system (Figure 1a) [25, 26, 27]. The modularity of Tpy allows for facile functionalization at the 4′–position or on the peripheral aromatic rings, enabling precise control over electronic properties, solubility, and self–assembly behavior [28, 29, 30]. Coordination with various metal (M) ions further imparts corresponding complexes with predictable geometries, rich photophysical features, and structural adaptability, thereby offering a robust platform for the fabrication of smart functional materials or composites (Figure 1b) [31, 32]. Compared with bidentate pyridyl ligands such as 2,2’–bipyridine (bpy) and 1,10–phenanthroline (phen), the Tpy ligand possesses several distinct advantages, including ease of synthesis and functionalization. Due to its tridentate chelating ability, Tpy enables the formation of well–defined octahedral [Tpy–M–Tpy]^n+^ complexes, which exhibit robust yet dynamic coordination stability, leading to fewer structural isomers compared to their bidentate counterparts. Moreover, its highly extended π–conjugated planar metal–hybridized aromatic rings endow the corresponding complexes with excellent and tunable photophysical and redox properties. Notably, Tpy can be readily functionalized at various positions, allowing systematic modulation of its solubility, assembly, and stimulus–responsive properties, thereby significantly expanding its applicability in catalysis, materials, and devices [33, 34, 35].

The synthesis, characterization, and applications of Tpy–M complexes have been extensively investigated over the past decades, especially with noble metal ions. Seminal works by Prof. Che and Prof. Yam have achieved remarkable progress in the development of complexes containing Au(I or III) [36, 37, 38], Pd(II) [39, 40], and Pt(II) [41, 42, 43, 44, 45], etc. Their works provided deep insights into the photophysical and photochemical behavior of these complexes, elucidating plausible structure–property relationships that underpin their performance in luminescent devices, sensors, and photocatalysis. In contrast, Tpy–M complexes containing earth–abundant transition metal ions are less studied, although they are inexpensive, readily available, robust under ambient conditions, and exhibit high biocompatibility [25, 46, 47]. Among them, Zn(II) ions have drawn significant consideration as candidates for constructing robust Tpy–M complexes. As a d^10^ closed shell ion, Zn(II) combines low toxicity and biocompatibility with unique coordination features (Figure 1c). Its fully occupied d–orbitals confer flexible coordination geometries, while the absence of d–d transitions suppresses non–radiative decay and promotes efficient ligand–centered (LC) emission, even in water [48, 49, 50]. These features, combined with their ease of tuning solubility and functionality via ligand modification or counterion exchange, make Tpy–Zn complexes a robust, versatile, and sustainable platform for multifunctional material design [51, 52, 53, 54, 55].

For instance, through non–covalent interactions, including π–π stacking, metal–metal bonding, and others, various planar pincer complexes with *penta*–coordination geometry readily self–assembly into supramolecular gels, offering great potential for visual molecular recognition via phase transition [56, 57]. In the case of *hexa*–coordinate motifs, like [R_1_–Tpy–Zn–Tpy–R_2_]^2+^, they serve as predictable assembly nodes for coordination polymers, metal–organic frameworks (MOFs), and supramolecular cages, thereby enabling diverse feasibility in photocatalysis [58], sensing [59, 60, 61], and light–emitting devices [62, 63]. Moreover, inexpensive, environmentally benign, and highly biocompatible Tpy–Zn complexes are readily incorporated into polymeric or hybrid matrixes to generate multifunctional composites, which are even capable of stabilizing ambient room–temperature phosphorescence (RTP), therefore exhibiting potential in information encryption, anti–counterfeiting [64], and responsive optical systems [65]. Although significant advances have been achieved, an overarching and critical review that systematically traces the progression from molecular design to smart functional materials, as well as their applications toward molecular recognition, catalysis and optical materials is still lacking. Herein, we compile design principles of Tpy–Zn complexes, the fabrication of functional materials, and representative applications in the aforementioned areas, emphasizing function–oriented design strategy and clear structure–property correlations, which may allow us to establish guiding concepts for the rational design and precise fabrication of next–generation smart Tpy–Zn–based materials and stimulate further innovation in this rapidly evolving discipline.

## 2 Molecular Design Strategies for Terpyridine Ligands and Corresponding Complexes

**FIGURE 2:**
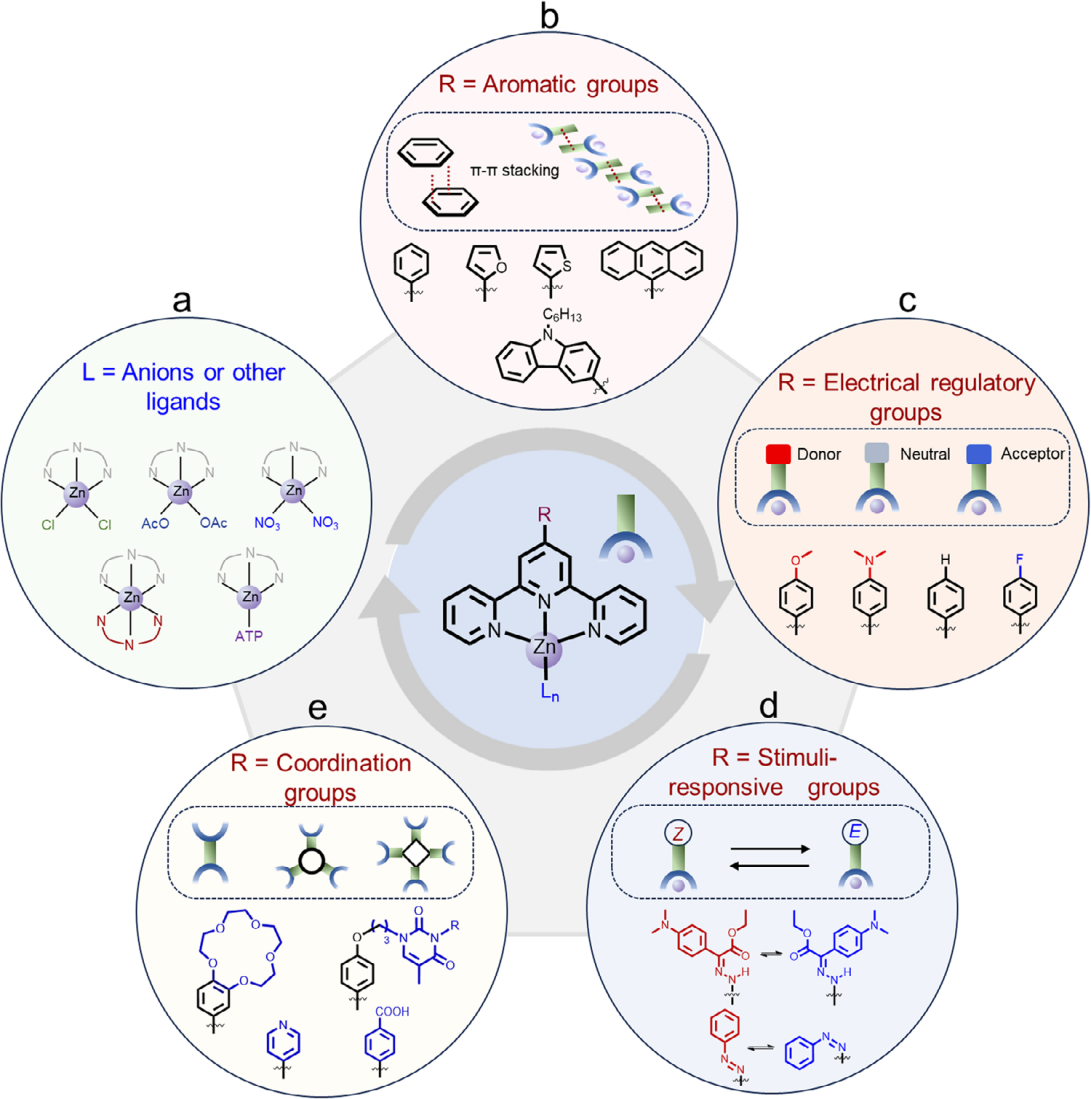
Main strategies to design Tpy ligands and corresponding Tpy–Zn complexes.

In the case of molecular structure and coordination geometry of Tpy–Zn complexes, two simple modification positions are readily apparent: auxiliary ligands/counterions (L) coordinated to the Zn(II) center, and the substituents (R) on the Tpy ligand scaffold. When substituents (R) on the Tpy are fixed, various counterions, including Cl^−^, Br^−^, I^−^, NO_3_^−^, OAc^−^, and OTf^−^, etc., could be introduced by anion–exchanging [65, 66], or replacement by additional Tpy ligands or other guest molecules like bipyridines, phenanthrolines as well as amino acids, nucleotides and even peptides [60, 61]. The exchanging strategy not only effectively alters solubility and aggregation behaviors of the complexes, but also modulates coordination geometry, possibly leading to the formation of [R_1_–Tpy–Zn–Tpy–R_2_]^2+^ motifs with different topologies (Figure 2a) [67]. Remarkably, R–modification of Tpy ligands provides a progressive approach to enrich structural and functional diversity of the target complexes. The most straightforward and practical approach is to introduce aromatic groups, which extend conjugation, promotes π–π stacking, and serves as the basic driving force for molecular self–assembly (Figure 2b) [56, 68]. Furthermore, incorporating electron–donating or electron–withdrawing R–substituents can regulate the charge distribution and donor/acceptor ability of the Tpy ligands, thereby tailoring the photophysical properties and coordination geometry of the complexes (Figure 2c) [64, 69]. Following this principle, stimuli–responsive motifs such as photo–isomerizable or redox–active groups are readily embedded to endow reversible optical or structural transformations under external triggers (Figure 2d) [65, 66]. Finally, R–substituents bearing additional coordination sites, including heteroatoms, carboxylates, or crown ethers, not only expand binding capability toward guest molecules but also allow the construction of multidentate Tpy ligands, leading to hierarchical assemblies with much sophisticated structures and tailored functions (Figure 2e) [70, 71, 72]. Consequently, these strategies highlight a systematic and hierarchical design flow, in which modifications at both the L and R sites lead to controllable structures and versatile functions of Tpy–Zn complexes.

### 2.1 Modification of L Motifs

**FIGURE 3:**
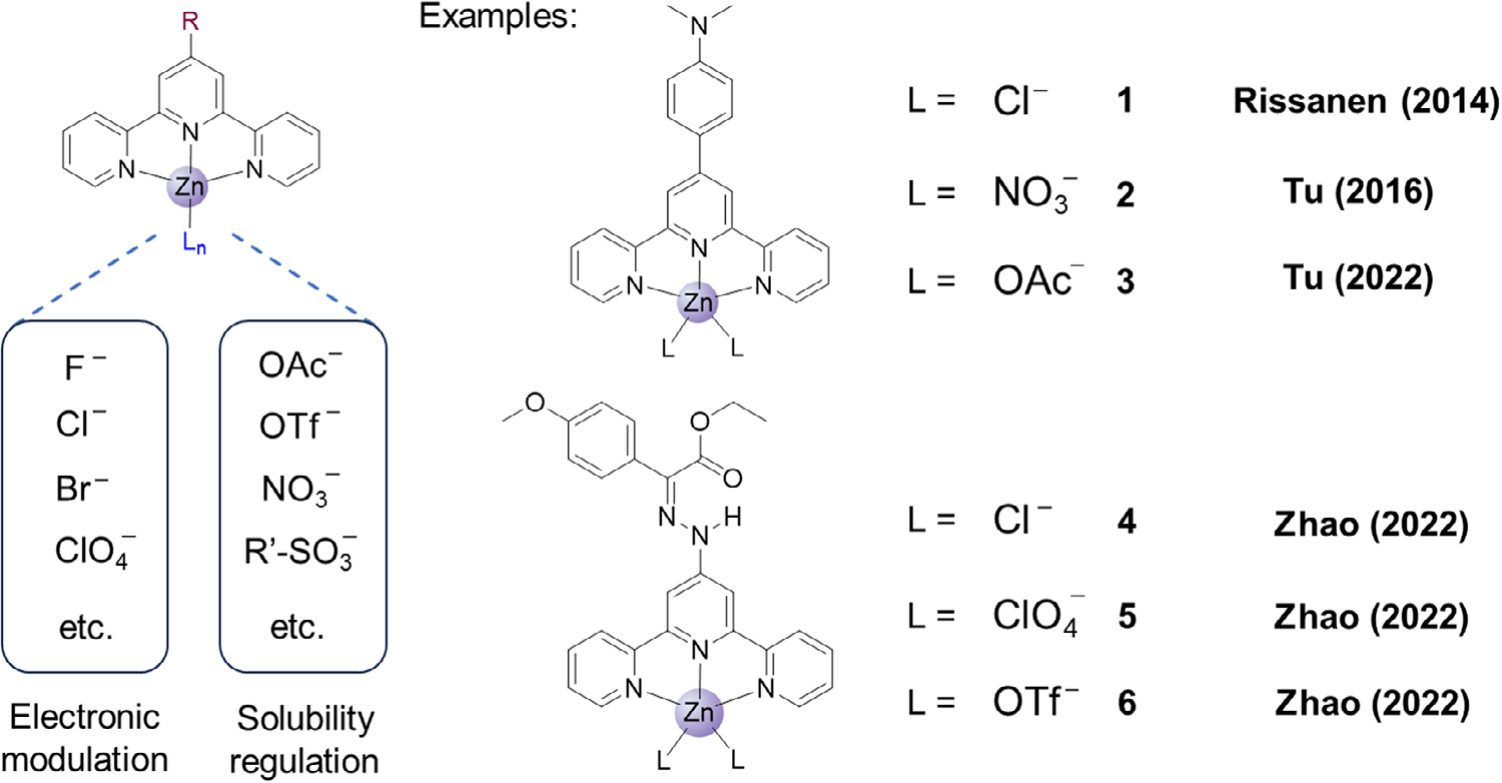
Schematic illustration and examples of molecular design strategies of Tpy–Zn complexes with various electron–regulating and/or solubility–modulating counterions anions (L).

Modification of L motifs provides a versatile handle to tailor coordination geometry, solubility, and other functionality of the Tpy–Zn complexes. For example, Rissanen (2014) [73] and Tu (2016 and 2022) [61, 69] reported a series of Tpy–Zn complexes containing different counterions (Figure 3). They found that halide anions (Cl^−^, Br^−^, I^−^) and the easily dissociable NO_3_^−^ promoted intermolecular assembly, leading to the formation of supramolecular gels with distinct morphologies and unique optical properties. In contrast, the incorporation of hydrophilic anions (OAc^−^ and OTf^−^) markedly enhanced solubility in aqueous media, facilitating the possibility to access water–compatible coordination systems and/or stimuli–responsive luminescent probes [69]. Furthermore, Zhao and coworkers (2022) synthesized a series of Tpy–Zn complexes with Cl^−^, ClO_4_^−^, and OTf^−^ counterions, which exhibited distinct fluorescence emission (Figure 3) [65]. Density functional theory (DFT) calculations confirmed that the counterions modulated the HOMO–LUMO energy gap of the Tpy–Zn complexes, thereby tuning their electronic properties and emission colors. Therefore, these results demonstrate that counterion–exchange strategy provides a simple yet powerful tool for controlling the aggregation behavior, solubility, and electronic structure of Tpy–Zn complexes, establishing a structure–property correlation protocol for predicting or designing molecules and composites with desired supramolecular and photophysical properties.

**FIGURE 4:**
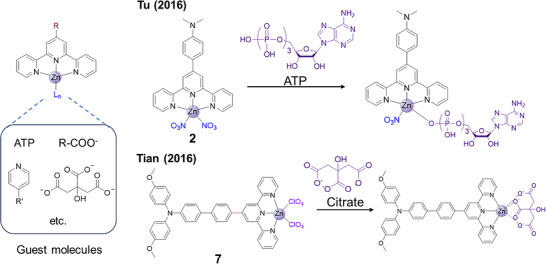
Schematic illustration and examples of molecular design strategies of Tpy–Zn complexes by coordination with as guest molecules (L), including ATP, carboxylates, nitrogen donors, citrate, and etc.

Besides simple counterions, other guest molecules containing N, P, or O donor atoms, such as pyridines, carboxylates, citrates, amino acids, nucleotides, and other biomolecules, can also be readily coordinated to the Zn(II) center. In 2016, the Tu group reported that coordination of adenosine triphosphate (ATP) or homologues with Tpy–Zn complexes led to a remarkable enhancement in fluorescence intensity (Figure 4) [61]. Similarly, in 2016, Tian and co–workers demonstrated that citrate binding to Tpy–Zn complex significantly increased emission intensity (Figure 4), confirming that guest–induced ligand substitution can modulate the photophysical behavior of the resulting Tpy–Zn complexes [74]. These studies therefore highlight that guest–induced coordination not only diversifies the supramolecular architectures but also enables the tuning of luminescent properties, laying the groundwork for their potential applications in fluorescence–based visual recognition and sensing.

**FIGURE 5:**
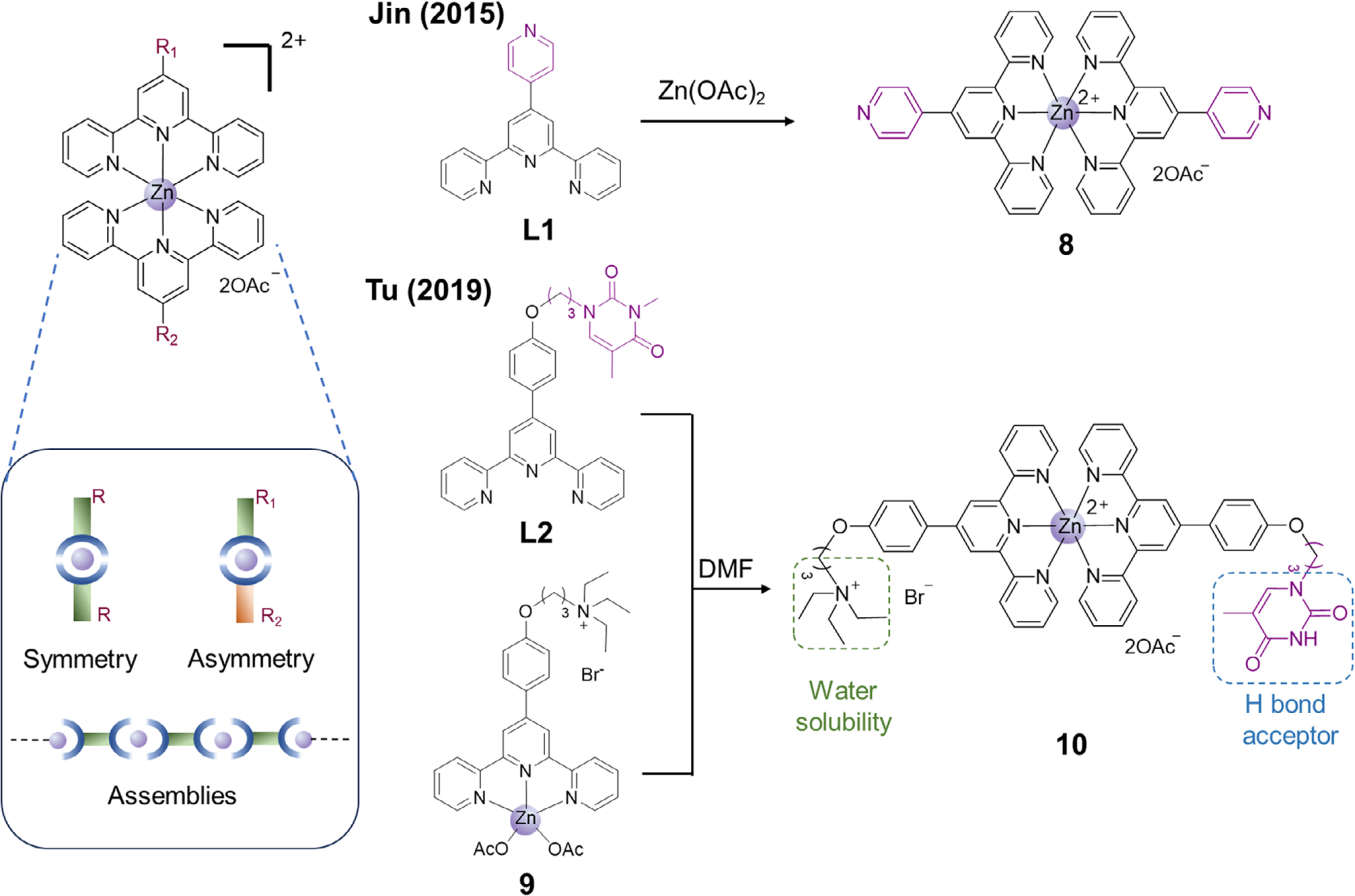
Schematic illustration and examples of molecular design strategies of [R_1_–Tpy–Zn–Tpy–R_2_]^2+^ with *hexa*–coordinated geometry.

Moreover, when L was substituted by the second Tpy ligand (Tpy–R_2_), the resulting robust *hexa*–coordinate [R_1_–Tpy–Zn–Tpy–R_2_]^2+^ motifs serve as predictable nodes for accessing extended molecular assemblies. In 2014, Jin and colleagues synthesized ligand **L1**, which coordinated with Zn(OAc)_2_ under controlled stoichiometry and reaction temperature to yield the symmetric *hexa*–coordinate complex **8** (Figure 5) [75]. Owing to the presence of pyridyl substituents at both termini and the thermodynamic stability of the [Tpy–Zn–Tpy]^2+^ unit, complex **8** constitutes a robust structural motif that can act as a bridging ligand for the construction of extended supramolecular structures. Subsequently, asymmetric six–coordinate complexes have also been demonstrated. In 2019, Tu and co–workers synthesized a thymine–functionalized Tpy ligand **L2** and a quaternary ammonium–containing complex **9** (Figure 5) [67]. Subsequent coordination between these two components yielded the asymmetric complex **10**, in which the thymine unit at one terminus enables multiple hydrogen–bonding interactions with guest molecules, while the quaternary ammonium group at the other end significantly enhances water solubility. This dual functionality highlights a rational design strategy for tailoring asymmetric *hexa*–coordinate complexes toward aqueous‐phase molecular recognition and sensing.

### 2.2 Modification of R Substitutes

Besides alternating the counterions or ligands bound to Zn(II) centers, rational modification can also be achieved by modifying the R substituents of the pincer ligands. These variations introduce diverse functional substituents with different structure, hindrance and electronic properties, which can be simply classified into four types: (hetero)aromatic groups, electronic regulating groups, stimuli–responsive units, and substituents with auxiliary chelation sites.

#### 2.2.1 R = (Hetero)Aromatic Groups

In Tpy–M coordination systems (M = Zn, Pd, Pt, Au, Cu, etc.), the introduction of (hetero)aromatic substituents is one of the most widely adopted molecular design strategies [30, 32, 76, 77]. This approach, originally developed across different Tpy–M frameworks, has been especially effective in the case of Tpy–Zn complexes. Incorporating aromatic rings extends π–conjugation and promotes intermolecular interactions, which support ordered self–assembly and the formation of functional soft materials. To date, a broad range of aromatic and heteroaromatic groups, such as phenyl, naphthyl, thiophene, furan, and carbazole, have been introduced into Tpy–Zn ligands, reflecting the flexibility and synthetic accessibility of this modification strategy.

**FIGURE 6:**
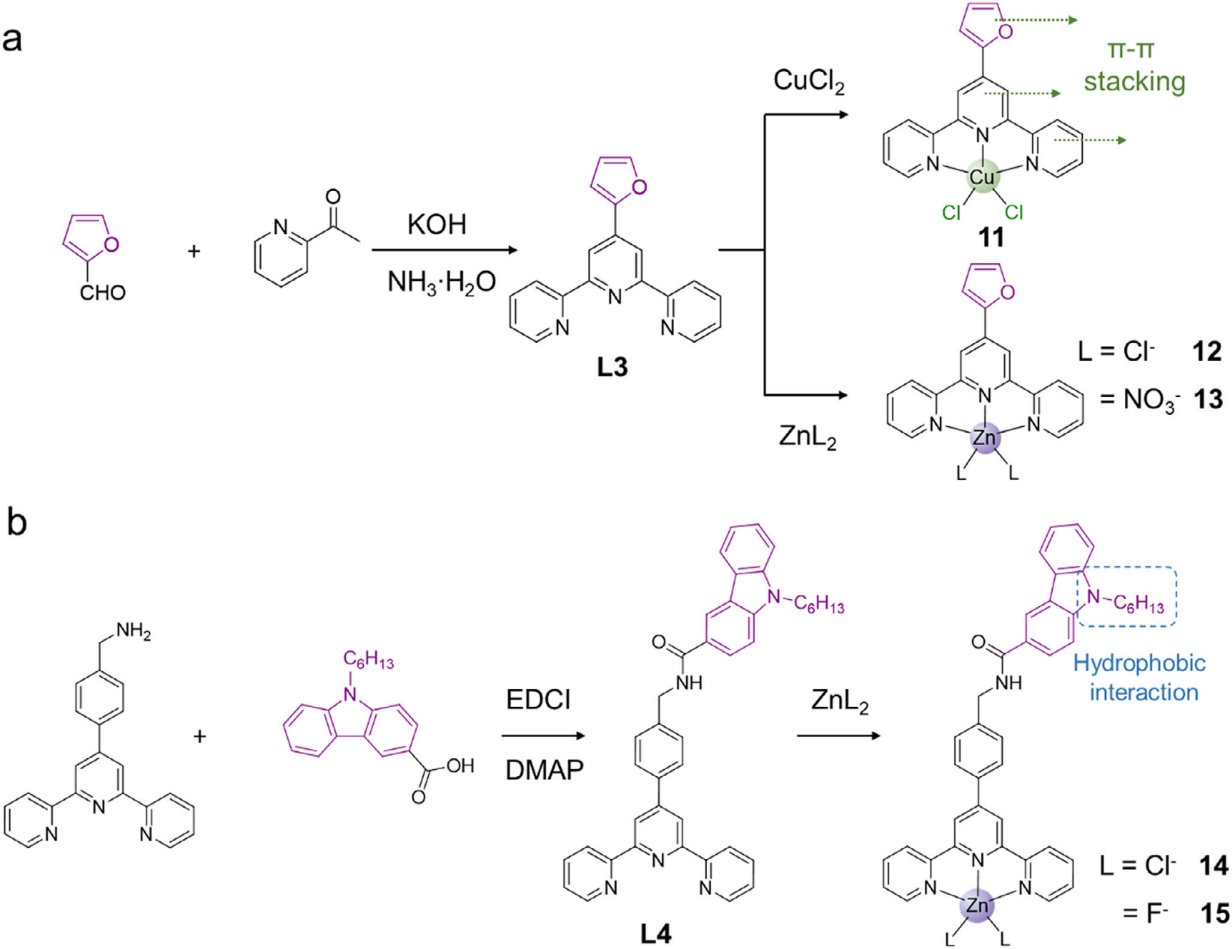
Representative examples of Tpy ligands bearing heteroaromatic substituents. (a) Synthesis of furan–substituted Tpy ligand **L3**, and corresponding complexes **11**–**13** formed by coordination CuCl_2_, ZnCl_2_ and Zn(NO_3_)_2_, respectively [56, 78]. (b) Synthesis of carbazole–functionalized Tpy ligand **L4** by amide condensation, and corresponding complexes **14** and **15** after coordination with ZnCl_2_ and ZnF_2_, respectively [68].

A representative example was reported by Tu and co–workers in 2014, they synthesized a furan–substituted Tpy ligand **L3** through a one–step condensation of *p*–substituted aromatic aldehydes with 2–acetylpyridine [56, 78]. This straightforward condensation route represents one of the simplest and most widely adopted synthetic methods for synthesizing Tpy ligands, offering an easy approach to access functionalized pincer ligands with various aromatic fragments. After coordination of with CuCl_2_, ZnCl_2_ or Zn(NO_3_)_2_, **L3** is readily to yield pincer complexes **11**–**13**, respectively (Figure 6a). Although conjugated furan ring reinforced the plausible intermolecular π–π interactions between the pincer complexes, the choice of counterion significantly altered their solubility, especially in water, leading to distinct assembly behaviors.

In contrast, Zhao and co–workers employed a modular approach to construct a carbazole–functionalized Tpy ligand **L4**. Unlike the classical one–step condensation approach, a carbazole derivative bearing a hexyl side chain was first functionalized with an appropriate carboxyl group, which was then connected to the pincer scaffold via an amide bond (Figure 6b) [68]. After coordination with ZnCl_2_ and ZnF_2_, the corresponding pincer complexes **14** and **15** were readily obtained, respectively. The bulky carbazole substituent also promotes π–π stacking, along with hydrophobic interactions caused by the appended hexyl chains, further stabilizes the supramolecular assembly system. This modular approach allows the introduction of structurally complicated aromatic motifs, thereby extending the diversity of terpyridine ligands, though significant synthetic efforts are required.

#### 2.2.2 R = Electronic Regulating Groups

While conjugated (hetero)aromatic groups primarily accelerate the supramolecular assembly process due to enhanced weak intermolecular interactions. Other refinements of the pincer ligands can also be achieved by introducing electron–donating (D) or electron–accepting (A) groups. These substitutions directly alter the electronic distribution of the whole ligand, thereby reshaping the coordination environment of Zn(II) centers and tuning the overall electronic character of the resulted complexes. Notably, the Tpy–Zn fragment is generally regarded as an electron acceptor, thus, when electron–donating substituents are introduced at the 4’–position of Tpy, the resulting complexes characterize as a typical D–π–A conjugated configuration. This structural arrangement facilitates charge transfer, improves electronic communication across the whole molecule, and may endow the complexes with enhanced optoelectronic properties. Consequently, numerous studies have focused on incorporating electron–donating groups into the Tpy ligands.

**FIGURE 7:**
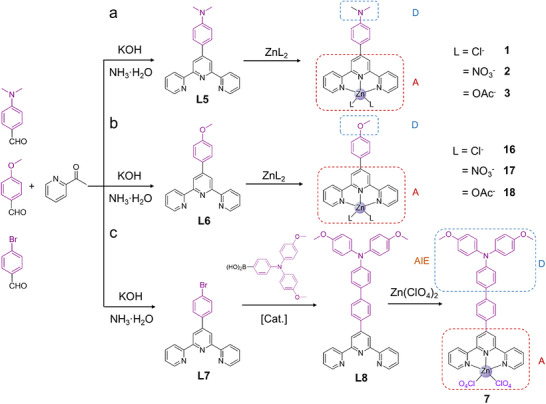
Synthesis of ligands **L5**, **L6** and corresponding complexes **1–3** and **16–18** containing (a)–NMe_2_ and (b)–OMe groups after coordination with ZnCl_2_ [73], Zn(NO_3_)_2_ [61] and Zn(OAc)_2_ [69]. (c) Synthesis of ligands **L7**, **L8** and complex **7** after coordination with Zn(ClO_4_)_2_ [74].

Representative progress has been made with the introduction of strong electron–donating groups like –NMe_2_. Both the Rissanen (2014) [73] and Tu groups (2016 and 2022) [61, 69] synthesized the –NMe_2_ substituted Tpy ligand **L5** via the classical one–step condensation approach. Coordination of **L5** with ZnCl_2_ afforded complex **1** in the work of Rissanen's team, whereas Tu and co–workers employed Zn(NO_3_)_2_ and Zn(OAc)_2_ to produce complexes **2** and **3** (Figure 7a). Benefiting from their D–π–A feature, both complexes exhibited favorable luminescent properties. Nevertheless, the choice of counterion played a decisive role in further modulating their performance: complexes **1** and **2** displayed enhanced aggregation ability and readily formed supramolecular gels, whereas complex **3** containing OAc^−^ showed good water solubility, highlighting its potential for luminescent materials in aqueous media. The aforementioned works confirm the contribution of the –NMe_2_ group to the D–π–A structures, as well as the key role of counterions in impacting on their solubility and molecular assembly behaviors. With this insight, Tu et al. further synthesized a –OMe substituted ligand **L6** via the same one–step condensation approach [64]. After coordination with ZnCl_2_, Zn(NO_3_)_2_ and Zn(OAc)_2_, D–π–A complexes **16–18** were furnished (Figure 7b). Distinct from the –NMe_2_ analogues, these –OMe substituted complexes displayed multi–stimuli–responsive luminescence in aqueous media. This behavior underscores the versatility of electron–donating substituents in fine–tuning the optoelectronic behaviors of Tpy–Zn complexes.

A further advance in this topic was reported by the Tian group in 2016, who introduced more complex donor fragments by the modular synthetic route [74]. With 4’–bromo–substituted Tpy **L7**, a multi–phenyl substituted pincer ligand **L8** was readily produced via Suzuki coupling reaction with diverse aromatic boric acids (Figure 7c). The introduction of these aromatic groups was particularly striking, which further endowed aggregation–induced emission (AIE) characteristics into the resulting ligands and/or complexes, thereby circumventing the aggregation–caused quenching (ACQ) phenomena, commonly observed in conventional π–conjugated organic/organometallic molecules. Complex **7**, formed from **L8** and Zn(ClO_4_)_2_, in which ClO_4_^−^ anion with weak coordination ability facilitates guest molecules binding to the metal center after substitution. Combining the tailored synthetic strategy, AIE–active donor group, and counterion selection, this example exemplified how careful molecular design can simultaneously optimize emission behavior and create opportunities for developing responsive molecular sensor.

#### 2.2.3 R = Stimuli–Responsive Units

Additionally, aromatic substitution and electronic regulation could be readily applied to incorporate other substituents that impart stimuli–responsive feature to the Tpy ligands. For example, photoactive groups, like azobenzene, hydrazone, Schiff–bases, etc., which undergo reversible structural transformation under light irradiation, are able to directly tune photophysical properties of the resulting Zn(II) pincer complexes. After embedding the stimuli–responsive moieties into the Tpy ligands, the complexes not only retain the advantages of conjugation and electronic modulation but also respond adaptively to external triggers, thereby extending their applicability.

**FIGURE 8:**
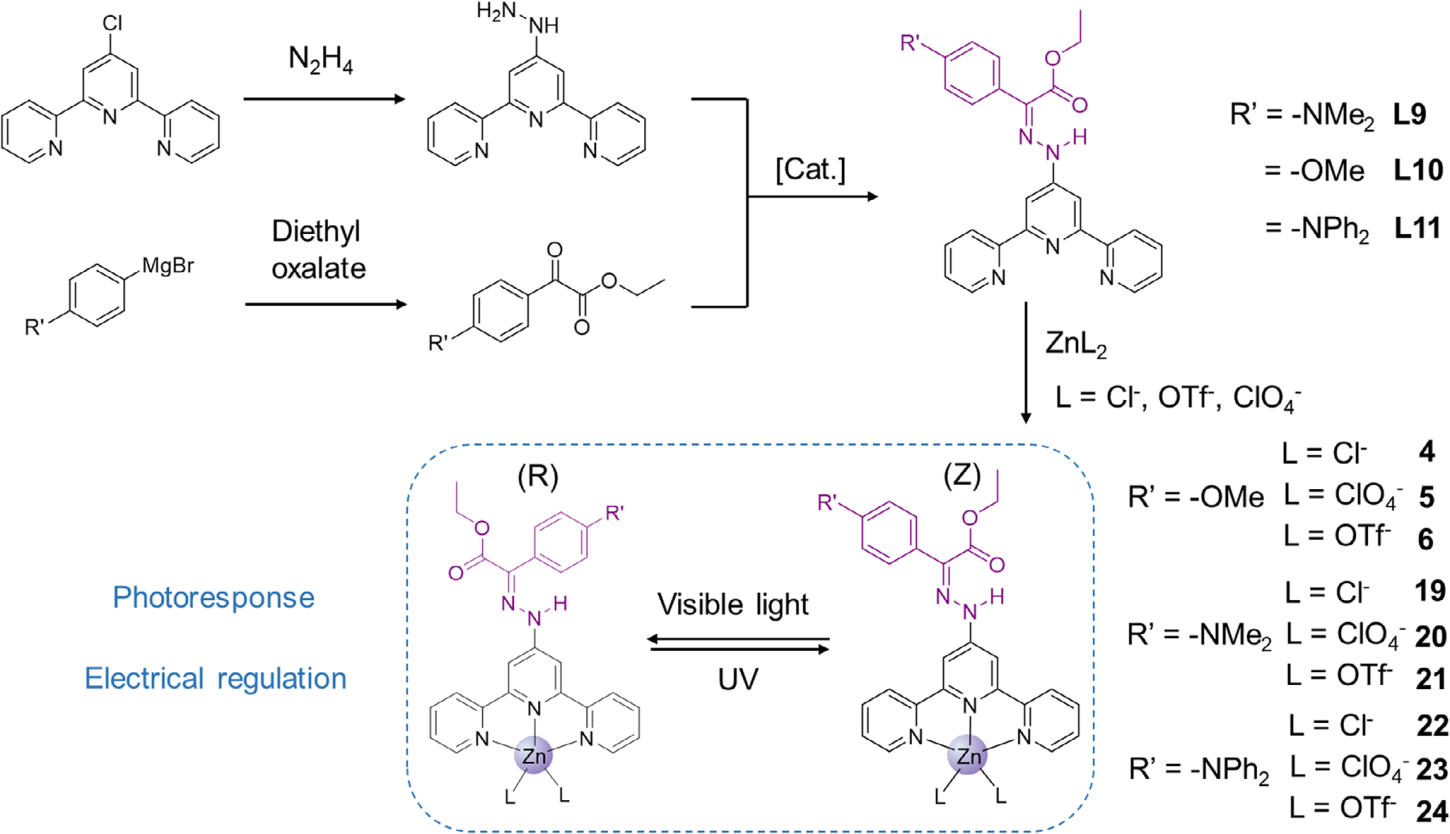
Schematic representation of stimuli–responsive Tpy ligands and their Zn(II) complexes. Dynamic imine–containing ligands **L9–L11** [65, 66] were synthesized with different electronic substituents, and subsequent coordination with Zn(II) salts bearing various counterions afforded complexes **4–6**, and **19**–**24**, respectively [65, 66].

In 2022 and 2023, Zhao and co–workers designed and synthesized two Tpy ligands **L9** and **L10** containing hydrazone and other electronic tunable substituents (Figure 8) [65]. Among them, ligand **L9** bears –NMe_2_ substitution, whereas **L10** contains –OMe group. Furthermore, they also introduced a diphenylamino (–NPh_2_) group and produced **L11** [66]. These ligands are readily coordinated with various salts ZnL_2_ (L = Cl^−^, OTf^−^ and ClO_4_^−^) and generate complexes **4–6** and **19–24**, respectively. The incorporation of hydrazone units imparted photo–switchable isomerization behavior to the resulting pincer complexes, enabling reversible structural isomerization under light irradiation and thus endowing the complexes with controllable luminescence. Importantly, the emission properties were further fine–tuned by both the electron–donating strength of the R substituents (–NMe_2_, –OMe, –NPh_2_) and the nature of the counterions, allowing systematic modulation of emission wavelengths. These studies demonstrated how to integrate photo–responsive motifs with electronic and anion regulation and create Tpy–Zn complexes which are capable of switching multicolor emission and offering great promise for applications in rewritable optical materials and information storage.

#### 2.2.4 R = Coordination Groups

In addition to aromatic substitution, electronic regulation, and stimuli–responsive motifs, another effective strategy is introducing extra bonding sites, like carboxylates, pyridine or imidazole fragments, crown ethers, etc., to the pincer skeletons. These groups provide complementary functionality beyond the intrinsic coordination ability of the Tpy unit to the central Zn(II) ion. By enabling secondary interaction with guest molecules and/or other metal ions, these bonding sites further enrich the structural diversity of the Tpy–Zn complexes and offer a new approach for constructing predictable higher–order assemblies.

**FIGURE 9:**
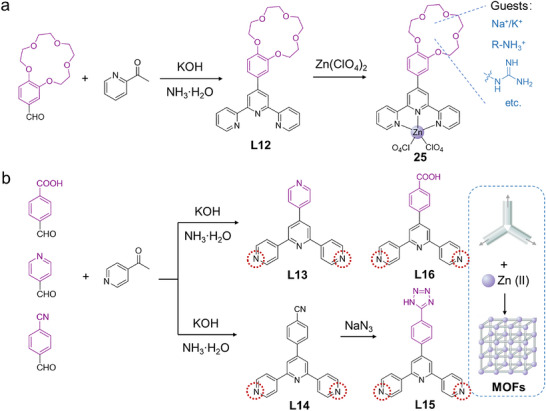
Tpy ligands with additional coordination groups. (a) Crown ether–functionalized ligand **L12** afforded complex **25** with Zn(ClO_4_)_2_ [70]. (b) Ligands **L13** [71], **L15** [79], and **L16** [72] obtained by altering the position of the N donor atom.

In 2011, Wu and co–workers reported the crown ether–functionalized Tpy ligand **L12** and its corresponding complex **25** after coordination with Zn(ClO_4_)_2_ (Figure 9a) [70]. The crown ether fragment served as an auxiliary binding site, enabling interactions with a wide range of guest molecules, including alkali metal cations such as Na^+^, K^+^, organic ammonium salts (R–NH_3_^+^), and guanidinium derivatives. This design expanded the functionality of the simple Tpy–Zn coordination complexes to serve as versatile platforms in host–guest molecular recognition.

Modifying the positions of nitrogen atoms within the Tpy skeleton represents another viable strategy for ligand design. Starting with 4–acetylpyridine, Zhao (2019, **L13**) [71], Wang (2019, **L15**) [79], and Tutar (2025, **L16**) [72] realized the synthesis of para–pyridine substituted Tpy ligands using the condensation approach (Figure 9b). These alterations shifted the orientation and accessibility of the coordination sites, favoring outward–directed binding modes. Such a modification is particularly useful for constructing extended coordination networks, making these ligands highly suitable for the fabrication of MOFs or coordination polymers. While the specific network structures may vary, the essential design principle lies in exploiting donor atom geometry to enable robust multidirectional coordination.

**FIGURE 10:**
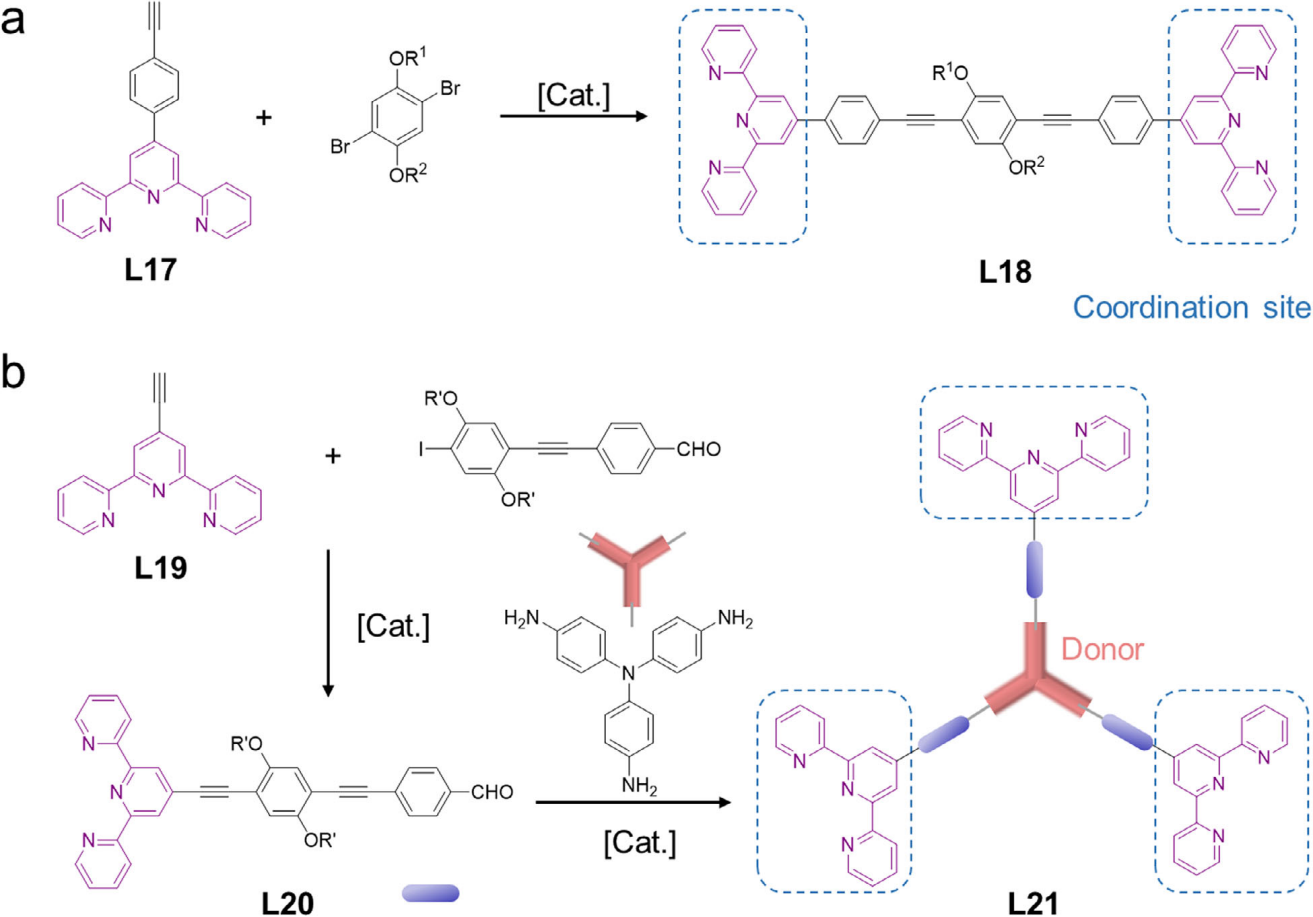
Functional multitopic pincer ligands (a) **L18** with two Tpy fragments [80]. (b) **L21** with three Tpy fragments [81].

Another reported approach involves incorporating multiple Tpy coordination sites within a single ligand, which greatly expands the structural complexity and coordination assembly potential for the resulting structure. In 2008, Schubert and colleagues reported **L18**, prepared by cross–coupling of alkyne–functionalized Tpy **L17** and dibromoarene in 2:1 ratio (Figure 10a) [80]. The resulting **L18** bears two Tpy fragments oriented opposite to each other at the terminals of the molecule, both capable of binding to various metal ions. This linear arrangement makes **L18** as a versatile building block for the construction of 1D coordination polymers. Following a similar approach, in 2020 Ghorai and co–workers designed ligand **L21** by condensing Tpy aldehydes **L20** with tris(4–aminophenyl)amine (TAPA) via an imine formation reaction (Figure 10b) [81]. The incorporation of three Tpy units oriented in triangle directions provided a ligand framework well–suited for generating 2D network–like coordination polymers upon metal coordination.

**FIGURE 11:**
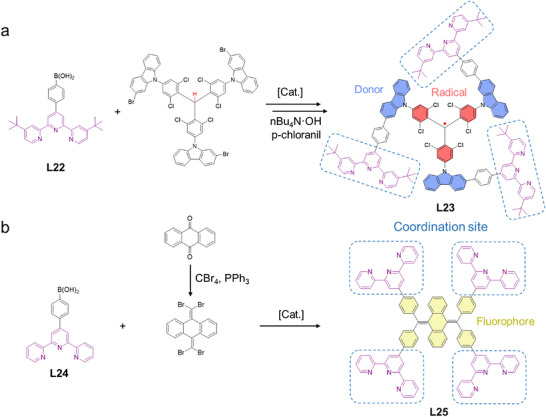
Tpy ligands containing multiple coordination sites. (a) Ligand **L23** formed by three Tpy fragments and triphenyl radical core [82]. (b) Ligand **L25** carries four Tpy fragments and fluorophore core [62].

In contrast to the tridentate ligands with defined geometry that favor planar 2D coordination propagation, Wang and Abdurahman group proposed a distinct molecular design strategy by integrating functional radical units into multitopic Tpy ligands. In 2023, they synthesized a tridentate Tpy ligand **L23** featuring three Tpy arms radiating from a central carbazole core and a persistent tris(2,4,6–trichlorophenyl)methyl (TTM) radical unit (Figure 11a) [82]. This ligand was prepared from Tpy precursor **L22** and a carbazole precursor via a Suzuki coupling reaction followed by deprotection and oxidation to introduce the TTM radical center. The electron–donating carbazole motif provides the directing angles and plays a key role in adjusting the electronic density of **L23**. The incorporation of the open–shell radical species not only expanded the spatial orientation of coordination sites, enabling the potential construction of more complex 3D architectures, but also imparted redox activity and spin–related functionality to the molecule. Such a dual–attribute design provides a versatile platform that combines structural directivity with radical–based optical and catalytic potential.

In 2022, the Wang group synthesized a tetratopic Tpy ligand **L25** via a Suzuki coupling reaction between dihydroanthracenyl tetrabromide and a boronic acid–functionalized Tpy precursor **L24** (Figure 11b) [62]. The incorporation of the rigid and π–conjugated dihydroanthracene core not only provided four spatially distributed Tpy arms for multidirectional coordination but also introduced an emissive chromophore into the molecular backbone. This design endowed **L25** with dual advantages of structural complexity and photophysical functionality, enabling the subsequent assemblies to serve as promising candidates for luminescent supramolecular materials.

**FIGURE 12:**
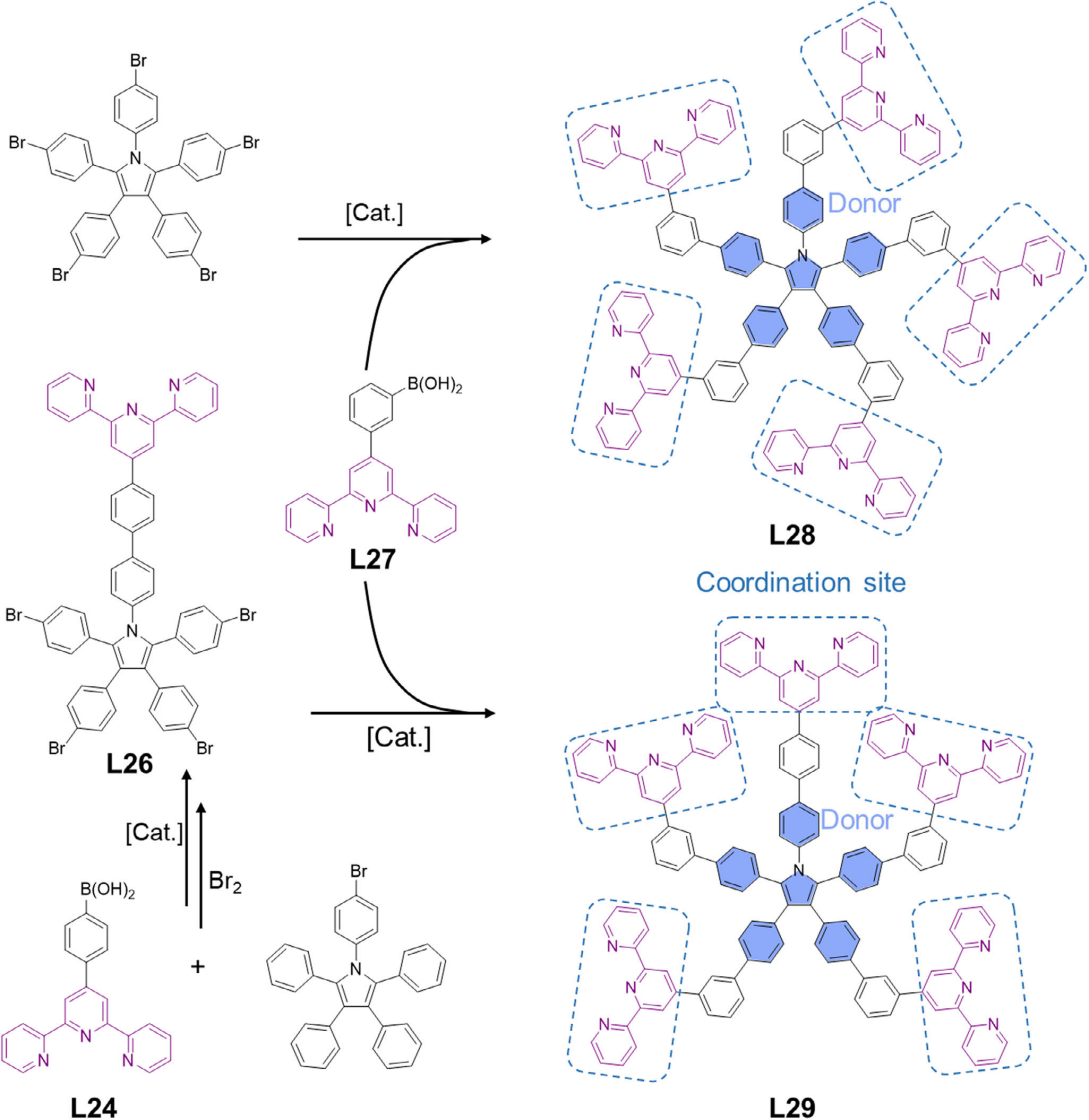
Functional Tpy ligands **L28** and **L29** containing five Tpy fragments [83].

Based on these advances, in 2025 Li, Liu, and Wang reported two pentatopic Tpy ligands containing five Tpy fragments (**L28** and **L29**, Figure 12), further enriching the design scope of multitopic coordination systems [83]. Ligand **L28** was synthesized through a Suzuki coupling reaction between 1,2,3,4,5–pentakis(4–bromophenyl)–1H–pyrrole and the boronic acid–functionalized Tpy precursor **L27**. In a parallel route, ligand **L26** was first obtained by coupling the boronic acid–functionalized Tpy ligand **L24** with 1–(4–bromophenyl)–2,3,4,5–tetraphenyl–1H–pyrrole, followed by bromination, and its subsequent coupling with **L27** afforded ligand **L29**. Both ligands feature five Tpy units arranged in multiple directions, which coordinate with Zn(II) to generate intricate supramolecular cages. Therefore, these studies highlight the critical link between ligand design and coordination assembly. They demonstrate that integrating multiple Tpy units into a single molecule is a powerful strategy for constructing higher–order supramolecular architectures.

The molecular design of Tpy–Zn complexes focuses on tailoring the Zn–chelating ligands or counterions (L) along with the substituents (R) on the Tpy scaffold. The L site primarily governs properties such as solubility, coordination geometry, and electronic distribution. In contrast, the R site enables extended conjugation, tailored electronic structures, dynamic response, and additional binding sites. These approaches illustrate how to govern the photophysical properties and the modes of supramolecular assembly at molecular level. With these strategies, Tpy–Zn complexes can be further assembled into diverse material systems, in which the principles of molecular design are translated into functional architectures with broad applicability.

## 3 Construction of Functional Materials or Composites From Simple Tpy–Zn Complexes and Their Derivatives

With a variety of Tpy–Zn complexes and derivatives synthesized by the aforementioned molecular design strategies, the resulting molecules can be readily fabricated into various functional materials with diverse components, architectures and morphologies. By judiciously controlling intermolecular interactions, coordination connectivity, and other integration approaches, Tpy–Zn units have been organized into soft supramolecular gels, crystalline MOFs, coordination polymers, supramolecular cages/frameworks, and various hybrid composites. Each material retains the intrinsic characteristics of the Tpy–Zn coordination core while exhibiting emergent properties conferred by its supramolecular architecture, such as tunable luminescence, selective molecular recognition, catalytic activity, and environmental adaptability. These diverse construction approaches enable the assembly of functional materials from Tpy–Zn molecules with different structure, morphology and components for facilitating their applications in sensing, imaging, catalysis and related fields.

### 3.1 Supramolecular Gels

**FIGURE 13:**
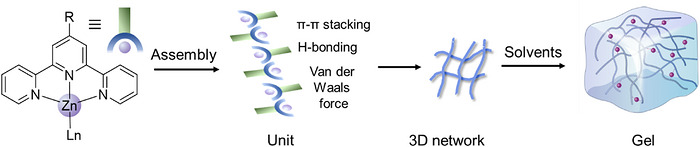
Schematic diagram of preparing supramolecular hydrogels from Tpy–Zn complexes.

Supramolecular gels represent one of the most important types of soft materials, which have texture and appearance that are similar to jelly [84, 85]. Their essence is that small molecules (also known as gelator, generally less than 2 wt%) initially arrange into 1D assemblies in an appropriate solvent under the drive of a number of weak non–covalent interactions (such as coordination interactions, π–π stacking interactions, metal–metal interactions, hydrogen bonds, hydrophobic interactions, dispersion force, and etc.), and then undergo subsequent cross–linking to form a 3D networks. The solvent molecules are encapsulated and fixed in the matrix, thereby forming the gel (Figure 13) [84, 85, 86, 87].

**FIGURE 14:**
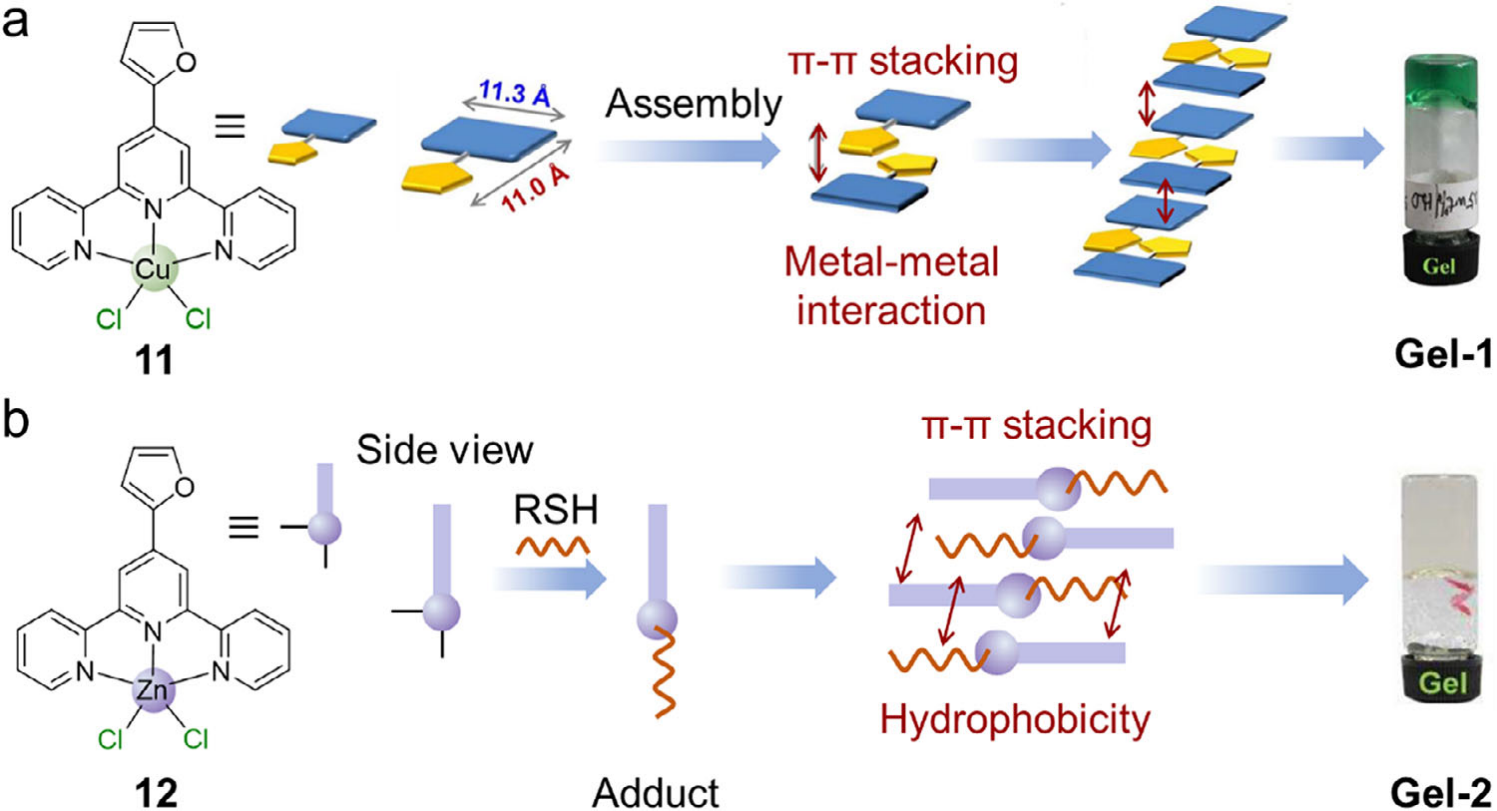
(a) Supramolecular **Gel–1** formation process of complex **11** and the coordination–blocking mechanism disrupting gelation. Reproduced with permission [78]. Copyright 2014, Royal Society of Chemistry. (b) Formation of a RSH induced supramolecular **Gel–2** from complex **12** via Tpy–Zn coordination and π–π stacking interactions. Reproduced with permission [56]. Copyright 2014, Royal Society of Chemistry.

Thanks to the various intermolecular interactions within the pincer skeletons, obvious advancements have been achieved in supramolecular gels fabricated by Tpy–metal complexes in recent decades. Tu and co–workers have conducted a systematic study on the synthesis, fabrication and application of organometallic supramolecular gels derived from Tpy–Zn complexes. In 2014, they reported a Tpy–Cu complex **11** that could spontaneously form a green supramolecular hydrogel (**Gel–1**) at an ultralow critical concentration of 0.25 wt% (Figure 14a) [78]. The gelation relied on cooperative π–π stacking and Cu···Cu interactions, but the system suffered from fluorescence quenching and potential cytotoxicity, which limited its applicability in bioimaging and transparent optical systems. These drawbacks triggered them to explore alternative metal cations like Zn(II), which retains the structural characteristics of Cu analogues, but exhibits superior photophysical properties and biocompatibility due to low toxicity.

To address these limitations, complex **12** was synthesized by simply coordinating the Tpy ligand **L3** with ZnCl_2_. However, due to its poor water solubility, it failed to form a gel under identical conditions (Figure 14b) [56]. Upon introducing cysteine, a water–soluble amino acid bearing a thiol group, the Zn (II) complex transformed into a soluble adduct and successfully self–assembled into a hydrogel (**Gel–2**). Gelation in this system was driven by π–π stacking, Zn···Zn interactions, hydrogen bonding and hydrophobic interaction caused by the cysteine residues. This study not only highlights the effectiveness of ligand–level modification in tuning solubility and assembly behavior but also exemplifies an additive–assisted gelation strategy, in which an external guest molecule acts as a structural modulator to trigger gel–network formation.

**FIGURE 15:**
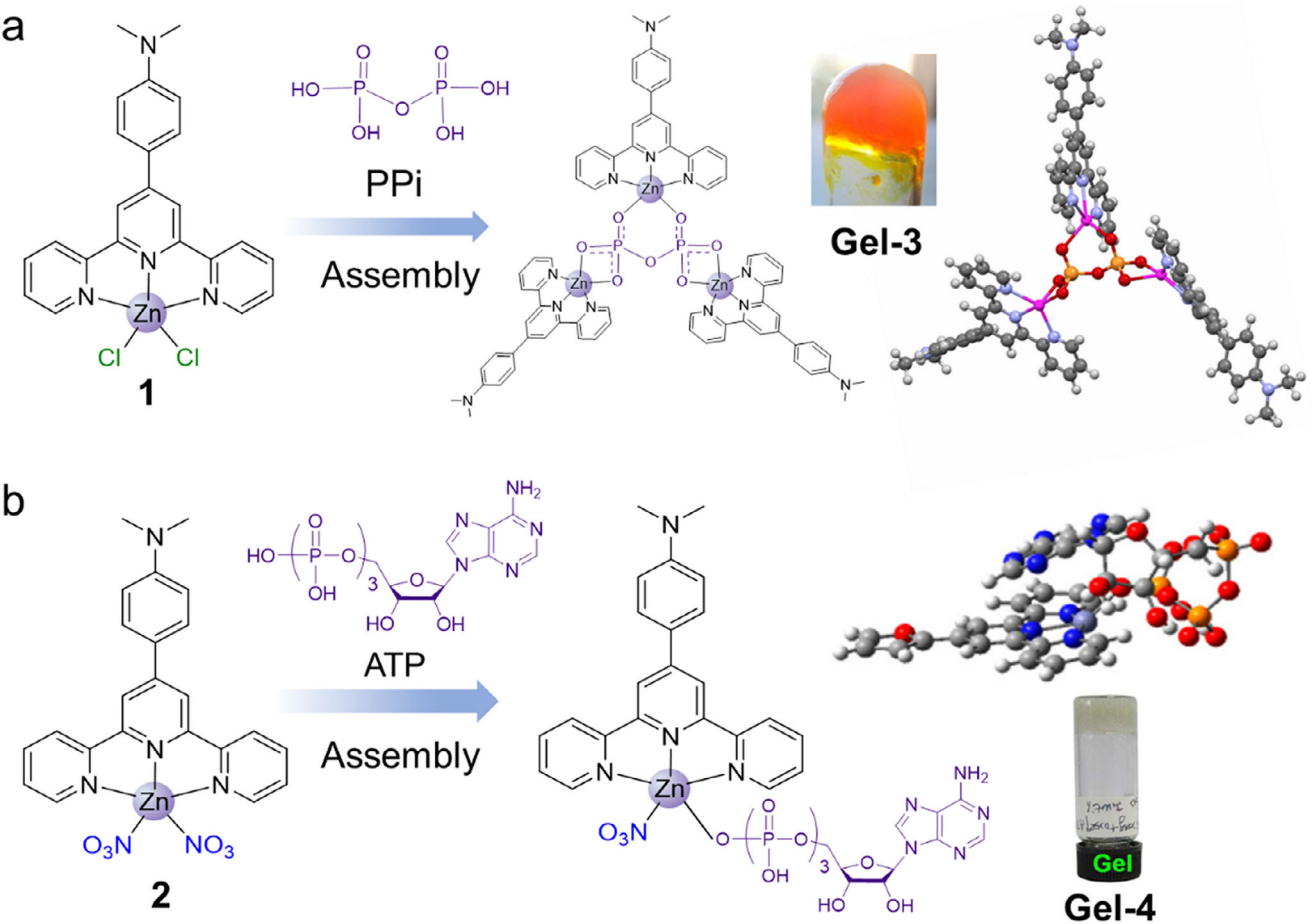
(a) Schematic assembly of complex **1** with PPi. Reproduced with permission [60]. Copyright 2014, American Chemical Society. (b) Schematic assembly of complex **2** with ATP. Reproduced with permission [61]. Copyright 2016, American Chemical Society.

Extending this concept, Rissanen and co–workers reported a pyrophosphate (PPi)–responsive pincer complex **1** in which the Tpy core was functionalized with an electron–donating –NMe_2_ group at the 4′ position and Cl^−^ served as the counterion (Figure 15a) [60]. The complex **1** with a donor–π–acceptor (D–π–A) electronic structure that is favorable for luminescence. While complex **1** formed a non–fluorescent gel (**Gel–3**) under mildly acidic aqueous conditions, the addition of PPi dramatically enhanced the fluorescence intensity (approximately 66–fold) through an AIE effect. Mechanistic studies revealed that PPi coordination to the Zn(II) center induced trimeric adduct formation, which increased the degree of molecular aggregation and stabilized the gel network. This work demonstrates how external molecules can both initiate gelation and modulate functional optical properties.

Subsequently, Tu and co–workers investigated how counterions govern gelation behavior of the resulting pincer complexes. Initially, they synthesized complex **2** by replacing the Cl^−^ anion of complex **1** by NO_3_^−^ with much more weakly coordinating ability (Figure 15b) [61]. This counterion modification significantly altered reactivity toward phosphate–containing biomolecules. Upon the addition of adenosine triphosphate (ATP), oxygen atoms of phosphate were readily coordinated to Zn(II) centers, the resulting adducts further assembled into a robust hydrogel (**Gel–4**). This comprehensive study underscores the critical influence of counterion after ligand exchange, offering a versatile approach to selectively for gel after trigging by specific molecules. Moreover, the gel–sol phase transition or luminescence variation is induced by specific biomolecules, providing a promising platform for the visual discrimination and/or recognition of biologically relevant species.

**FIGURE 16:**
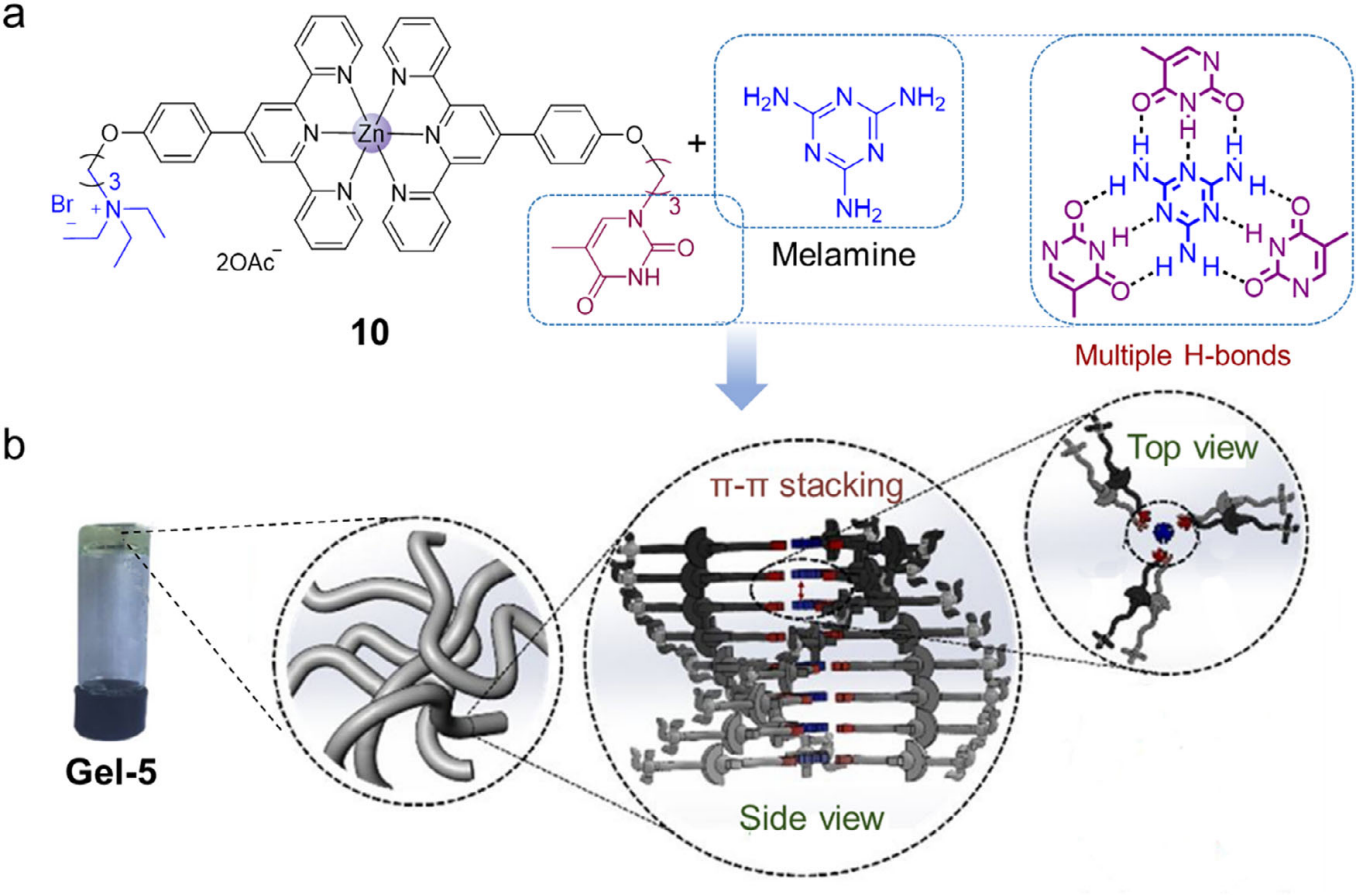
(a) Schematic diagram of the formation of multiple H–bonds between thymine in complex **10** and the guest molecule Melamine. (b) Schematic illustration of supramolecular material **Gel–5** formation between complex **10** and melamine, along with the proposed assembly mechanism. Reproduced with permission [67]. Copyright 2019, Elsevier.

To expand the scope of small–molecule–triggered gelation beyond direct coordination at the Zn(II) center, Tu group employed a ligand modification approach in 2019, in which the external trigger interacts with functional groups on the Tpy ligand rather than with the metal center itself [67]. They rationally synthesized a thymine–functionalized Tpy–Zn complex **10** to integrate complementary hydrogen–bonding sites with water–solubilizing quaternary ammonium groups (Figure 16a). The quaternary ammonium units enhanced solubility and promoted gelation, while the thymine moieties enabled specific guest recognition. Although complex **10** alone did not form a gel in aqueous media—likely due to steric hindrance and insufficient π–π stacking, the introduction of melamine, a hydrogen–bond–rich guest, induced directional assembly through multiple thymine–melamine hydrogen bonds, yielding a stable supramolecular hydrogel (**Gel–5**). Structural analyses confirmed the formation of a fibrous network driven by multiple hydrogen–bonds (Figure 16b). This ligand–functionalization approach, complementing earlier metal–centered gelation strategies, demonstrates how external small molecules can induce gelation through specific non‑covalent interactions.

From aforementioned studies, three main strategies have been adopted to access gelation from Zn(II) pincer complexes: (1) self–assembly (**Gel–1**), in which the complex spontaneously forms a supramolecular matrix; (2) guest–assisted gelation (**Gel–2**, **Gel–3** and **Gel–4**), where coordination of external guest molecules to Zn(II) centers results in adducts with enhanced aggregation tendency; and (3) functional groups–promoted gelation (**Gel–5**), where the introduction of groups capable of guest–ligand interactions, such as hydrogen–bonding donors or acceptors, gel–network formation was consequently mediated. In all three cases, gelation process modifies the physicochemical properties of the resulting soft materials, especially inducing pronounced luminescence alteration. These works suggest that Tpy–Zn complexes can be considered as versatile and tunable building blocks for supramolecular gel fabrication, owing to their modular ligand design, responsive coordination chemistry, and capacity to integrate diverse non–covalent interactions, thus offering a robust platform for precisely constructing functional soft materials.

### 3.2 Coordination Polymers

The nature of the R substituents on the Tpy ligand plays a crucial role in the formation of higher–order assemblies. As in supramolecular gel formation process, functional groups with abilities to form π–π stacking or interact with other guest molecules benefit cross–linking between gelator molecules and solvents leading to the formation of soft materials. When the R group has coordination ability, Tpy–Zn units can propagate in a head–to–tail way via metal–ligand chelating linkages to generate extended coordination polymers [88, 89]. By introducing rigid and directional coordination linkers through further ligand engineering, these assemblies can evolve toward long–range order, ultimately forming crystalline MOFs with well–defined porosity and topology [90, 91]. Herein, coordination polymers are therefore discussed in two categories: (1) amorphous coordination polymers, which exhibit network connectivity without long–range periodicity, and (2) crystalline metal–organic frameworks (MOFs), which possess ordered architectures with tunable structural parameters. This classification highlights the progress from disordered to crystalline structural properties and reflects the versatility of Tpy–Zn complexes as modular nodes for constructing functional coordination materials.

#### 3.2.1 Amorphous Coordination Polymer

**FIGURE 17:**
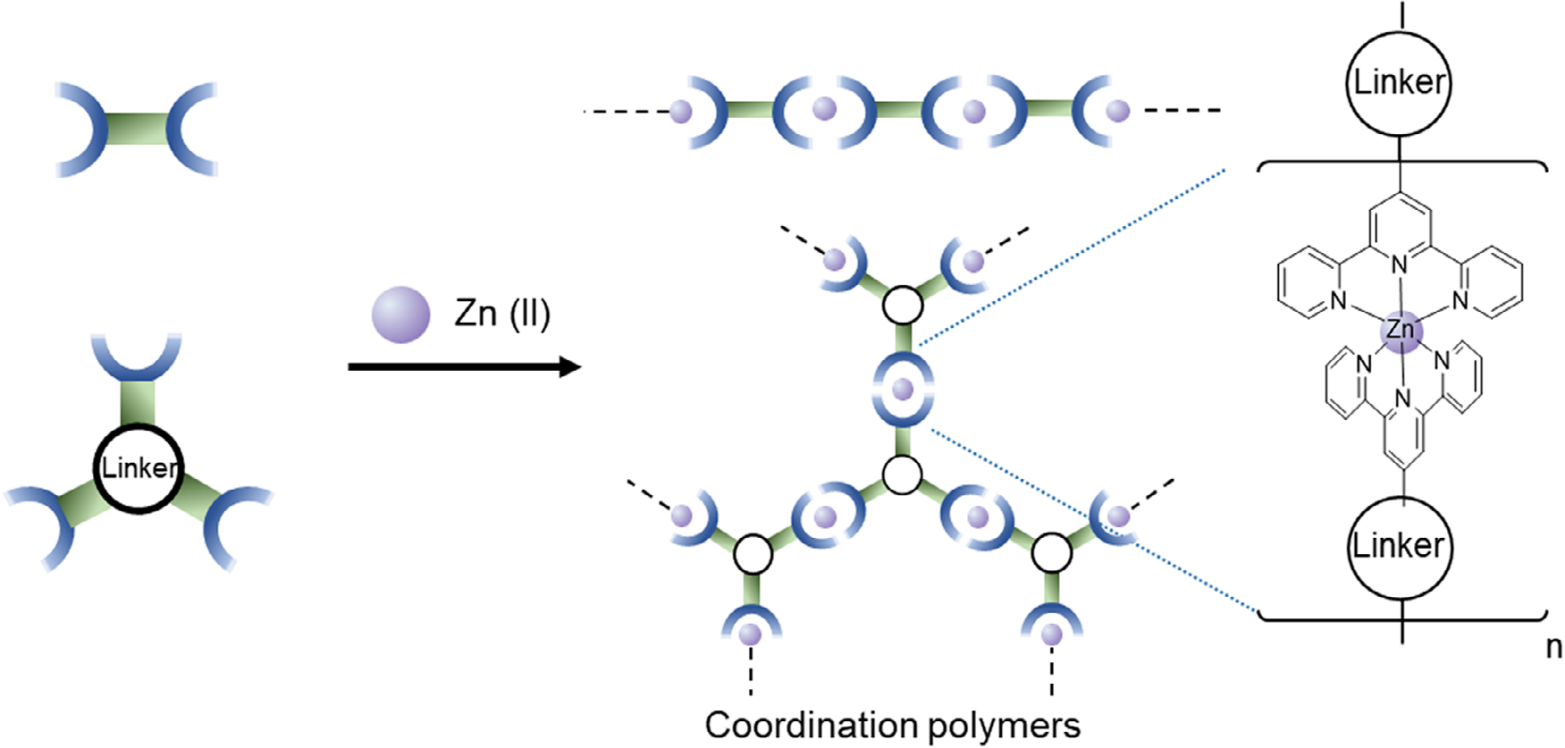
Schematic diagram of the synthesis of coordination polymers via bis–Tpy ligands and Zn(II).

When Tpy ligands are functionalized with other substituents bearing additional coordination sites, the resulting Tpy–Zn motifs are able to propagate through metal–ligand linkages into extended repeatable architectures. Depending on the ligand design, these assemblies can be 1D chains or 2D/3D networks, providing structural diversity beyond small complexes. Unlike crystalline analogues, such amorphous coordination polymers lack long–range periodicity yet can still form mechanically robust architectures through random coordination junctions and entangled chains [92, 93]. As illustrated in Figure 17, the head–to–tail connection of Tpy–Zn motifs propagates in multiple directions, yielding irregular but interconnected networks. Compared with the reversible and stimuli–responsive matrix of supramolecular gels, these amorphous coordination polymers exhibit greater structural stability and durability, representing a transition from dynamic assemblies to permanent robust coordination networks. Furthermore, highly ordered MOFs can also be obtained by rational modulation of ligand flexibility, porosity, and functionality through careful design of the ligand structure and coordination environment. This approach offers a versatile tool for designing functional materials without demanding stringent crystallization conditions.

**FIGURE 18:**
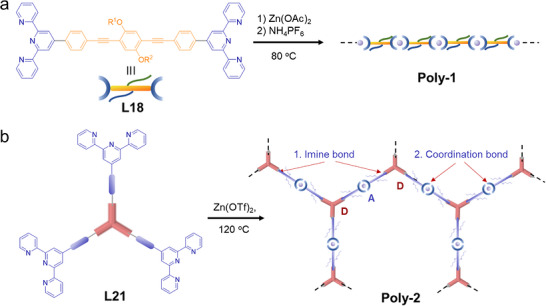
(a) Construction of linear polymer **Poly–1** by coordinating Tpy ligand **L18** with Zn(OAc)_2_ [80]. (b) Formation of network polymer **Poly–2** by incorporating TPAP, aldehyde–functionalized ligand **L21** and Zn(OTf)_2_ through Schiff–base condensation and metal coordination [81].

Due to the photophysical stability, Lewis acidity, and coordination flexibility of Zn(II), Tpy–Zn–based coordination polymers have garnered significant research interest in recent years [92]. Schubert and colleagues reported a series of structurally tunable coordination polymers [94]. As shown in Figure 18a, the bis–Tpy ligand **L18** with poly–ε–caprolactone side chains readily generated rigid π–conjugated polymer **Poly–1**. The alkyl side chains conferred enhanced solubility in organic solvents, overcoming a critical limitation of poor processability by integrating solubilizing units into the ligand framework [80]. This modular design enables device–compatible fabrication while preserving photophysical properties of the pincer precursors. Future refinements to optimize emission profiles and charge transport characteristics allow their optoelectronic applications. Furthermore, **Poly–2** could be constructed from an aldehyde–functionalized Tpy ligand **L21**, triphenylamine–based linker (TPAP), with Zn(II) ions (Figure 18b) [81]. The introduction of TPAP not only provided a donor unit but also altered the coordination orientation of the Tpy–Zn nodes, thereby directing the assembly into a 2D network after further condensation with amino building blocks. These studies demonstrate the structural diversity and functional tunability of Tpy–Zn based coordination polymers. By incorporating solubilizing side chains or donor–acceptor motifs into the Tpy ligand framework, the resulting materials exhibit improved solution processability, enhanced light–harvesting capabilities, and tailored charge transport behavior. These advantages are essential for further application of Tpy–Zn polymers toward optoelectronic and photocatalytic devices.

Despite the significant progress in Tpy–Zn–based amorphous materials, substantial challenges remain in their rational design, structural regulation, and comprehensive characterization. Among these challenges, the precise characterization of amorphous structures stands as a major bottleneck – as their inherent lack of long–range order makes conventional single–crystal X–ray diffraction (SCXRD) analysis ineffective. This limitation impedes the accurate determination of coordination geometries, connection patterns within the amorphous matrix, and, ultimately, the structure–property relationships, which are crucial for material design and performance optimization. To tackle this critical issue, a combination of advanced characterization techniques and theoretical calculations has been employed. For example, extended X–ray absorption fine structure (EXAFS) spectroscopy allows for the unambiguous probing of the local coordination environment around Zn(II) centers, including bond lengths, coordination numbers, and local symmetry, thereby directly compensating for the inability of SCXRD to reveal short–range structural features. Meanwhile, solid–state NMR spectroscopy offers valuable insights into ligand connection, molecular dynamics, and structural heterogeneities within the matrix. In addition to these experimental techniques, density functional theory (DFT) calculations play an indispensable role, facilitating the modeling of plausible structural configurations, the evaluation of electronic structures and charge–transfer pathways, and the establishment of robust structure–property correlations. Future research should prioritize two synergistic pathways: (i) designing conformationally diverse Tpy ligands to construct higher–dimensional networks (3D hierarchical architectures), and (ii) designing ligands with programmable geometries to create structure and cavity defined coordination polymers. These approaches would yield materials suitable for selective molecular recognition and diffusion–controlled processes, ultimately advancing the development of multifunctional soft materials with stimuli–responsive and predictable functionalities.

#### 3.2.2 MOFs

**FIGURE 19:**
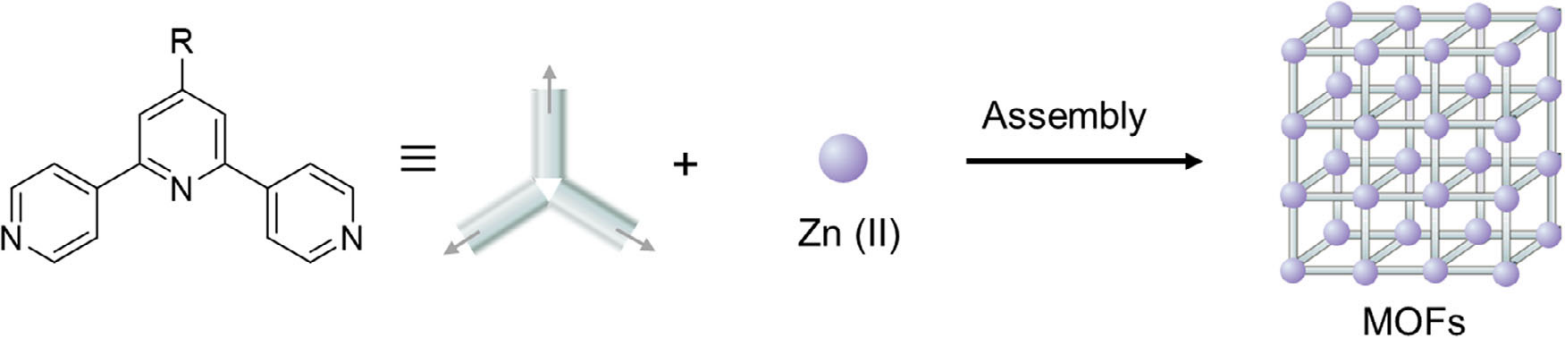
Schematic diagram of the preparation of MOFs from Tpy–Zn complexes.

MOFs, crystalline porous materials designed through coordination–driven self–assembly between metal ions and organic ligands, are also accessible by Tpy–Zn subunits (Figure 19) [95, 96, 97, 98]. Compared with amorphous analogues, MOFs exhibit a higher crystallinity, enabling precise control over porous geometry and connectivity. Their high surface area, tunable pore size, and structural versatility enable broad applications in gas adsorption and separation [6, 99, 100], catalysis [101, 102, 103, 104], bioimaging [105, 106], drug delivery [107, 108], and so on. Tpy ligands emerge as a powerful candidate for MOFs synthesis, transcending the limitations of conventional approaches, and the structural rigidity, predictable coordination, and facile functionalization unlock a new approach in engineering porous crystalline materials. Within this context, Zn(II)–based systems have attracted particular interest: the d^10^ configuration of Zn(II) favors the formation of robust frameworks while imparting desirable photophysical properties and good biocompatibility. Remarkably, the rigid tridentate chelating ability of the Tpy ligand provides a robust and well–defined geometry upon coordination with Zn(II) ions, which significantly enhances the thermodynamic stability of the resulting frameworks and ensures structural integrity under external disturbances. Furthermore, the relatively dynamic and/or hemilabile nature of Zn–N coordination bonds facilitate additional structural adaptability, enabling reversible self–adjustment/healing and stimulus–responsive behaviors without significant framework collapse. In this context, Tpy–Zn motifs are suitable for serving as secondary building units (SBUs), exhibiting a delicate balance between stability and functionality, which is crucial for accessing robust MOFs with diverse applications in sensing, catalysis, and optoelectronic devices [91, 109, 110].

**FIGURE 20:**
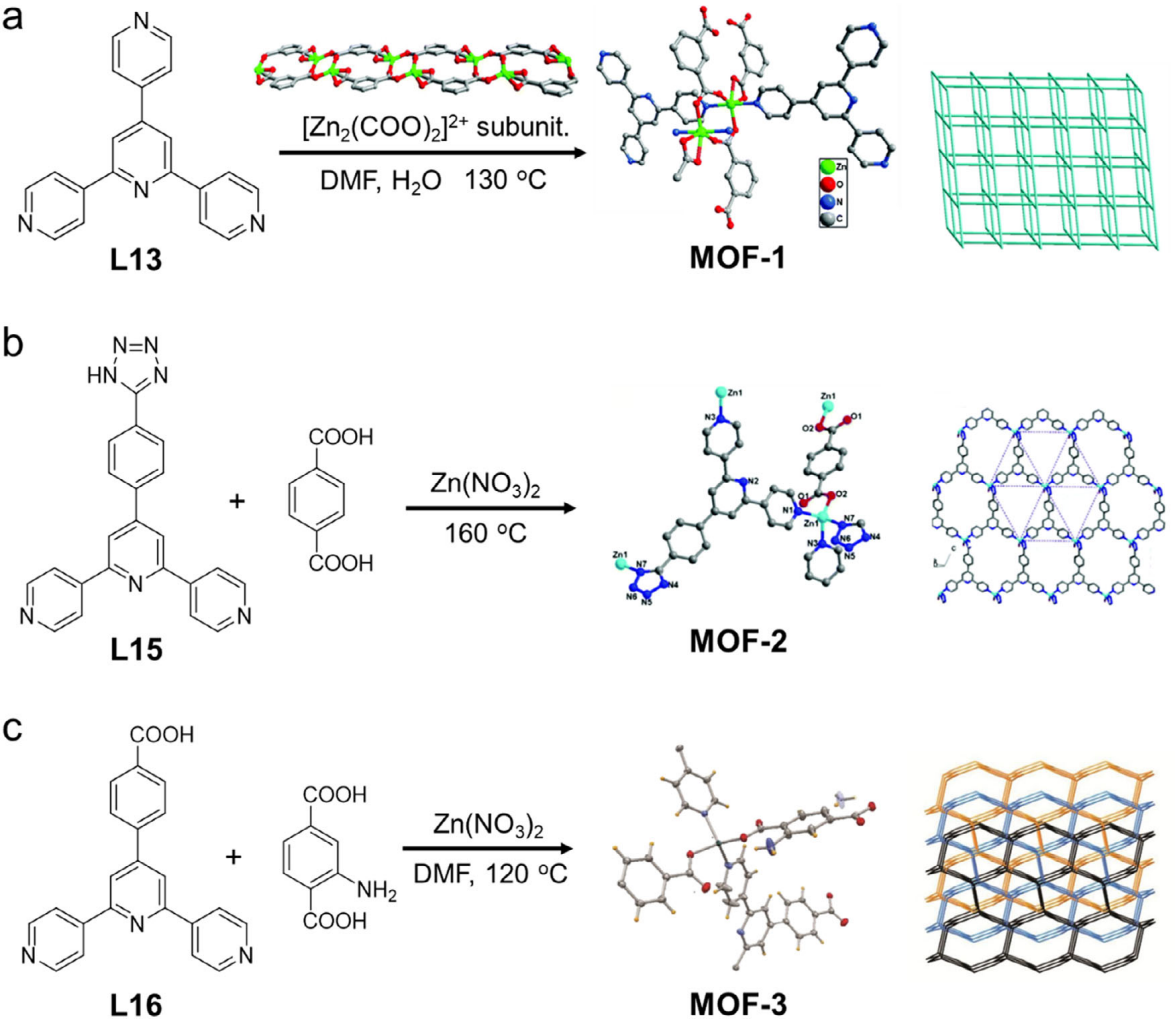
(a) Coordination of Tpy–ligand **L13** and Zn(II) with carboxylate linkers [Zn_2_(COO)_2_]^2+^ subunit) to form highly porous 3D crystalline **MOF–1**. Reproduced with permission [71]. Copyright 2019, Royal Society of Chemistry. (b) Assembly of ligand **L15** with Zn(II) through multidentate coordination to generate a triple–interpenetrated structure **MOF–2**, with ordered triangular channels and high structural stability. Reproduced with permission [79]. Copyright 2019, Royal Society of Chemistry. (c) Construction of 2D layered material **MOF–3** from ligand **L16** and Zn(II). Reproduced with permission [72]. Copyright 2025, Royal Society of Chemistry.

Harnessing the coordination versatility of Tpy ligands and the structural tunability of MOFs, a series of Tpy–Zn based MOFs have been constructed with diverse architectures and functionalities. In 2018, a rigid ditopic Tpy–ligand **L13** was coordinated with the dinuclear [Zn_2_(COO)_2_]^2+^ subunit to afford a 2D lamellar framework **MOF–1** (Figure 20a) [71], in which each Zn(II) center adopted a distorted octahedral coordination geometry with Zn–N distances of approximately 2.125–2.157 Å and Zn–O distances of 2.042–2.344 Å. The framework featured dynamic Zn···Zn interactions and extensive π–π stacking between adjacent Tpy moieties, endowing it with reversible single–crystal–to–single–crystal transformations upon guest exchange. Such structural adaptability, arising from the flexible Zn···Zn contacts and rigid Tpy backbone, highlights the advantage of Zn–based nodes in constructing responsive crystalline matrices.

In contrast to the previous system featuring a monodentate pyridyl group, the triazole moiety with its distinct coordination environment has become a focus in current research. In 2019, a triazole–functionalized Tpy–ligand **L15** was used to construct **MOF–2** (Figure 20b), in which Zn(II) centers adopt distorted tetrahedral coordination geometries [79]. Each Zn(II) is coordinated by three nitrogen atoms from terpyridine and triazole moieties, along with one oxygen atom from a carboxylate group. The appended triazole units not only expand the coordination environment but also act as flexible linkers, introducing additional rotational freedom into the framework. This structural adaptability enables reversible framework distortion under external stimuli, accompanied by luminescence switching behavior. The incorporation of triazole groups thus serves a dual role: enhancing connectivity for stable framework formation while introducing adaptive flexibility, making **MOF–2** a promising candidate for molecular sensing and stimulus–responsive materials.

Building on the observations from **MOF–1** and **MOF–2**, Tpy–ligand **L16** was deliberately designed to pre–organize the coordination geometry and enable multidirectional bridging of Zn(II) nodes (Figure 20c) [72]. In this design, three rigid Tpy arms radiate from a central core with defined angles, creating a spatially symmetric yet highly directional coordination geometry. This arrangement not only increases the number of coordination vectors available to the Zn(II) centers but also minimizes steric clashes between ligands, thereby promoting extended framework growth in three dimensions. The resulting **MOF–3** exhibits a high porosity of 40.6% and a remarkably stable lattice, maintaining its crystallinity under both thermal and chemical stress. Such combination of tripodal preorganization, high connectivity, and controlled interpenetration illustrates a significant advancement in ligand–driven framework engineering, demonstrating how careful Tpy ligand design can steer the assembly of Zn–based MOFs toward complex yet ordered robust materials.

Compared with other metals, Zn(II) possesses several intrinsic merits for MOFs construction. Its closed–shell d^10^ configuration avoids electronic quenching and affords predictable coordination chemistry; its geometric flexibility enables diverse framework topologies; and its redox–inert nature ensures stability under light and chemical stress. These features render Zn(II) not only a versatile connector but also a determinant of framework dimensionality and robustness. Remarkably, Tpy–Zn–based MOFs may also be considered as promising candidates for photocatalysis, which will be further elaborated in the subsequent sections.

### 3.3 Supramolecular Cages

**FIGURE 21:**
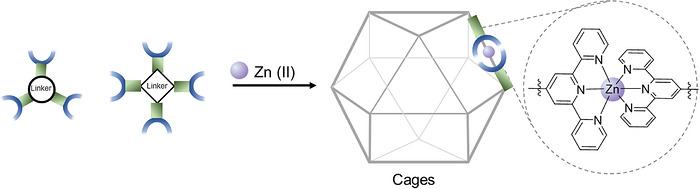
Schematic diagram of the coordination and assembly process for constructing metal–organic supramolecular cages based on Tpy ligands and Zn(II) salts.

Besides gels, polymers, and MOFs, supramolecular cages can also be fabricated from Tpy–Zn complexes. Through rational molecular design, ligands incorporating multiple Tpy ligands can be connected with Zn(II) ions to form supramolecular cages with diverse structures (Figure 21) [31, 111, 112]. Such design allows the transition from extended frameworks to cages, in which the overall geometry, cavity size, and stability are precisely controlled by the ligand preorganization and the dynamic yet reversible Tpy–Zn coordination. This structural tunability has positioned Tpy–Zn motifs as versatile building blocks for constructing sophisticated supramolecular systems, laying the foundation for further applications in host–guest recognition [113, 114], full–color emissive materials [115, 116] and optoelectronic devices [117].

**FIGURE 22:**
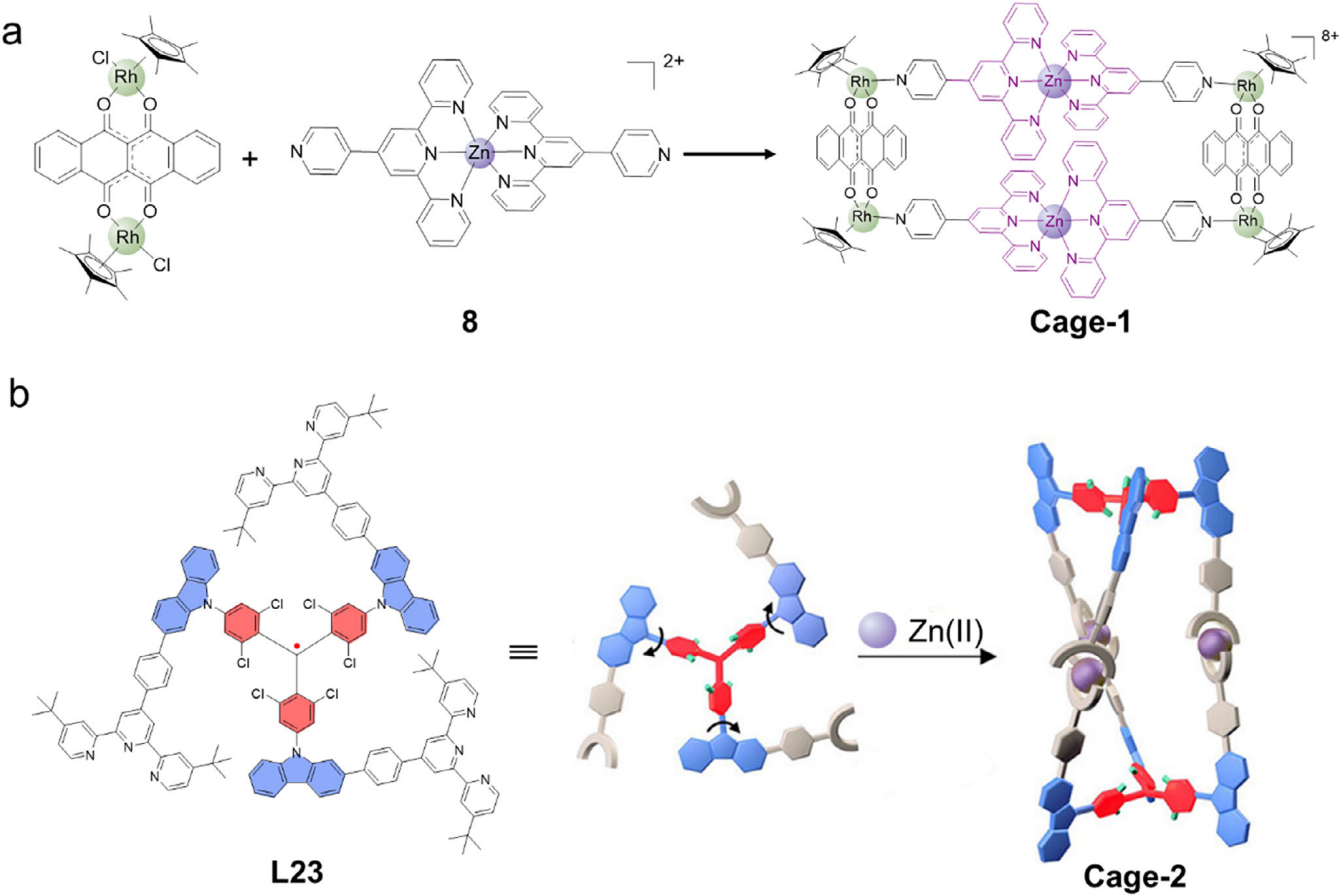
(a) Construction of **Cage–1** from bis–Tpy complex **8** and transition metal nodes [75]. (b) Construction of **Cage–2** from Tpy–ligand **L23** and Zn(II) salt. Reproduced with permission [82]. Copyright 2023, American Chemical Society.

In recent years, using these kinetically or thermodynamically stable Tpy–Zn units to construct novel and complicated supramolecular structures with different and diverse topology has attracted considerable attention [33]. In 2014, Jin and co–workers designed and synthesized the Tpy–metal complex **8** in the form of [Tpy–Zn–Tpy]^2+^ and the new [4+2] six–core metal–organic topological molecule **Cage–1** (Figure 22a) with a semi–belted structure [(Cp*_2_Rh_2_(µ–DHNA)Cl_2_] (DHNA represents 6,11–dihydroxy–5,12–naphthalenanthrone) [75]. In this system, metal nodes such as Zn and Rh can be replaced by other metal centers, including Cu or Ir, leading to the formation of structurally diverse supramolecular cages.

By further modifying Tpy ligands with functional moieties like radical or emissive groups, supramolecular cages can be endowed with rich photophysical and electronic properties. In 2023, Wang and co–workers reported a supramolecular **Cage–2** assembled from the tridentate Tpy–ligand **L23** bearing a central radical core (Figure 22b) [82]. Upon coordination with Zn(NO_3_)_2_∙6H_2_O, **L23** underwent a coordination–driven self–assembly process to afford a twisted trigonal–prism **Cage–2** composed of two ligands and three Zn(II) nodes. The integration of the radical core within the rigid Tpy–Zn framework not only reinforces the structural stability of the prism cage but also endows it with unique photophysical and spin–related properties, demonstrating the versatility of Tpy–Zn coordination chemistry in constructing multifunctional radical–based supramolecular architectures.

**FIGURE 23:**
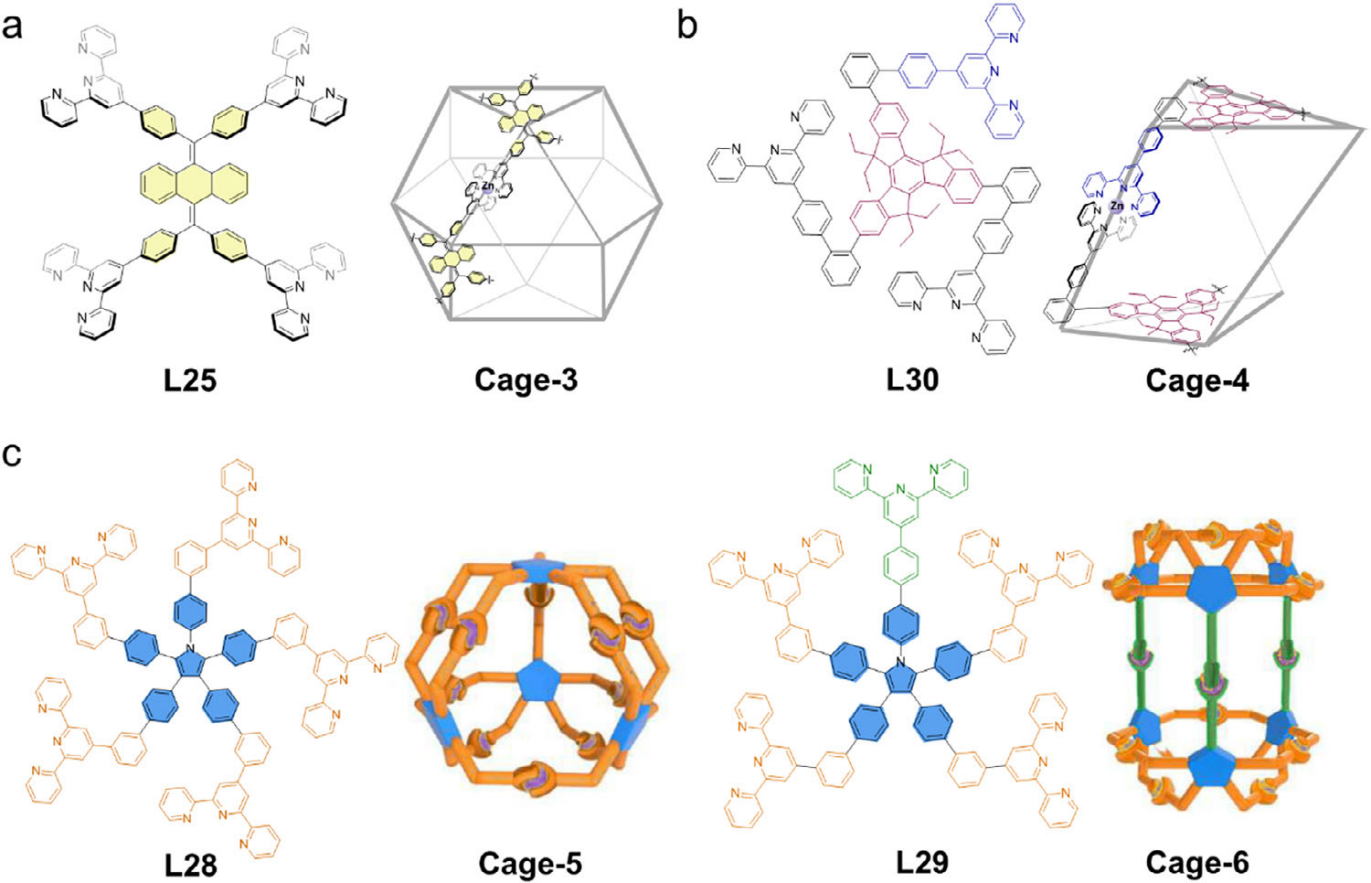
(a) Construction of **Cage–3** by Tpy–ligand **L25** and Zn(II) salt [62]. (b) Construction of **Cage–4** by Tpy–ligand **L28** and Zn(II) salt [63]. (c) Construction of **Cage–5** and **Cage–6** using Tpy–ligands **L28** and **L29** and Zn(II) salts, respectively. Reproduced with permission [83]. Copyright 2025, Wiley.

Besides radical units, introducing emissive chromophores into Tpy–ligands can further enhance the photoluminescent properties of the resulting supramolecular cages. In 2022, Wang and co–workers reported a large **Cage–3** constructed by chelating of *tetra*–Tpy–ligand **L25,** bearing a dihydroanthracene core with Zn(NSO_2_CF_3_)_2_ (Figure 23a) [62]. The resulting structure featured a well–defined octahedral topology with a molecular size of approximately 2.8 nm. Multiscale imaging techniques confirmed its uniform geometry and dispersity. The incorporation of the dihydroanthracene chromophore not only contributes intrinsic luminescence but also illustrates how rational ligand design can integrate photophysical functionality into high–symmetry, nanoscale Tpy–Zn supramolecular cages.

Subsequently, the same group further developed a helical supramolecular **Cage–4** by coordinating Tpy ligand **L30** with Zn(NO_3_)_2_∙6H_2_O in 2024 (Figure 23b) [63]. The ligand featured three Tpy arms arranged to favor directional rotation, and self–assembly yielded a twisted octahedral cage with well–defined *P/M* helicity. Single–crystal X–ray analysis confirmed its topological chirality, with a height of 18.9 Å and Zn(II) centers binding each Tpy arm in a helically oriented manner. This work illustrates a strategy for encoding chirality into Tpy–Zn supramolecular architectures through precise ligand preorganization. It provides a foundation for constructing functional chiral materials, potentially enabling developments in asymmetric recognition, circularly polarized luminescence, or enantioselective catalysis.

More recently, Li and co–workers reported two novel Tpy–Zn cages (**Cage–5** and **Cage–6**) assembled from pyrrole–based Tpy–ligands **L28** and **L29**, respectively (Figure 23c) [83]. Both ligands contain a central pyrrole core connected to five Tpy arms that coordinate with Zn(II) to form discrete cages. A minor structural change between the two ligands, namely the connection position of one Tpy unit in **L29** compared with **L28**, results in distinctly different cage geometries. The symmetric ligand **L28** gives a compact cage composed of intertwined parallelogram and triangular faces, while the slightly asymmetric **L29** leads to a more elongated cage with a hexagonal arrangement of coordination nodes. The resulting cages possess intrinsic D–A architectures, which create well–separated redox–active sites upon light irradiation. This structural feature enables efficient exciton dissociation and directional electron transfer, endowing the cages with significant potential for photocatalytic applications.

Over the past decade, researches on Tpy–Zn supramolecular cages have quickly been developed from simple 2D assemblies to sophisticated 3D polyhedral after rational design and modulation of ligand topology. The representative examples (**Cage–1** to **Cage–6**) highlight from controlling cavity size with ditopic ligands to introduce electron–rich substituents and finally to realize tripodal or multibranched motifs that benefit well–defined polyhedral structure formation. These works not only demonstrate the structural adaptability of the Tpy–Zn coordination motif, but also validate its potential in achieving emergent functions such as selective molecular encapsulation and tunable luminescence. Nevertheless, despite these impressive advances, challenges remain in streamlining multistep ligand synthesis, improving assembly efficiency, and achieving robust stability in aqueous or biologically relevant media. Addressing these issues through modular ligand design and solvent–compatible assembly strategies will be essential for unlocking and extending the feasibility of Tpy–Zn cages toward sensing, drug delivery, and photonic devices.

### 3.4 Composites

**FIGURE 24:**
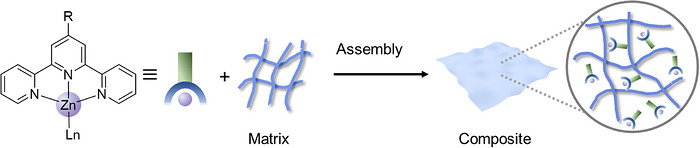
Schematic diagram of the fabrication of composite with Tpy–Zn based complexes.

In addition to serving as versatile building blocks for supramolecular gels, polymers, MOFs, and cages, Tpy–Zn complexes can also be fabricated into various composites via doping or co–assembling with external matrix (Figure 24) [118, 119, 120, 121]. This strategy utilizes the tunable nature of peripheral substituents (R groups) and counterions (L), which can be engineered to interact with polymer backbones, biopolymers, and/or inorganic substrates. Such hybridization not only imparts improved mechanical properties, flexibility, and processability of the resulting composites, but also enriches their photophysical and chemical behaviors. Remarkably, doping Zn–based chromophores into rigid matrix may convert their intrinsic fluorescence into persistent RTP [64], while simultaneously allowing multifunction like cuttability, recyclability, and environmental adaptability. Thus, the doping approach represents a powerful strategy to convert the structural and luminescent merits of Tpy–Zn complexes into practical and multifunctional composites.

**FIGURE 25:**
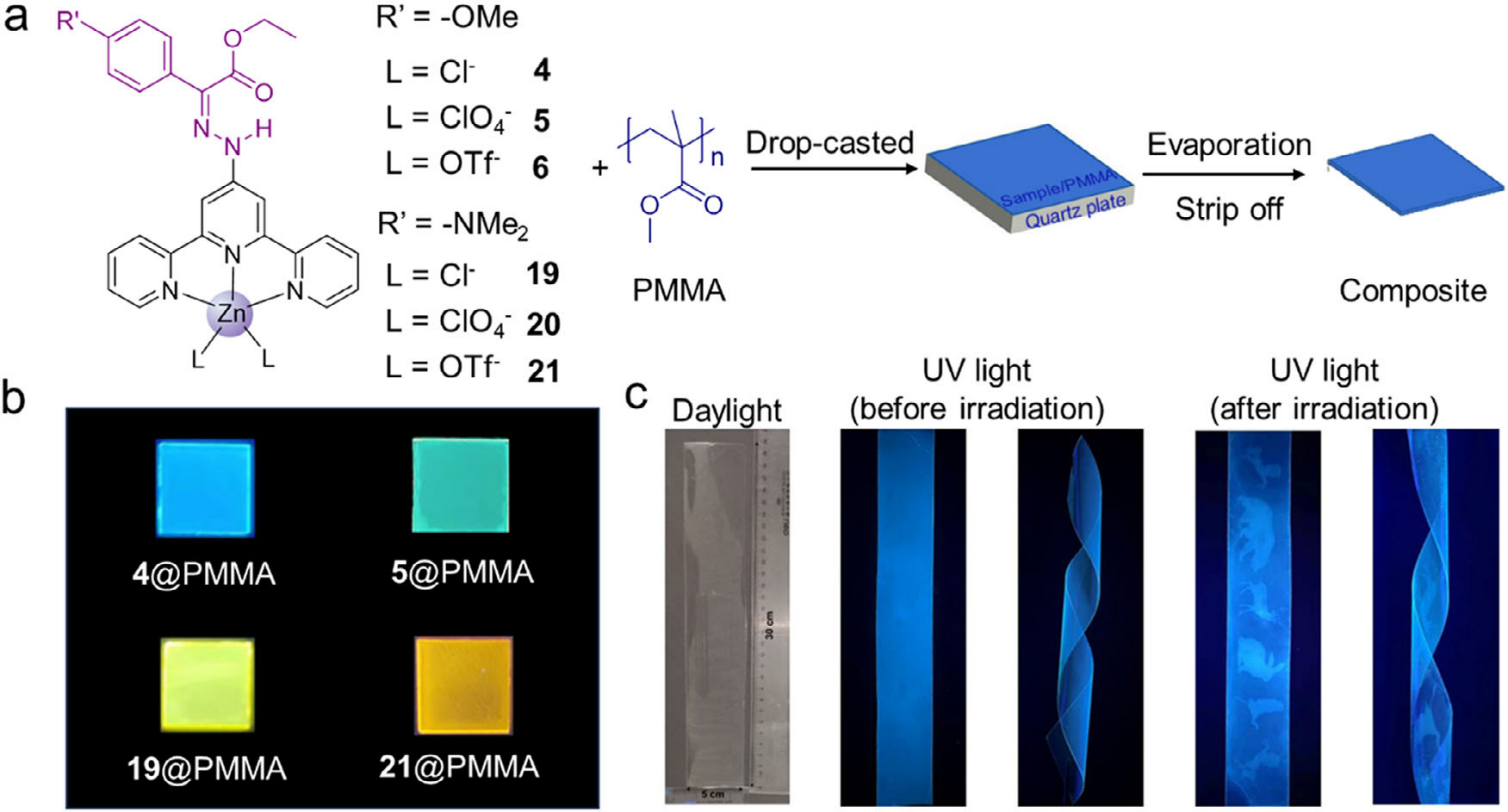
(a) Schematic illustration of composite fabrication strategies for complexes **4–6** and **19–21** with a PMMA matrix. (b) Photographs of film composites under 365 nm UV light irradiation. (c) Flexible luminescent tag (5×30 cm) formed by drop–coating complex and PMMA onto a polyethylene terephthalate (PET) substrate before and after UV irradiation. Reproduced with permission [65]. Copyright 2022, Wiley.

In 2022, Zhao and co–workers developed a doping strategy by embedding a series of photo–responsive Tpy–Zn complexes **4–6** and **19–21** into polymethyl methacrylate (PMMA) matrix (Figure 25a) [65]. Transparent thin films were fabricated by drop–coating mixtures of Tpy–Zn complexes (2 wt%) with PMMA (98 wt%) on quartz plates, resulting in uniform luminescent films with emission colors spanning blue, cyan, green, yellow, and orange depending on the ligand design and counterions (Figure 25b). Importantly, these films maintained the light–activated isomerization capability of the molecular precursors, allowing reversible luminescence switching within the polymeric environment. Besides tunable optical outputs, the resulting films exhibited high flexibility and mechanical processability, and their brightness could be modulated under light irradiation, demonstrating potential as flexible substrates for light–printing applications (Figure 25c). Overall, this dopping strategy illustrates how embedding small–molecule Tpy–Zn complexes into polymer matrices can effectively translate molecular–level functions into processable materials, thereby extending the scope of their practical applications.

**FIGURE 26:**
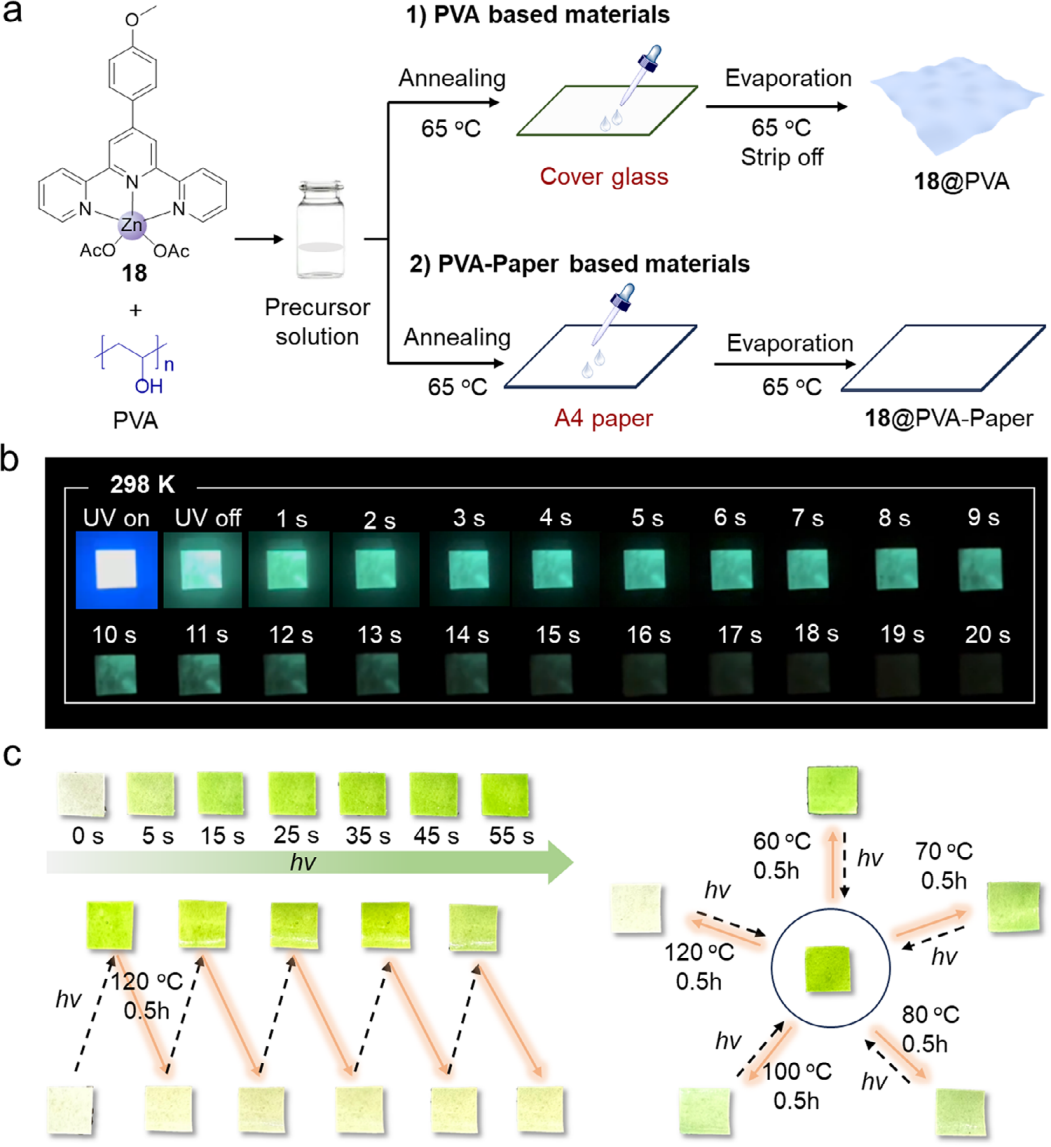
(a) Schematic illustration of two strategies for fabricatinig composite from complex **18** and PVA: 1) direct casting onto a cover glass to form transparent PVA films (**18**@PVA), and 2) deposition onto A4 paper to produce flexible PVA–Paper–based composites (**18**@PVA–Paper). (b) Photographs of the room–temperature phosphorescence (RTP) exhibited by composite **18**@PVA after UV light irradiation (λ_ex_ = 365 nm) at 298 K. (c) Photochromic behaviors, including response time, reversibility, and thermal stability, of the composite **18**@PVA–Paper. Reproduced with permission [64]. Copyright 2024, Wiley.

In 2024, Tu and co–workers reported a water–soluble D–π–A type Tpy–Zn complex **18** bearing methoxy (–MeO) substituents and acetate (OAc^−^) counterions, where the –MeO groups acted both as electron donors and H–bond acceptors, facilitating hybridization with poly(vinyl alcohol) (PVA) matrices (Figure 26a) [64]. Two doping strategies were adopted: casting the precursor solution on glass produced free–standing PVA films (**18**@PVA), or deposition on A4 paper directly yielded flexible hybrid composite (**18**@PVA–Paper). The former exhibited stable RTP, enabled by π–π stacking and hydrogen–bonding interactions between complex **18** and the polymer host, which suppressed non–radiative relaxation pathways (Figure 26b). The latter showed rapid and reversible photochromism under UV irradiation, with distinct green color appearing within seconds after irradiation and complete bleaching upon mild heating (Figure 26c). Mechanistic studies attributed this photochromism to the formation of radical species stabilized by the polymer matrix. Remarkably, this work demonstrates that after rational doping, structurally simple Tpy–Zn complexes are readily to form multifunctional hybrid materials with diverse photophysical behaviors. By incorporating RTP and dynamic chromism into polymer and paper supports, authors not only extended the feasibility of Tpy–Zn complexes but also provide a general strategy for developing next–generation optoelectronic and responsive materials.

These studies demonstrate how to embed or dope Tpy–Zn complexes into different polymeric supports and provide additional functions. Unlike PMMA simply acts as a rigid and transparent host to preserve photoisomerization and enables flexible luminescent film formation, while PVA serves as a hydrogen–bonding and water–compatible matrix that unlocks the free rotation of Tpy–Zn complex and exhibits entirely new properties such as RTP and reversible photochromism. Such comparisons highlight that doping strategies not only can fabricate diverse composites from simple small–molecule complexes but also give rise to novel functionalities, thereby opening new pathways for developing multifunctional organometallic composites.

## 4 Applications of Tpy–Zn Complexes and Corresponding Functional Materials

The preceding section outlined the construction of Tpy–Zn–based materials, ranging from coordination polymers and supramolecular gels to MOFs, cages, and composites, revealing how coordination modes, ligand functionalities, intermolecular interactions, and matrix effects shape their diverse architectures. The resulting complexes and materials not only govern the intrinsic physical and chemical characteristics of Tpy–Zn molecules but also exhibit various stimuli–responsive behaviors and new functionalities. In particular, the structure–property correlation may provide a rational hint for classification of their potentials into three domains: (1) Dynamic binding properties of Tpy–Zn complexes toward various guest molecules enable selective molecular visualization and/or recognition. (2) The low toxicity and tunable planar structures of Tpy–Zn complexes, which are easily assembly under various scenarios allow to access versatile luminescent and full–color emission materials with potential applications toward bioimaging, information encryption, anti–counterfeiting and even optoelectronic devices. (3) The robust but flexible coordination structure and accessible metal sites with ligand–exchange ability create opportunities for catalytic transformations. Concurrently, we highlight emerging trends and future directions propelling this rapidly evolving research field.

### 4.1 Visual Molecular Recognition

**FIGURE 27:**
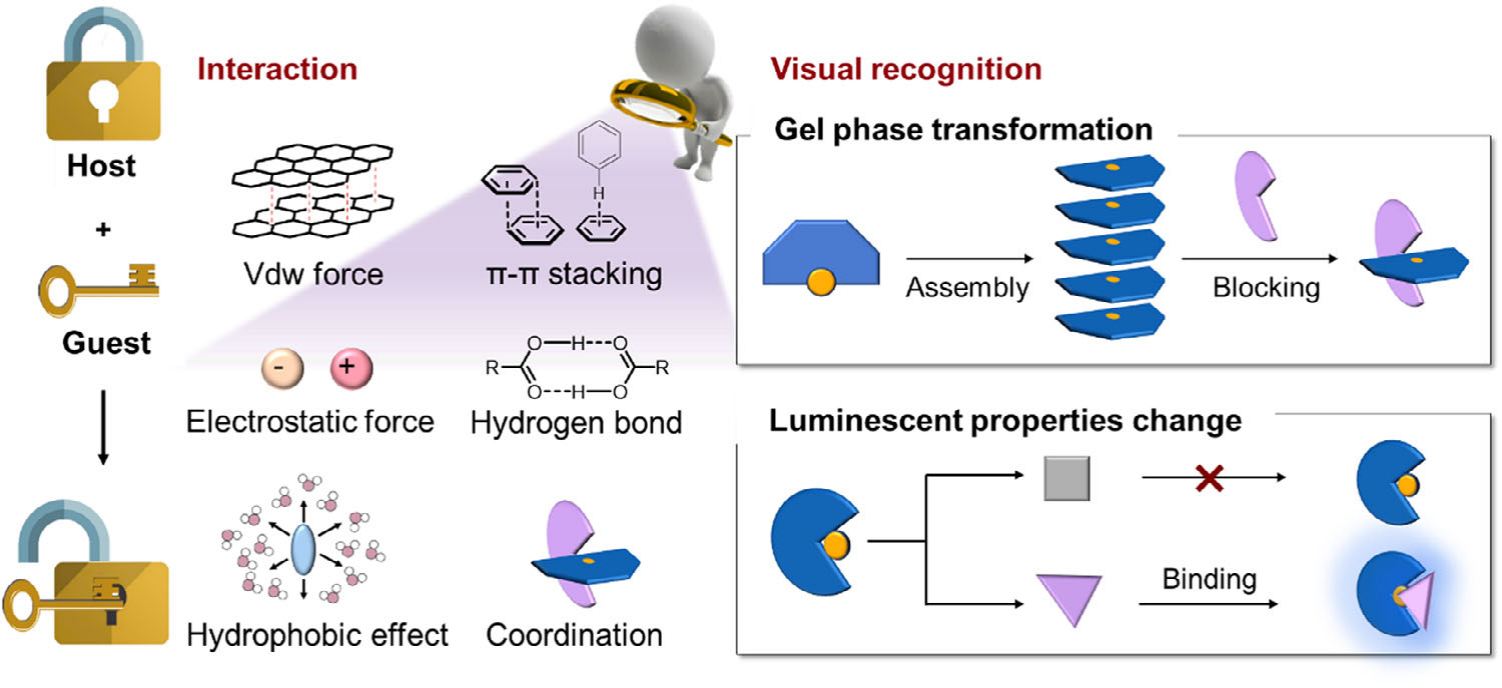
Schematic diagram of the molecular recognition process with colorimetric or phase–alteration strategies.

Molecular recognition, a central theme of supramolecular chemistry, relies on the selective interaction between host and guest molecules via weak noncovalent forces including hydrogen bonding, π–π stacking, van der Waals force and electrostatic interactions (Figure 27) [122, 123, 124]. Conventionally, the discrimination of these interactions has heavily depended on sophisticated analytical instruments, which, although highly accurate, are often time–consuming, costly, and require specialized training. In contrast, visual molecular recognition offers a straightforward, low–cost, and user–friendly choice, where molecular interaction or response could be amplified, and even recognized by the naked eyes [125]. This approach enables real–time detection under ambient conditions, eliminating the requirements of tedious preprocess of samples, expensive equipment and facilities and opening new approach for practical sensing in diverse scenarios.

Tpy–Zn complexes and/or corresponding matters are considered as particularly suitable candidates for molecular recognition due to labile Zn–N coordination bond, easily substituted counter ions, and electronic and steric tunable property of Tpy ligands. Remarkably, the electronic and steric substitution allows the resulting pincer complexes to selective bind guest molecules or accelerate dynamic supramolecular assembly, even leading to obvious colorimetric or phase–alteration and amplifying recognition signals in front of naked eyes. Generally, two complementary strategies have been developed (Figure 27): (1) Phase transformation, in which selective ligand exchange or guest molecular substitution may perturb/benefit the possible Tpy–Zn coordination assembly process, like selective gel collapsing or formation, representing a practical approach for molecular recognition. This strategy incorporating the dynamic assembly nature of planar Tpy–Zn complexes with selective coordination ability has been successfully exploited in the selective recognition of various amino acids, nucleotides and other biological active molecules. (2) Optical signal variation (colorimetric/fluorescent/phosphorescent), where guest–induced alterations in coordination patterns or electronic distribution of Tpy–Zn complexes directly affect their colorimetric, fluorescence or phosphorescence behaviors, leading to changes in emission color, intensity, or lifetime. This approach amplifies diverse subtle molecular interactions as distinct optical outputs, and shows broad potential in visual sensing, information encryption, and anti–counterfeiting.

#### 4.1.1 Phase Transformation

The phase transformation approach facilitates visual molecular recognition by modulating guest–induced changes in weak supramolecular interactions that result in the formation or disruption of soft matter, such as gels. Interacting with specific analytes may induce phase changes, like gel collapsing or formation, which is easily observed by the naked eyes [57, 126, 127]. This approach is particularly suitable for rapid, on site and selective detection, providing a convenient and practical instrument–free discrimination protocol in various scenario and extensively employing in recognition of various ions, molecular isomers and analogues, as well as a number of biologic active species [56, 61, 78]. In this section, we primarily focus on selective phase transformations of Tpy–Zn complexes induced by diverse guest molecules.

**FIGURE 28:**
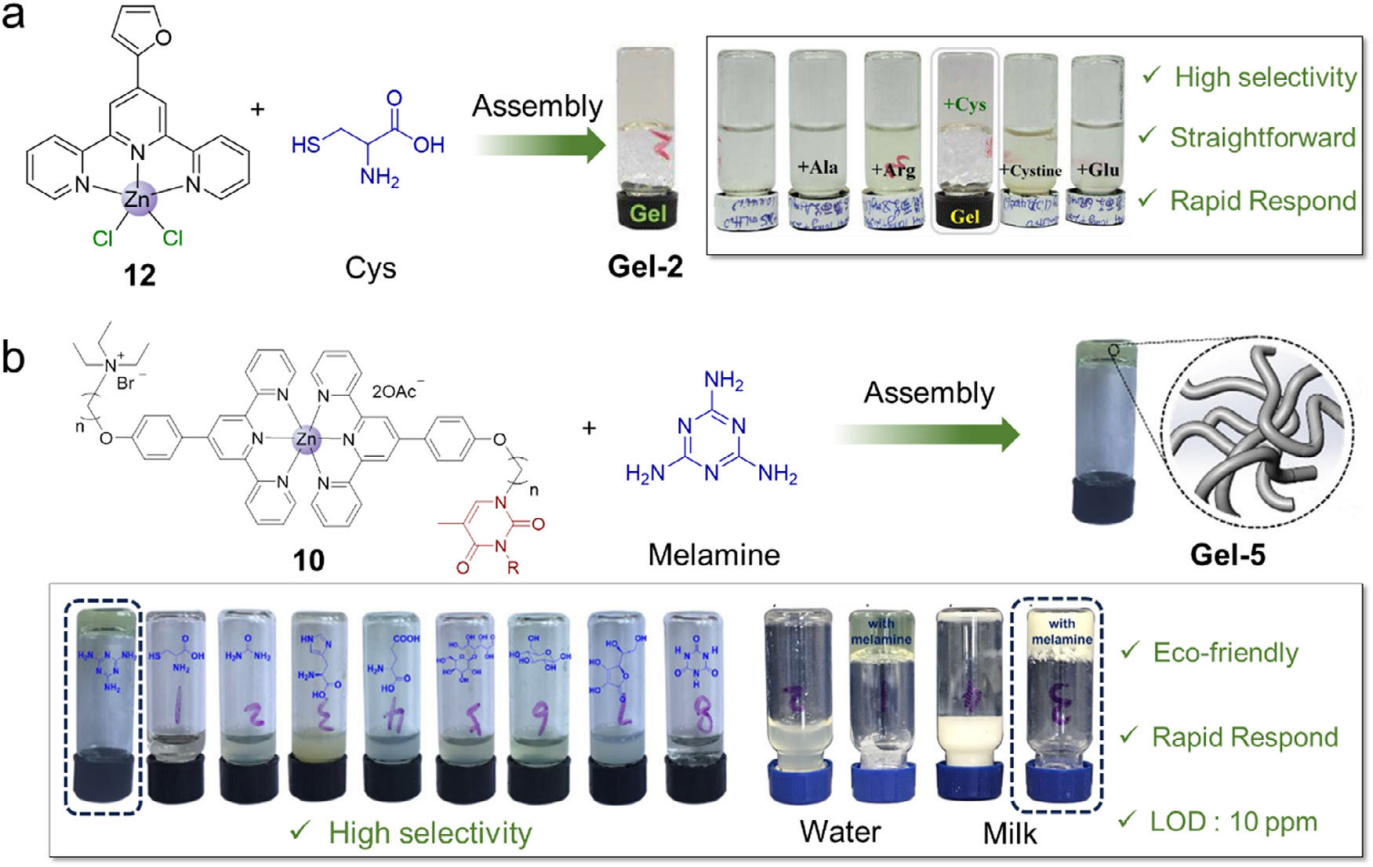
(a) Visual recognition of cysteine (Cys) based on a gel–to–sol transition induced by selective substitution of Cl^−^ of complex **12** by Cys. Reproduced with permission [56]. Copyright 2014, Royal Society of Chemistry. (b) Visual recognition of melamine through selective gel formation by hydrogen bonding interaction with Tpy–Zn complex **10,** and their application in detection of trace melamine in milk samples. Reproduced with permission [67]. Copyright 2019, Elsevier.

Sulfur–containing amino acids, including cysteine (Cys), homocysteine, and glutathione, play essential roles in biological systems. However, their direct visual detection remains challenging due to structural similarities and similar solubility properties. In 2014, Tu and co–workers addressed this challenge by developing Tpy–Zn complex **12**, which enabled naked–eye recognition of Cys via a selective hydrogel formation (Figure 28a) [56]. Addition of 2 equiv. of Cys to an aqueous solution of complex **12** induced immediate formation of a transparent hydrogel (**Gel–2**), while other amino acids failed to produce gel under the otherwise identical test conditions. This selectivity may arise from strong coordination between the thiol group of Cys and Zn(II) center of the pincer complex. After coordination, the resulting complex exhibits enhanced water solubility and readily assembles to form a robust hydrogel. This work represents a pioneering example of visualized sensing of biologically active molecules by selectively supramolecular gel formation.

Subsequently, the same group reported two–component supramolecular **Gel–5**, readily formed by the selective assembly of complex **10** with melamine, achieving visual detection of melamine via selective gel formation even down to 10 ppm (Figure 28b) [67]. Driven by multiple hydrogen bonding between the thymine units attached to pincer ligand and melamine, along with π–π stacking, the adduct formed by melamine and complex **10** readily self–assembles into hydrogel. Crucially, this discrimination process remains effectively even with the real milk samples. Obvious gel formation was observed with melamine–containing milk, especially, no additional pretreatment is required. This study offers a rapid, straightforward, cost–effective, and practical alternative protocol to conventional instrumental techniques (GC–MS or HPLC–MS), especially suitable for on–site detection.

#### 4.1.2 Luminescent Properties Change

Complementing gel–phase transitions, luminescence modulation also constitutes a powerful strategy for visual molecular recognition. Tpy–Zn complexes exhibit various advantages in this regard, including tunable fluorescent emission and high environmental sensitivity, thereby rendering them as ideal platforms for stimuli–responsive sensors [128, 129]. Interaction with specific analytes (metal ions, anions, amino acids, or H^+^) can perturb the coordination environment or electronic distribution, inducing measurable alterations in emission intensity and/or wavelength. These optical alterations, obviously detectable under UV light irradiation or even under the sunlight, enable straightforward and selective molecular recognition without complicated procedures. In this section, representative Tpy–Zn molecules that leverage or alternate emission for guest discrimination are highlighted, emphasizing on plausible mechanisms, structure–property relationships, as well as recognition strategies.

**FIGURE 29:**
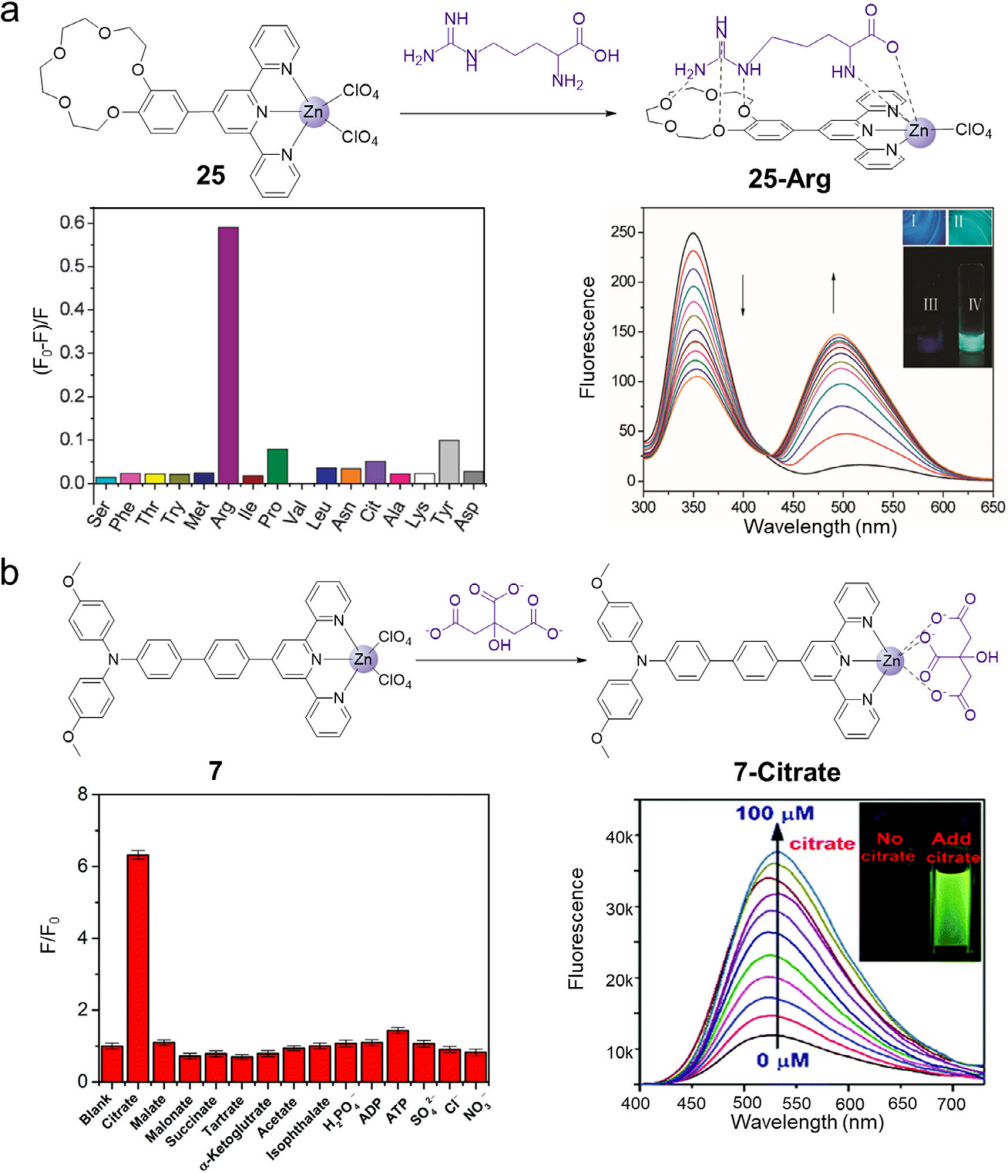
(a) Visual fluorescence recognition of Arg by Tpy–Zn complex **25** via emission intensity variation in the presence of different amino acids. Reproduced with permission [70]. Copyright 2011, Royal Society of Chemistry. (b) Visual fluorescence recognition of citrate by Tpy–Zn complex **7**, with titration–dependent emission response and visual signal under UV light. Reproduced with permission [74]. Copyright 2016, Royal Society of Chemistry.

In 2011, Wu and colleagues developed Tpy–Zn complex **25** with a crown ether fragment at the *para*–position of the central pincer pyridine, enabling selective discrimination of arginine (Arg) by clear fluorescence enhancement in aqueous solution (Figure 29a) [70]. The recognition depends on dual–site cooperative binding: the crown ether engaged the guanidinium group of Arg, while the Zn(II) center coordinated with its amino and carboxyl parts. This cooperative binding, along with size complementarity, conferred highly selective discrimination toward Arg over other amino acids. Furthermore, in 2016, Tian and colleagues reported a Tpy–Zn complex **7** featuring AIE characteristics for the visual detection of citrate (Figure 29b) [74]. Upon addition of citric acid to a THF/H_2_O mixture containing **7**, a pronounced fluorescence enhancement was observed, with a detection limit as low as 3.5 × 10^−7^  M. The outcome was mainly attributed to the AIE effect caused by the selective coordination of –COOH of citrate with Zn(II) center, the resulting adducts **7–citrate** inducing aggregation and restrict intramolecular motion. This approach highlights the advantage of integrating AIE–active systems with Tpy–Zn motifs toward sensitive discrimination of small biological anions in aqueous media.

**FIGURE 30:**
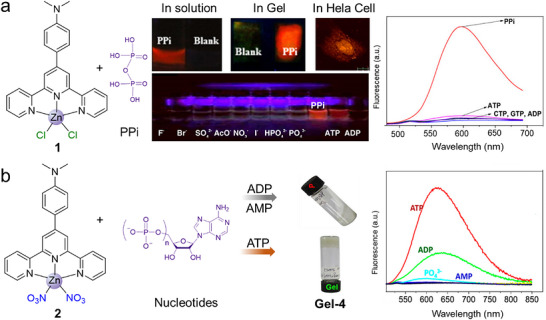
(a) Selective visual recognition of PPi by Tpy–Zn complex **1** via fluorescence emission enhancement in aqueous condition. Reproduced with permission [60]. Copyright 2014, American Chemical Society. (b) Selective gelation behavior of complex **2** to discriminate various nucleic acids (ADP, AMP and ATP). Reproduced with permission [61]. Copyright 2016, American Chemical Society.

Besides direct coordination–induced emission shifts, guest binding can also modulate the supramolecular organization of Tpy–Zn adducts, thereby triggering AIE or other luminescence responses. In 2014, the Rissanen group realized coordination of complex **1** with pyrophosphate (PPi) and triggered molecular aggregation with a ∼500–fold fluorescence enhancement via the AIE process (Figure 30a) [60]. This protocol exhibited high sensitivity (Limit of detection: 20 nM) with feasibility of PPi imaging within the HeLa cells. Moreover, complex **1** could also form gel under acidic conditions and further be processed into disposable strips, which displayed visible orange emission only in the presence of PPi (1.6 µg mL^−1^), whereas, other anions elicited negligible response.

Subsequently, Tu and co–workers employed complex **2** to accomplish selective nucleotide recognition via ATP–triggered gel formation (Figure 30b) [61]. Addition of small amount ATP induced a stable hydrogel **Gel–4** formation, whereas adenosine diphosphate (ADP) and adenosine monophosphate (AMP) failed to trigger gelation under otherwise identical test conditions. The selectivity arises from the distinct coordination geometries of phosphate groups: ATP binding promotes an extended linear molecular conformation that reduces the possible intramolecular π–π stacking, thereby facilitating intermolecular aggregation into an ordered gel network. Importantly, this gelation process was accompanied by a pronounced fluorescence enhancement, as seen in the emission spectra, which is attributable to the restriction of intramolecular motions in the aggregated state—an AIE effect. In contrast, ADP and AMP, which favor folded conformations due to the intramolecular π–π stacking between coordinated planar pincer ring and aromatic moieties of nucleotide, neither gelation nor luminescence enhancement. Thus, the ATP recognition is simultaneously manifested in macroscopic phase transition and amplified optical signals, providing a dual readout mode.

Gel–phase transformations of Tpy–Zn complexes highlight how subtle variations in ligand environment and counterion selection govern supramolecular assembly and optical response. Guest binding reorganizes the coordination network, driving selective gelation or disassembly with concomitant AIE enhancement. Such coupling of molecular design with hierarchical phase behavior enables analyte–specific, rapid, and instrument–free recognition, establishing a versatile platform for biosensing.

### 4.2 Functional Luminescent Materials

Benefiting from their selectively chelating behaviors and subsequent obvious chemical and physical alteration, coordination complexes have also emerged as robust and versatile platforms for constructing functional luminescent materials [130, 131, 132]. Particularly, their photo–responsive behaviors play a critical role, enabling dynamic control over emission characteristics of the resulting materials, which broadens their potential applications [133, 134]. Due to their tunable coordination geometry and photophysical behavior, Tpy–Zn complexes exhibit diverse emission alterations, including fluorescence enhancement, color switching, and even persistent phosphorescence, enabling their potential applications in information encryption, anti–counterfeiting, rewritable displays, and even flexible visual devices [94, 135]. In this section, recent advances in functional luminescent materials prepared from Tpy–Zn complexes are summarized, emphasizing on fabricating strategies, stimuli–responsive behavior, and their feasibility.

**FIGURE 31:**
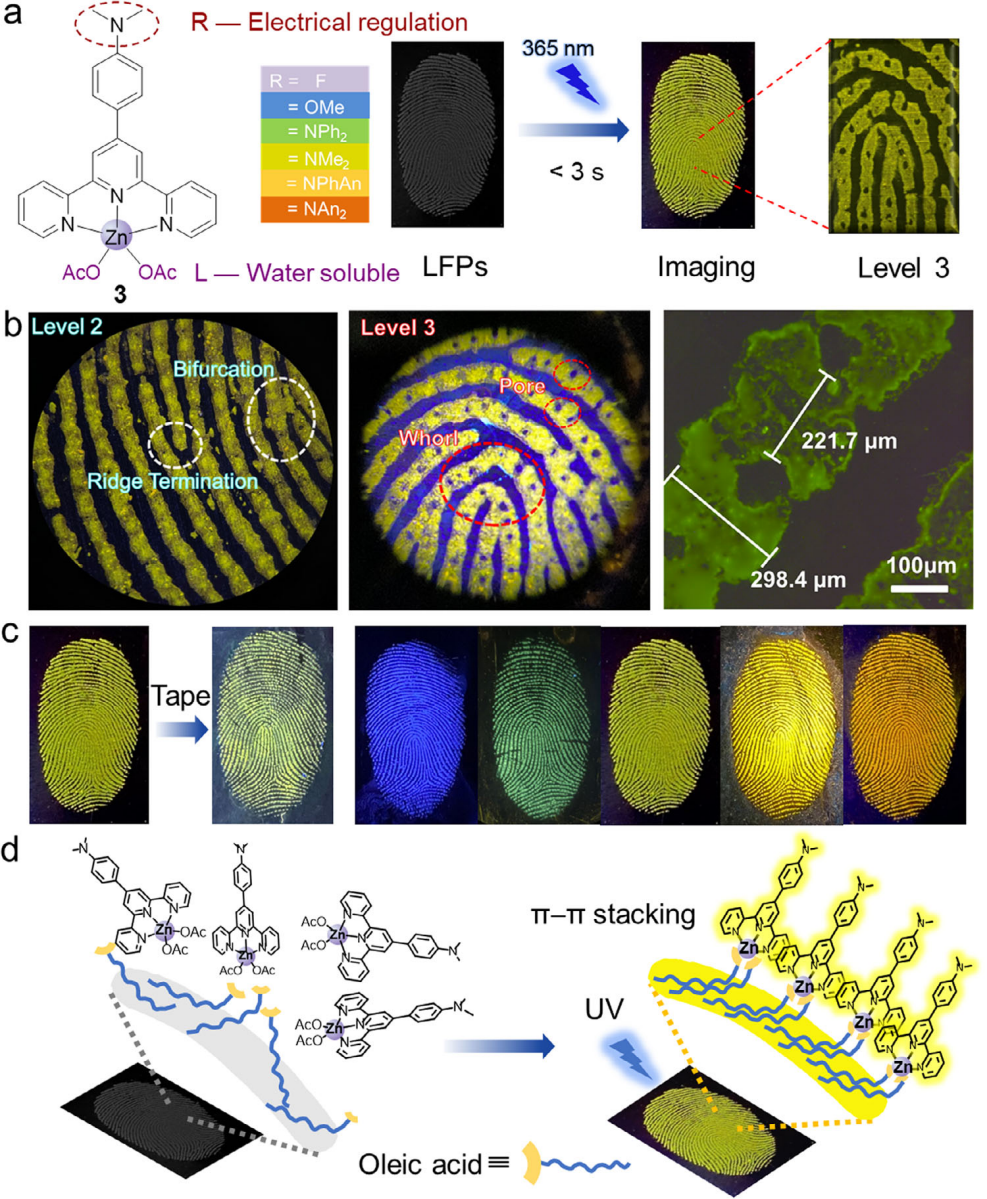
(a) Molecular structure of Tpy–Zn complex **3** with various electron–donating substituents and schematic illustration of instantaneous, high–resolution visualization of latent fingerprints (LFPs) in aqueous media. (b) Level 2 and Level 3 features captured after LFP imaging with **3**. (c) Demonstration of reproducibility and tunable fluorescence emission of LFPs images obtained from different Tpy–Zn complexes. (d) The plausible mechanism diagram of LFPs visualization developing by complex **3**. Reproduced with permission [69]. Copyright 2022, Wiley.

Besides their sensing capabilities in solution–phase, Tpy–Zn complexes have recently exhibited feasibility in solid–state visualization, including latent fingerprints (LFPs) imaging. In 2022, Tu and co–workers developed a series of water–soluble Tpy–Zn complexes bearing electron–donating substituents for LFPs sensing and discrimination. Among them, complex **3** containing a –NMe_2_ group exhibited an “off–on” luminescence behavior (Figure 31a), enabling rapid and high–contrast visualization of LFPs at a concentration as low as 20 µM within 3 s [69]. The emitted yellow fluorescence was bright and stable, allowing clear resolution of level 3 LFPs details (Figure 31b) and reproducible transfer directly using forensic tape. By tuning the electron–donor strength of the substituents, emission color could be adjusted from blue to orange–red (Figure 31c). Mechanistically, the Zn(II) center selectively chelates with lipid components in LFPs, the resulting adducts are readily assembled via π–π interaction and van del Waals interaction between the interphase, leading strong emission due to the AIE effect (Figure 31d). This work offers a rapid, non–destructive, and high–resolution imaging approach for forensic applications, even in water, extending the feasibility of Tpy–Zn complexes beyond visual molecular recognition. The inexpensive, easily handled and low–toxic Zn complex confers significant commercial viability, facilitating smart portable devices for targeted visualization and real–time analysis of LFPs or bioimprinting. Importantly, its imaging ability for high–resolution LFPs at level 3 may provide a powerful tool for constructing advanced Level 3 LFPs databases servicing public security, information encryption, and criminal investigation.

**FIGURE 32:**
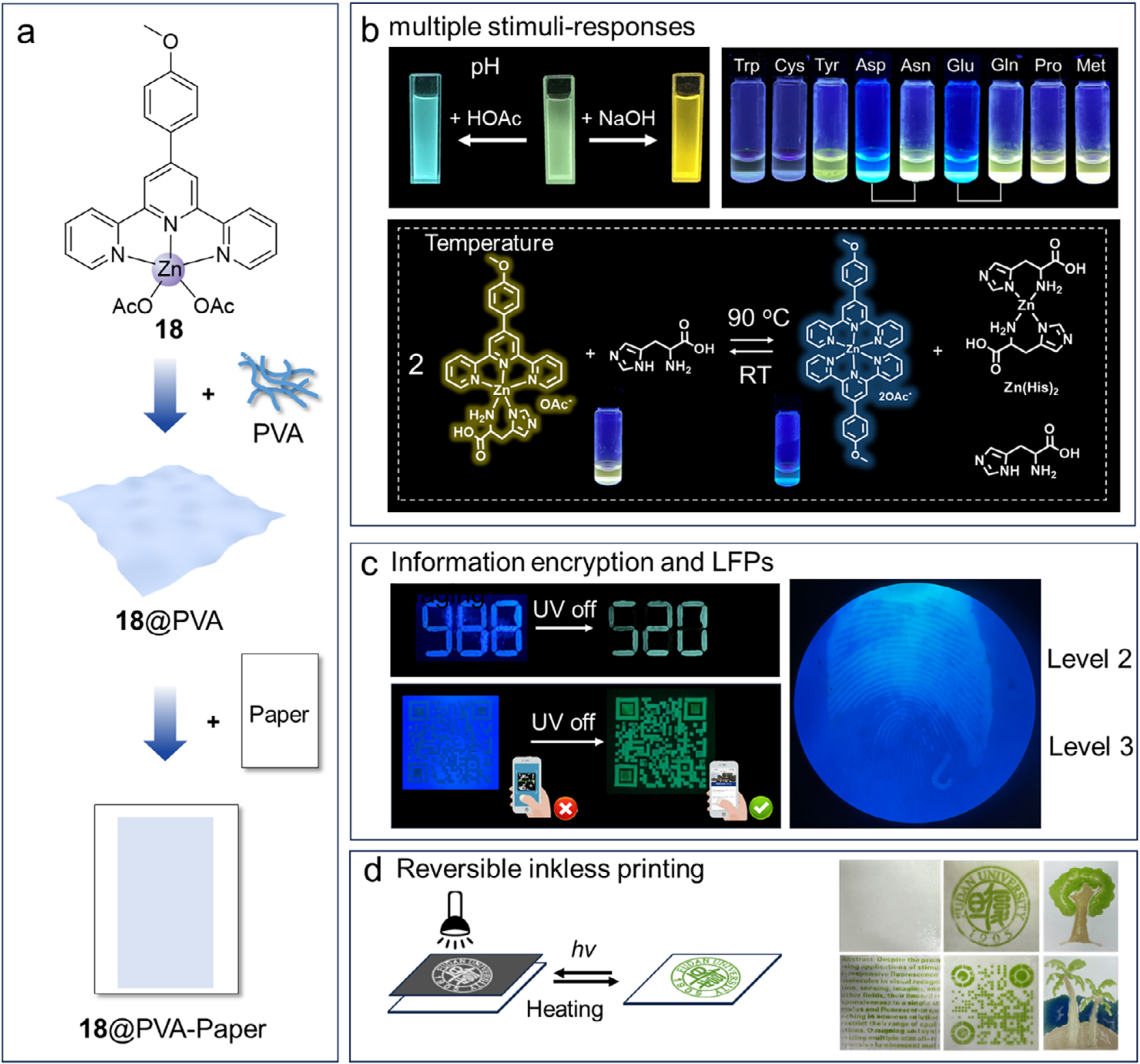
(a) Molecular structure of Tpy–Zn complex **18** and schematic illustration of its integration with poly(vinyl alcohol) (PVA) and paper to form composite materials. (b) pH and amino acid responsiveness of complex **18** in aqueous solution. (c) Applications of PVA–based composites **18**@PVA in information encryption and sweat LFPs visualization. (d) Photochromic behavior of composite **18**@PVA–Paper and application in inkless reversible printing. Reproduced with permission [64]. Copyright 2024, Wiley.

Furthermore, the same group developed a water–soluble D–π–A type Tpy–Zn complex **18**, exhibiting multi–stimuli responsiveness to water, pH, and specific amino acids in 2024 (Figure 32a) [64]. Notably, the system showed reversible thermochromic behavior in the presence of histidine (His), with fluorescence color shifting from yellow–green to blue upon heating (Figure 32b). Spectroscopic and computational analyses revealed that Zn(II) coordination mode conversion triggered by ligand substitution and molecular aggregation is responsible for the stimuli–responsive luminescence switching. Consequently, they accomplished visual recognition of amino acid pairs (Asp/Asn, Glu/Gln), and selectively identified His via temperature–depended luminescence switching (Figure 32b). To enhance their practical utilization, corresponding flexible “PVA–paper–based” composite was then fabricated, yielding stable room–temperature phosphorescence (lasting up to 14 s), water–responsive fluorescence, and reversible photochromism, with potential applications in information encryption, anti–counterfeiting, LFPs recognition (Figure 32c), and inkless reversible printing (Figure 32d). After rational molecular design and dopping with other substances, this study demonstrates the possibility of fabricating “smart” multifunctional composites from simple complexes, which not only extends the practical applicability of Tpy–Zn complexes, but also provides a new strategy to access intelligent luminescent materials with programmable optical outputs due to their stimuli–responsive behaviors.

**FIGURE 33:**
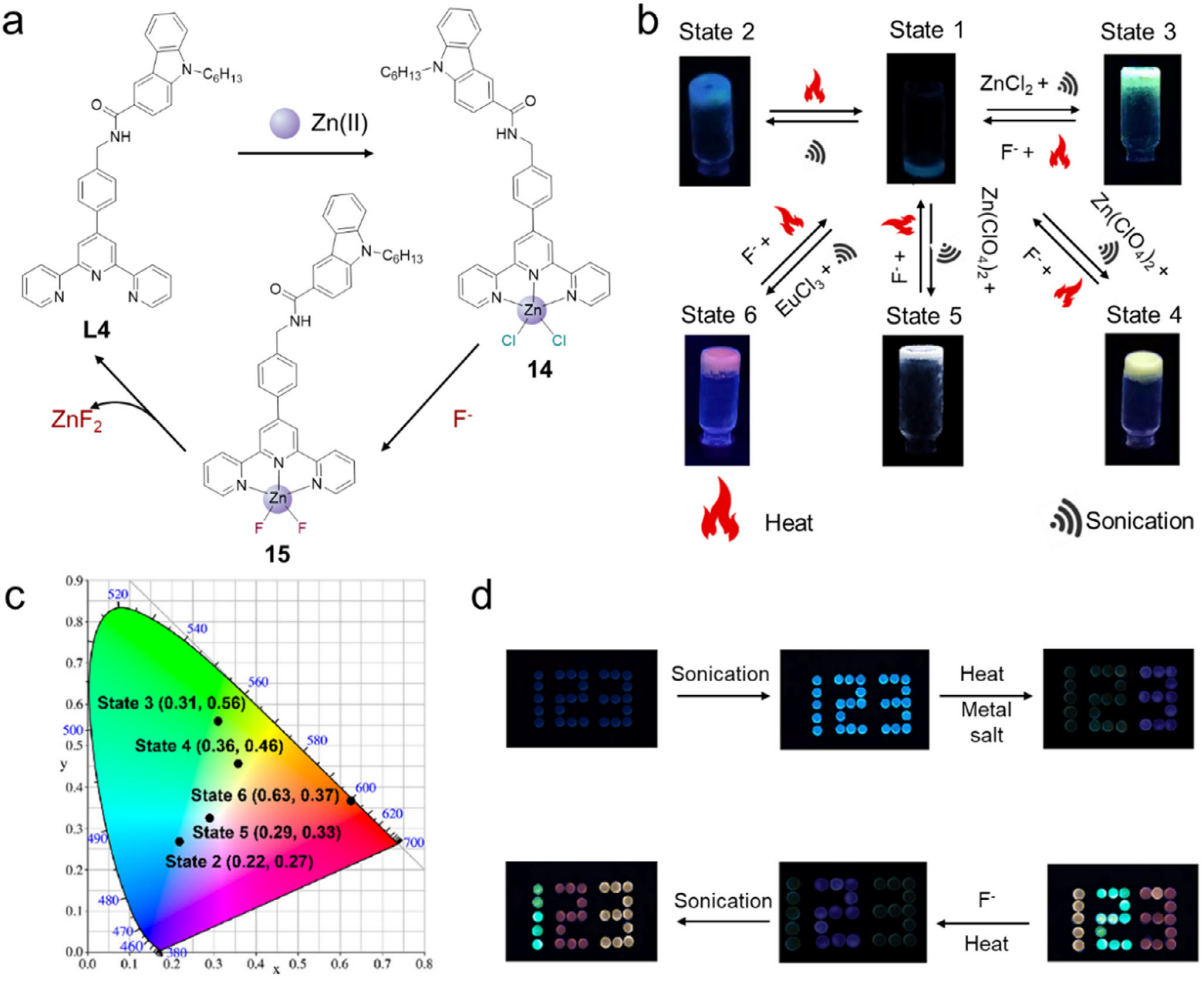
(a) Schematic diagram illustrating the principle of adjusting the fluorescence emission color through the Tpy ligand **L4** and the dynamic metal–ligand coordination principle. (b) Photos showing the mutual conversion of six states (fluorescence quenching, blue, green, yellow, red, white) under UV irradiation. (c) CIE–1931 chromaticity diagrams of different fluorescence emission states. (d) Schematic diagram of the application of multi–color fluorescence emission phenomenon in reversible encoding and decoding. Reproduced with permission [68]. Copyright 2020, Chinese Chemical Society.

In 2021, Huang and Zhao reported another Tpy ligand **L4** with a coumarin group, which is capable of forming reversible coordination complexes with Zn(II) salts and undergoing ultrasound– or heat–induced gelation in aqueous media (Figure 33a) [68]. By utilizing anion–triggered ligand–exchange strategy, multicolor luminescence ranging from blue to yellow (Figure 33b), including nearly ideal white light emission (Figure 33c), was observed. This dynamic and reversible color modulating enabled practical applications in encryption and decryption. Mechanistic studies revealed that the emission color was modulated by the intramolecular charge transfer (ICT) process, which was sensitive to the electronegativity of the coordinated counterions. More electronegative anions like F^–^ ions induced distinct shifts in emission wavelength by altering the coordination environment around the Zn(II) center. Meanwhile, this work demonstrates a versatile strategy for constructing tunable luminescent systems, offering new avenues for accessing responsive optical materials in data storage, anti–counterfeiting, and smart display technologies (Figure 33d).

**FIGURE 34:**
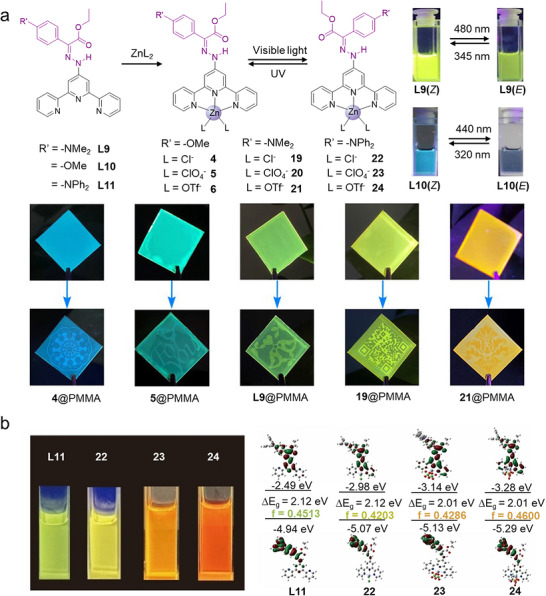
(a) Molecular structure of the Tpy ligand **L9–L11** and its corresponding complexes **4–6**, and **19–24**, as well as photo–induced isomerization phenomenon and the photochromic phenomenon of those complexes for inkless printing. Reproduced with permission [65]. Copyright 2022, Wiley. (b) Fluorescence images (left) and diagrams of the change of counterions on the HOMO and LUMO energy levels (Right) of Tpy ligand **L11** and its corresponding complexes **22–24**. Reproduced with permission [66]. Copyright 2023, Wiley.

Furthermore, photo–induced isomerizable Tpy ligands were also involved to demonstrate their feasibility of Tpy–Zn complexes in dynamic luminescent materials. As shown in Figure 34a, photo–reversible *Z/E* isomerization of ligands **L9, L10** and their Zn(II) salts are readily con, trolled by intramolecular hydrogen bonding, along with significantly altering their fluorescence intensity [65]. After embedding into PMMA films, the resulting tunable emission colors enabled high–resolution, erasable fluorescent patterning, highlighting their potential in inkless printing and secure data encryption. Subsequently, Zhao and Ma further prepared ligand **L11** featuring a triphenylamine–hydantoin fragment [66]. Complexes **22–24** exhibited anion–dependent fluorescence (Figure 34b). DFT calculations confirmed emission shifts originated from modified ICT processes and frontier orbital energy realignment. These works reveal how photo–responsive fragments can be integrated with anion–modulated coordination to fabricate intelligent luminescent systems with reversible and multicolor performance.

**FIGURE 35:**
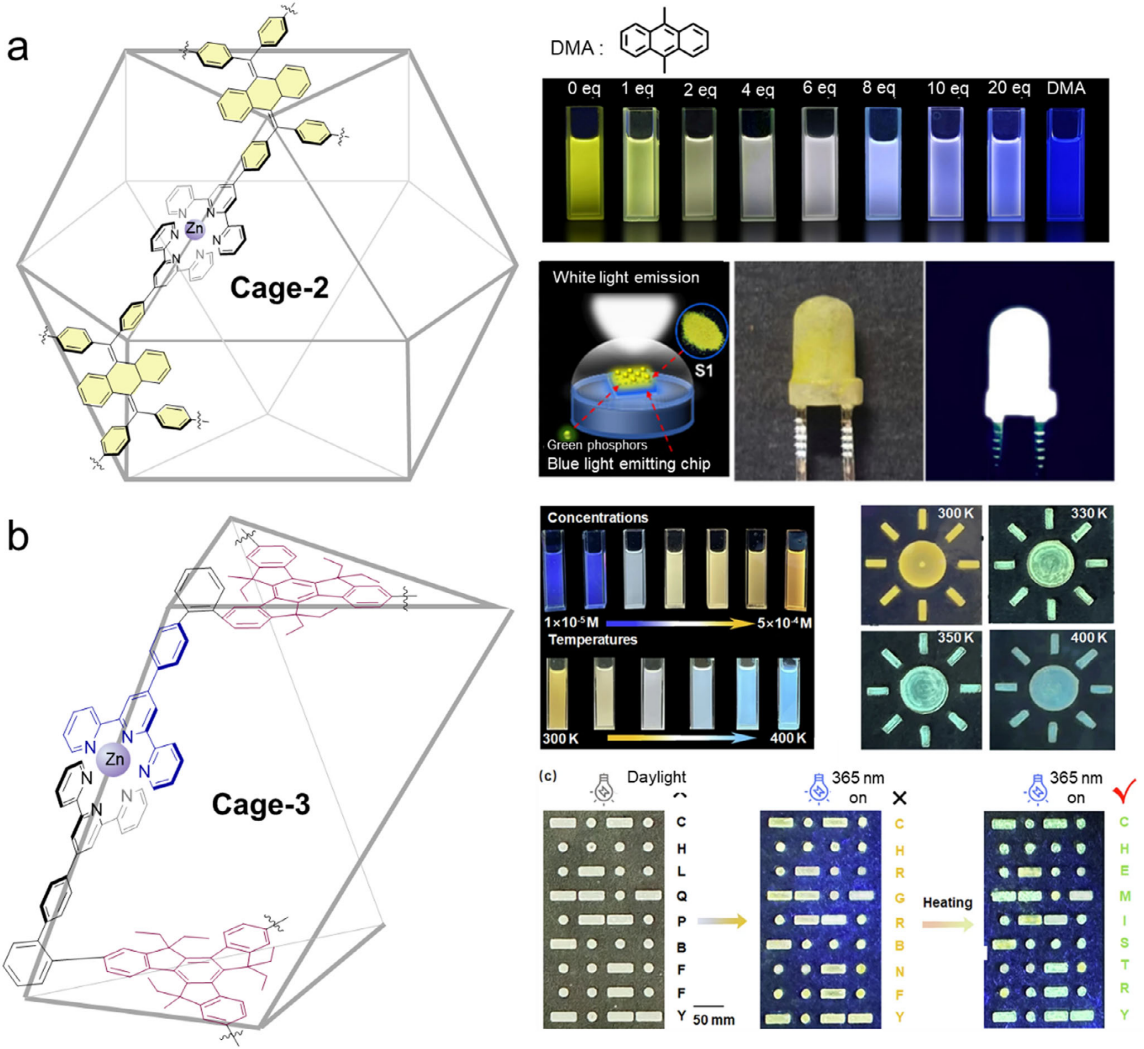
(a) Structure of **Cage–2** and its application in white LED light–emitting devices. Reproduced with permission [62]. Copyright 2022, American Chemical Society. (b) Structure of **Cage–3** and its multiple stimulus responsiveness to temperature, as well as the application of its composite in thermal activation information encryption. Reproduced with permission [63]. Copyright 2024, Springer Nature.

Beyond simple complexes, supramolecular cages formed by Tpy–Zn coordination have also gained prominence due to their precise and sophisticated structures with multifunctionalities. These architectures offer confined microenvironments, enhanced rigidity, and multivalent coordination sites leading to the resulting luminescent systems with superior stability, tunability, and stimuli–responsiveness. In 2022, Wang et al. fabricated **Cage–2** (Figure 35a), which exhibits AIE property. Dopant–dependent tuning with blue–emissive 9,10–dimethylanthracene (DMA) achieved multicolor fluorescence and white–light emission (CIE: 0.32, 0.34) [62]. **Cage–2** also exhibited a high solid–state quantum yield (Φ = 23.4%) and could be directly applied as an LED coating, generating bright white light from a yellow–emitting film. Subsequently, Wang and Zhang developed dopant–free helical **Cage–3** (Figure 35b), achieving concentration–dependent white–light emission via monomer–to–excimer transition (blue to orange) [63]. Embedding **Cage–3** into PMMA formed a temperature–responsive composite with the potential toward thermal sensing and information encryption.

**FIGURE 36:**
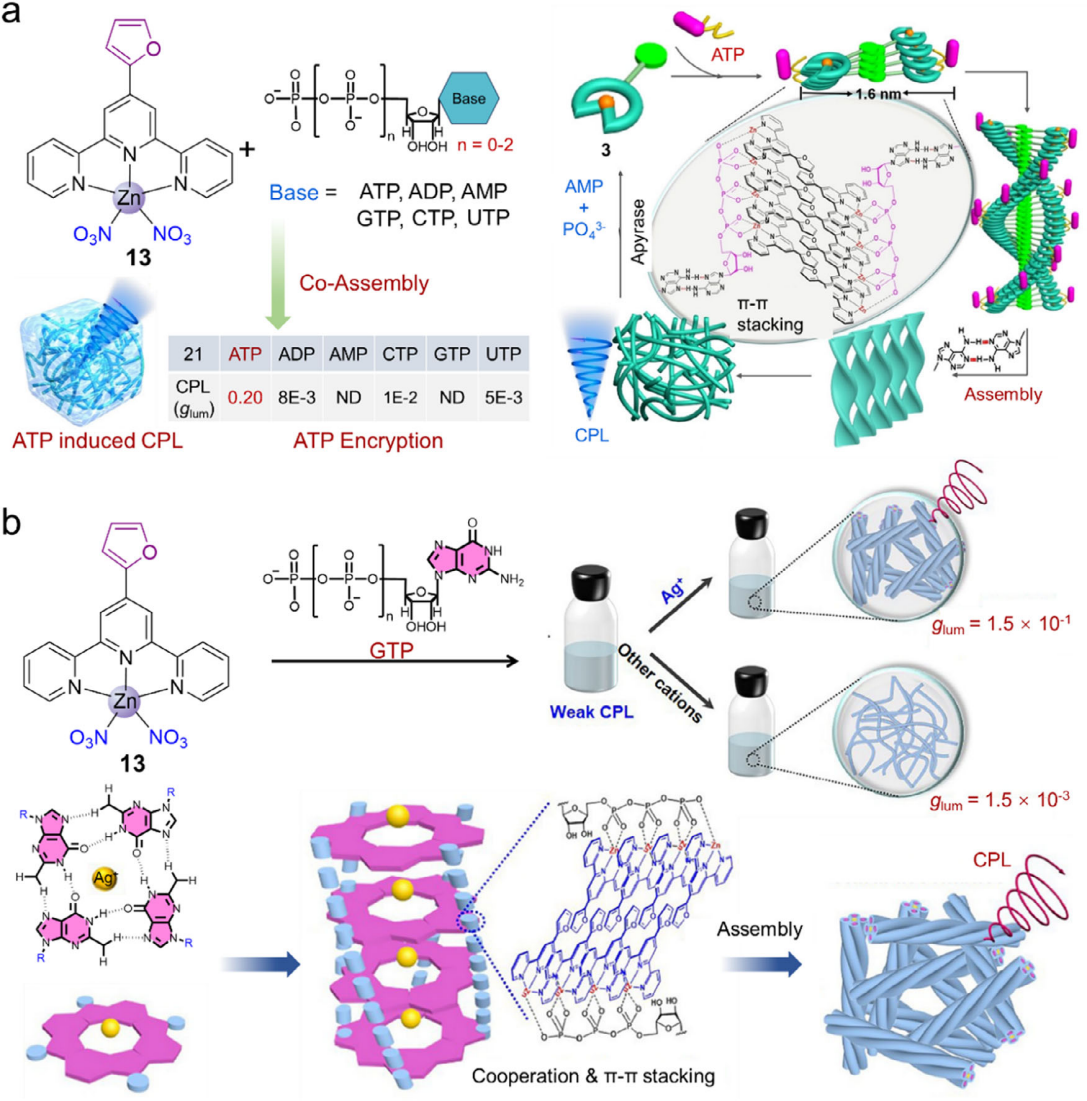
(a) Schematic illustration of the co–assembly mechanism between Tpy–Zn complex **13** and various nucleotides, and the corresponding CPL signal of the resulting gel materials. Reproduced with permission [141]. Copyright 2022, Wiley. (b) CPL modulation of the co–assembly system formed by complex **13** and GTP under regulation of Ag(I) ions. Reproduced with permission [142]. Copyright 2025, Science China Press.

Circularly polarized luminescence (CPL) materials have garnered significant interest for advanced optoelectronics, including 3D displays [136, 137], encrypted data storage [138, 139], and sensing [140]. In 2022, Liu and co–workers disclosed that a supramolecular gel formed by complex **13** with ATP, exhibiting exceptional CPL activity (g_lum_ = 0.20, Figure 36a) [141]. Mechanistic studies revealed furan–phosphate π–π interactions drove helical nanofiber formation, facilitating chirality transfer from nucleotide to the luminescent assembly. The CPL signal was reversibly modulated by ATP hydrolysis, enabling bio–responsive encryption. In 2025, they further demonstrated that Guanosine triphosphate (GTP) and Ag(I) co–assembled with **13** via Ag(I)–stabilized G–quadruplexes (Figure 36b), generating chiral helices with enhanced CPL for enantioselective penicillamine sensing [142]. The phosphate groups in GTP were found to play a key role in guiding the supramolecular packing, while Ag(I) served as a structural organizer to induce long–range chirality. These studies demonstrated phosphate–containing biomolecules as dual–functional co–assemblers for engineering supramolecular chirality in Tpy–Zn systems. Future works should pursue programmable CPL platforms with switchable handedness, multi–stimuli responsiveness, and broader biosensing/encryption utility.

**FIGURE 37:**
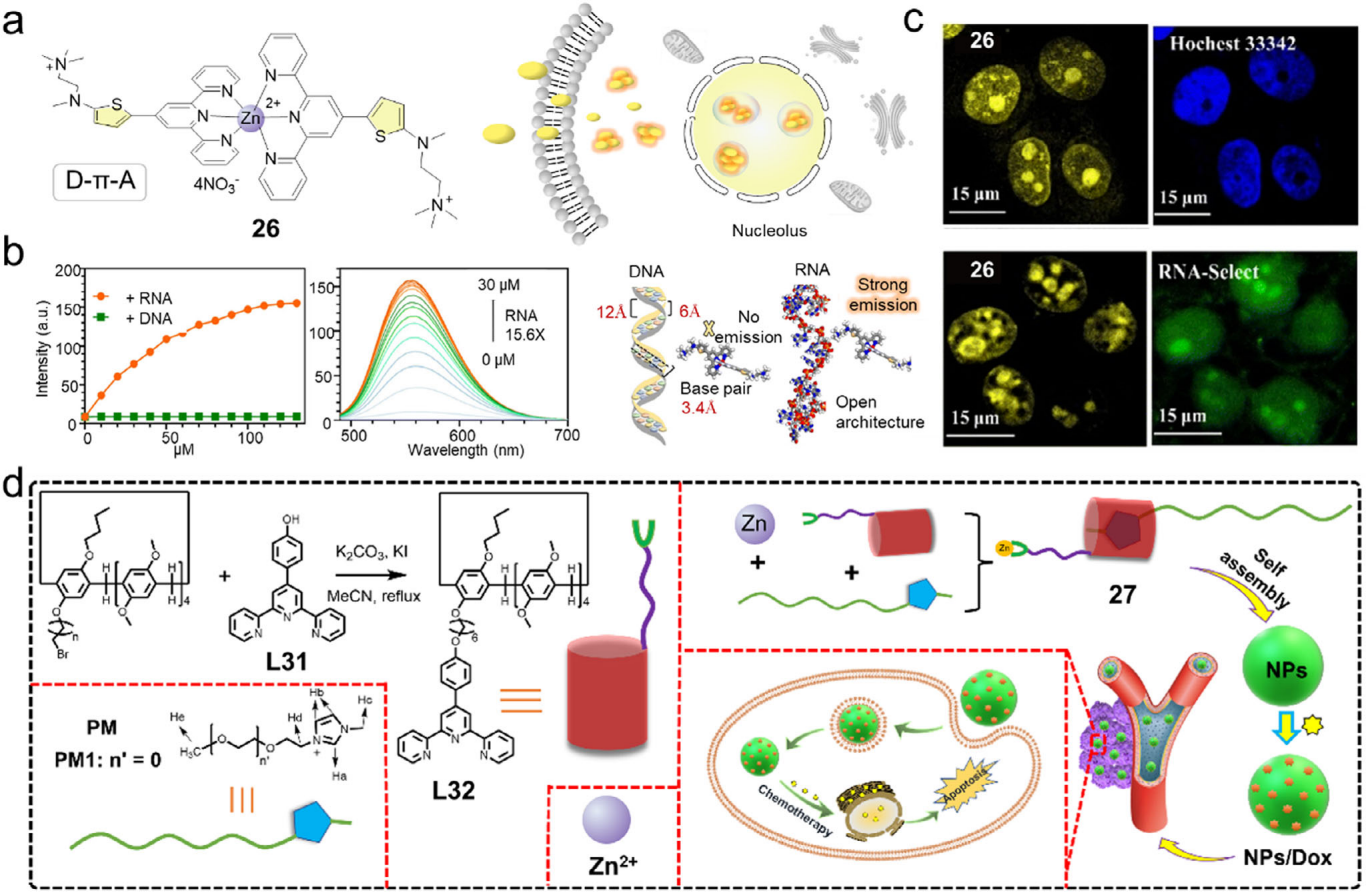
(a) Molecular structure of complex **26** and schematic diagram of imaging RNA in living organisms. (b)  Emission spectra and binding selectivity of **26** toward nucleic acids. Left: Fluorescence titration curve of **26** with increasing concentrations of RNA (orange) and DNA (green). Right: Emission spectra of **26** with different RNA concentrations. (c) Confocal fluorescence images of HepG2 cells treated with **26** and colocalization dye Hochest 33342 and RNA–Select. Reproduced with permission [143]. Copyright 2023, Royal Society of Chemistry. (d) Assembly diagram of Tpy ligand **L32** with Zn(II) into a supramolecular adduct **27**, and its application in Dox encapsulation, targeted delivery, and pH–responsive release for anticancer therapy. Reproduced with permission [144]. Copyright 2022, MDPI.

In 2023, Tian and colleagues developed a D–π–A structured Tpy–Zn complex **26** (Figure 37a) with octahedral coordination geometry containing hydrophilic quaternary ammonium groups [143]. The cationic moiety enhanced its aqueous solubility, cell–membrane permeability, and electrostatic RNA affinity. Under physiological pH, complex **26** exhibited 15.6–fold fluorescence enhancement with RNA, significantly outperforming amino acids, ATP, DNA, and BSA (< 1.3–fold change) (Figure 37b). Confocal fluorescence imaging further confirmed its high selectivity toward RNA in living cells: HepG2 cells treated with complex **26** showed strong cytoplasmic luminescence that overlapped well with the RNA–specific dye RNA–select, while exhibiting minimal colocalization with the nuclear stain Hoechst 33342 (Figure 37c). Mechanistic study attributed this specificity to hydrophobic and π–π stacking interactions between the ligand side chains of **26** and RNA bases, leading to restricted intramolecular motion and AIE–type emission.

Owing to their photophysical features and precise modular design, Tpy–Zn complexes have emerged as promising candidates for biological imaging. Their robust coordination frameworks confer excellent water solubility and photostability, while enabling either AIE or selective luminescence responses toward specific biological guests. Integrating rational recognition motifs and supramolecular assembly, these systems have been successfully applied as fluorescent probes for cellular visualization and real–time molecular tracking.

Complex **26** demonstrated exceptional cellular performance, establishing D–π–A Tpy–Zn complexes as precision platform for RNA–targeted diagnostics. Following the selectivity of D–π–A complexes in intracellular RNA imaging, Yao and co–workers developed an amphiphilic supramolecular probe **27** derived from Tpy ligand **L32** with Zn(II) ions (Figure 37d) [144]. And the final theranostic probe was obtained by encapsulating fluorescent nanoparticles and doxorubicin (Dox) through synergistic host–guest and supramolecular interactions. In contrast to previous RNA–targeted imaging systems, this design integrated pH–responsive drug release under the acidic tumor microenvironment. This strategy enabled selective RNA imaging combined with acid–triggered drug release, achieving spatiotemporally controlled theranostic functions within tumor cells, highlighting the potential of Tpy–Zn complexes for programmable intelligent nanomedicine.

### 4.3 Photocatalysis

Besides their versatile luminescent properties, Tpy–Zn complexes exhibit competitive photophysical characteristics, including strong visible–light absorption, tunable excited–state lifetimes, and high photo–stability, which make them emerging photocatalysts for accelerating solar–driven transformations. These features enable Tpy–Zn systems to function as integrated light–harvesting antennas, photosensitizers, and catalytic centers for visible–light–driven conversions. This section highlights recent advances in Tpy–Zn photocatalysts, disclosing structure–function correlations and mechanistic principles governing their performance.

#### 4.3.1 Photocatalytic CO_2_ Reduction

The excessive emission of CO_2_, primarily from fossil fuel combustion and industrial processes, has become a major driver of global climate change [145]. Photocatalytic CO_2_ reduction offers a sustainable solution to mitigate carbon emissions while generating value–added fuels and/or chemicals. Tpy–Zn complexes have emerged as promising catalysts for CO_2_ photoreduction, because of their tunable electronic structures, strong coordination capacity, and light–harvesting capability [110]. Recent advances have demonstrated that the rational design of Tpy ligands and corresponding coordination assemblies, such as polymeric frameworks and supramolecular architectures, can significantly enhance their catalytic performance. The following examples illustrate how coordination geometry, electronic modulation, and porous structure collectively contribute to the development of efficient Tpy–Zn based photocatalytic systems for CO_2_ reduction.

**FIGURE 38:**
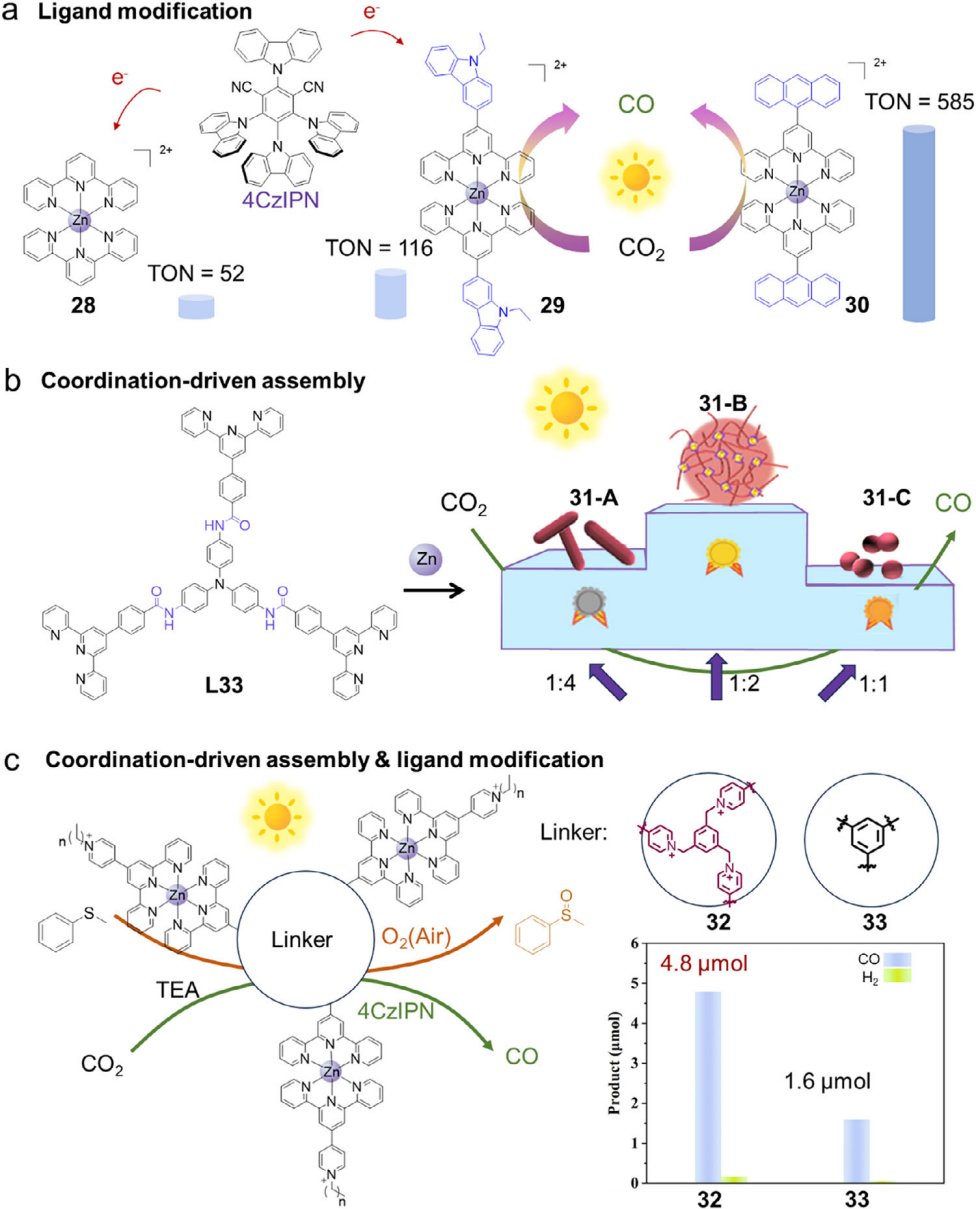
(a) Structural modification of complex **28** yielding complexes **29** [146], and **30** [147], and comparison of their catalytic performance in CO_2_ photoreduction. (b) Coordination of Tpy ligand **L33** with Zn(II) forming nanostructures (**31–A/B/C**) with distinct morphologies and schematic illustration of their application in photocatalytic CO_2_ reduction. Reproduced with permission [148]. Copyright 2023, American Chemical Society. (c) Tpy–Zn coordination polymer **32** and its derivative **33**, and their catalytic applications in photooxidation of organic substrates and photocatalytic CO_2_ reduction. Reproduced with permission [58]. Copyright 2023, Royal Society of Chemistry.

It is worth noting that the closed–shell d^10^ electronic configuration of Zn(II) leads to redox inertness of the metal center, posing a challenge for its use in CO_2_ photoreduction. To address this limitation, the Chao group employed ligand modification strategies, shifting the redox activity from the metal center to the ligand framework. In 2023, they synthesized a coumarin–functionalized Tpy–Zn complex **29** with a D–A structure (Figure 38a) [146]. This modification extended the π–conjugation moiety of the ligand, resulting in a more negative ligand–centered reduction potential and enhanced stabilization of the Tpy radical intermediate (λ_max_ = 660 nm). As a consequence, complex **29** achieved a turnover number (TON) of 116 and a CO selectivity of 95.2%, significantly higher than that of unmodified complex **28**. The photocatalytic reaction was carried out under visible light in the presence of a photosensitizer (4CzIPN), which provides the required excited electrons. Building on this success, the group further synthesized an anthracene–substituted Tpy–Zn complex **30** in 2025 [147], which further extended the π–system and demonstrated better photocatalytic performance with a TON of 585 and a CO selectivity of 96.2%, nearly 10 times higher than complex **28** (Figure 38a). This work highlights the power of ligand modification in overcoming the redox inactivity of Zn(II) and significantly enhancing CO_2_ photoreduction efficiency.

Besides ligand modification, coordination assembly represents an effective strategy for controlling structural morphology. In 2023, the same group constructed Tpy–Zn supramolecular assemblies by employing a flexible tris–Tpy ligand **L33** containing amide linkages, and systematically tuning the metal–to–ligand coordination ratio (Figure 38b) [148]. By adjusting the Zn(II)/ligand ratio from 1:1 to 1:4, they realized precise morphological control, yielding spherical nanoparticles (**31–A**), fibrous gels (**31–B**), and rigid microrods (**31–C**). Among these, the fibrous gel **31–B** exhibited the highest photocatalytic efficiency for CO_2_–to–CO conversion under visible light, with a production rate of 6.3 mmol g^−^^1^ h^−^^1^ and a selectivity of 94.7%. Mechanistic studies revealed that the enhanced activity of **31–B** stemmed from its hierarchical fibrous network, which was stabilized by Tpy–Zn coordination, hydrogen bonding, and π–π stacking. This architecture facilitated directional charge transport and efficient substrate diffusion, thus boosting the overall catalytic performance. This study highlights how coordination–driven morphological control can tailor the physicochemical environment of the catalytic interface, providing a rational design strategy for the construction of structurally adaptive, high–performance photocatalytic platforms.

To further elevate the photocatalytic performance of Tpy–Zn systems, the Chao team combined ligand modification and coordination–driven assembly by incorporating a redox–active viologen (pyridinium) unit into a newly designed Tpy ligand. Through coordination with Zn(II) ions, the functionalized ligand was readily assembled into a polymeric network, designated as coordination polymer **32** (Figure 38c) [58]. This dual–strategy design endowed the material with outstanding photocatalytic activity, enabling efficient visible–light–driven CO_2_ reduction (CO yield: 4.8 µmol, 96% selectivity). Mechanistic studies revealed that the Tpy radical anion (Tpy**·** ^−^) served as the genuine active site for CO_2_ activation, while the incorporated viologen moiety facilitated intramolecular electron transfer, enhancing the redox environment within the polymer. To further elucidate the role of the viologen group, a control polymer **33** lacking this unit was synthesized. The extremely inferior activity of **33** (CO yield: 1.6 µmol) confirmed the critical contribution of viologen to the enhanced photocatalytic behavior.

#### 4.3.2 Photocatalytic Oxidation

Notably, the catalytic performance of polymer **32** can also accelerate visible–light–driven oxidation of organic substrates, even without the need for additional oxidants (Figure 38c) [58]. This dual–functionality highlights their powerful photocatalytic potential of Tpy–Zn systems beyond CO_2_ reduction. In the following part, we explore recent advances in the photocatalytic oxidation with Tpy–Zn catalysts, particularly in green and efficient oxidative transformations.

**FIGURE 39:**
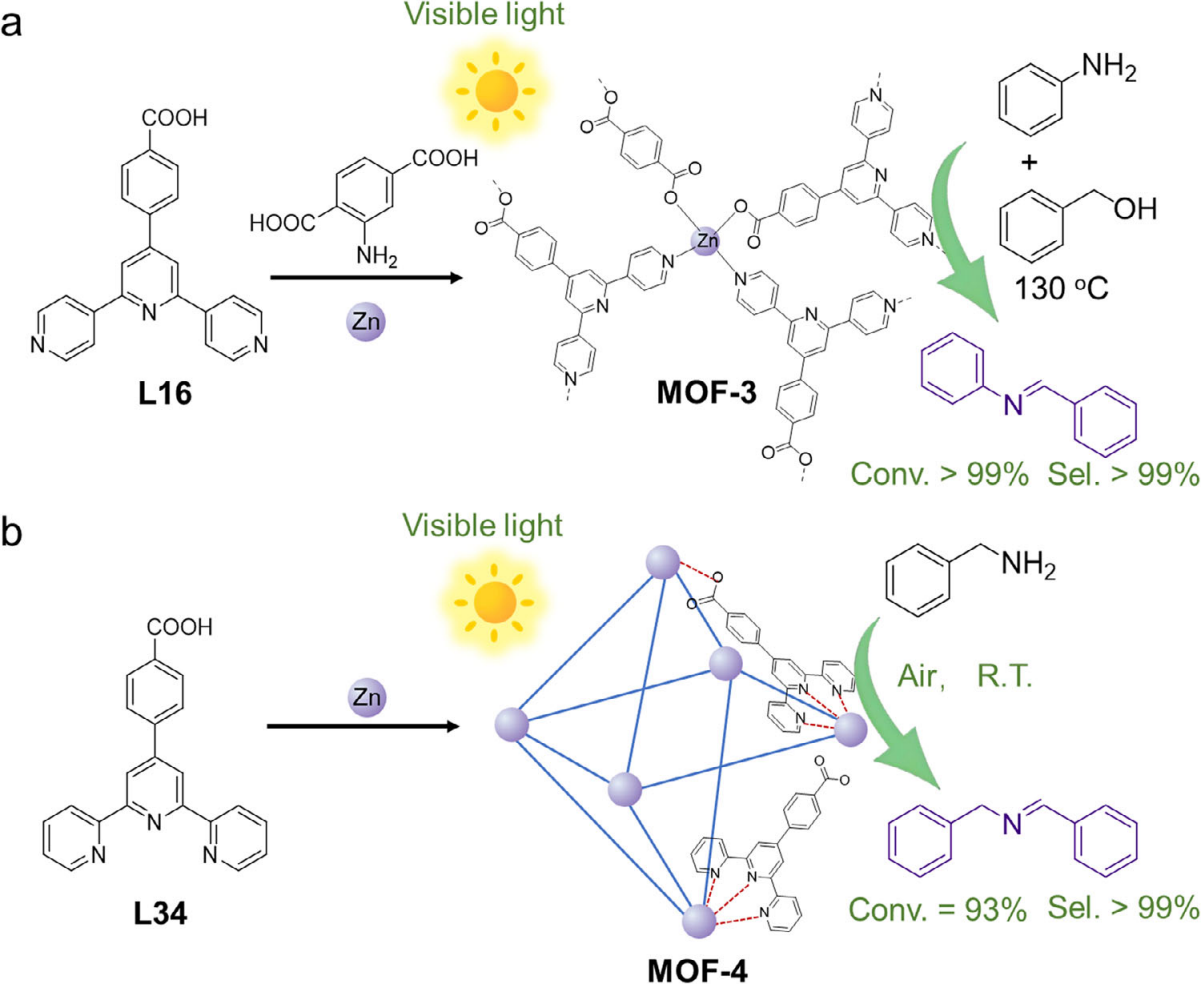
(a) Structure of **MOF–3** and its application in photocatalytic oxidation of small organic molecules [109]. (b) Schematic illustration of **MOF–4** constructed with Tpy–Zn motifs as “molecular antennae” for visible–light–driven photocatalytic oxidation reactions [72].

Very recently, Bagher and co–workers synthesized **MOF–3** by coordinating a Tpy ligand **L16** with Zn(II) and amino benzoic acid under solvothermal conditions (Figure 39a) [109]. The resulting material possessed a 3–fold interpenetrated 3D framework with 40.6% porosity, enabling efficient diffusion and activation of substances. In the oxidation of benzyl alcohol and aniline, **MOF–3** achieved nearly complete conversion under visible light at 130 °C, with 99% selectivity toward imine products. Mechanistic studies revealed that Zn(II) facilitated substrate adsorption and polarization, while its weak hydrogenation ability helped suppress over–reduction. Besides, Tan group integrated light–harvesting Tpy–Zn units into the MOFs to enhance optical response. As shown in Figure 39b, they synthesized **MOF–4** using a carboxylated Tpy ligand **L27** with modified nitrogen positioning as the organic linker and Zn(II) as the metal node [72]. Under visible light and ambient air conditions, **MOF–4** efficiently catalyzed the oxidative coupling of aniline with a 93% conversion and 99% selectivity. The catalyst also showed high reusability, maintaining performance over seven runs. Mechanistic studies confirmed that the Tpy–Zn moiety acts as a “molecular antenna”, enhancing light absorption and promoting charge separation by suppressing recombination. All the outcomes validate the potential of MOFs derived from Tpy–Zn in selective oxidation via light–driven catalytic processes, and provide valuable insights for the design of efficient, reusable, and visible–light–active MOFs catalysts for green organic synthesis.

**FIGURE 40:**
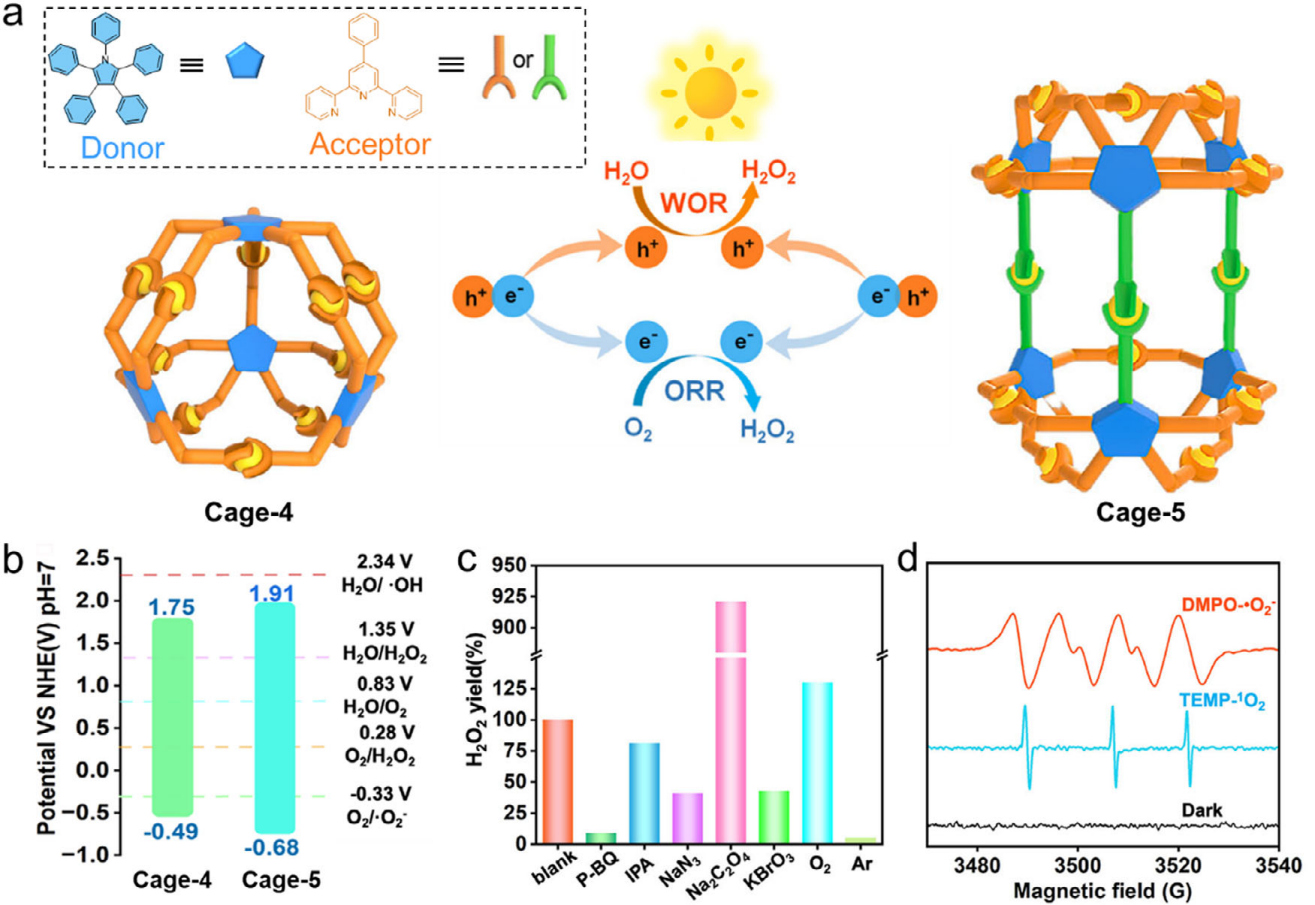
(a) Schematic diagram of supramolecular assemblies **Cage–4** and **Cage–5** for photocatalytic WOR and ORR. (b) Band structure diagrams of **Cage–4** and **Cage–5**. (c) Photocatalytic system of **Cage–5** with different substances. (d) EPR spectra of DMPO–**·**O_2_^−^ and TEMP–^1^O_2_. Reproduced with permission [83]. Copyright 2025, Wiley.

In the realm of photocatalytic production, traditional systems often suffer from limited substrate accessibility and slow oxygen reduction kinetics. Very recently, Li and Wang groups demonstrated the photocatalytic potential of **Cage–4** and **Cage–5** in hydrogen peroxide (H_2_O_2_) production (Figure 40a) [83]. Both cages acted as efficient single–molecule photocatalysts capable of driving oxygen reduction reaction (ORR) and water oxidation reaction (WOR) under light irradiation. Mott–Schottky analysis revealed that **Cage–5** possessed a more positive conduction band minimum (CBM) and a more negative valence band maximum (VBM) compared with **Cage–4**, suggesting a stronger driving force for both reduction and oxidation processes (Figure 40b). Mechanistic studies confirmed that O_2_ played a key role in the photocatalytic cycle: replacing air with argon markedly suppressed H_2_O_2_ generation, while an O_2_–rich atmosphere greatly enhanced the reaction rate (Figure 40c). Further radical–trapping and electron paramagnetic resonance (EPR) experiments verified that both ·O_2_^−^ and singlet oxygen (^1^O_2_) participated in the reaction pathway (Figure 40d). These results provide compelling evidence that Tpy–Zn based cages can efficiently mediate light–driven redox reactions through well–defined molecular frameworks, offering a new design paradigm for efficient photocatalytic systems.

#### 4.3.3 Photocatalytic Water Splitting

In addition to reduction and oxidation, the photocatalytic splitting of water into hydrogen and oxygen represents a cornerstone reaction for achieving a sustainable economy. As a clean, abundant, and carbon–neutral fuel, hydrogen generated from water using solar energy offers a compelling route toward mitigating reliance on fossil fuels [149, 150, 151]. However, efficient water splitting requires photocatalysts that can absorb visible light, mediate multi–electron transfer processes, and remain stable under aqueous conditions [152, 153]. Tpy–Zn based systems have recently garnered attention in this context due to their tunable optoelectronic properties, modular ligand design, and ability to form coordination polymers or extended frameworks with favorable charge transport characteristics. The following cases illustrate how structural engineering of Tpy–Zn materials, ranging from D–π–A type conjugated polymers to supramolecular gels, enable visible–light–driven water splitting, thereby expanding the functional scope of this coordination platform toward solar fuel generation.

**FIGURE 41:**
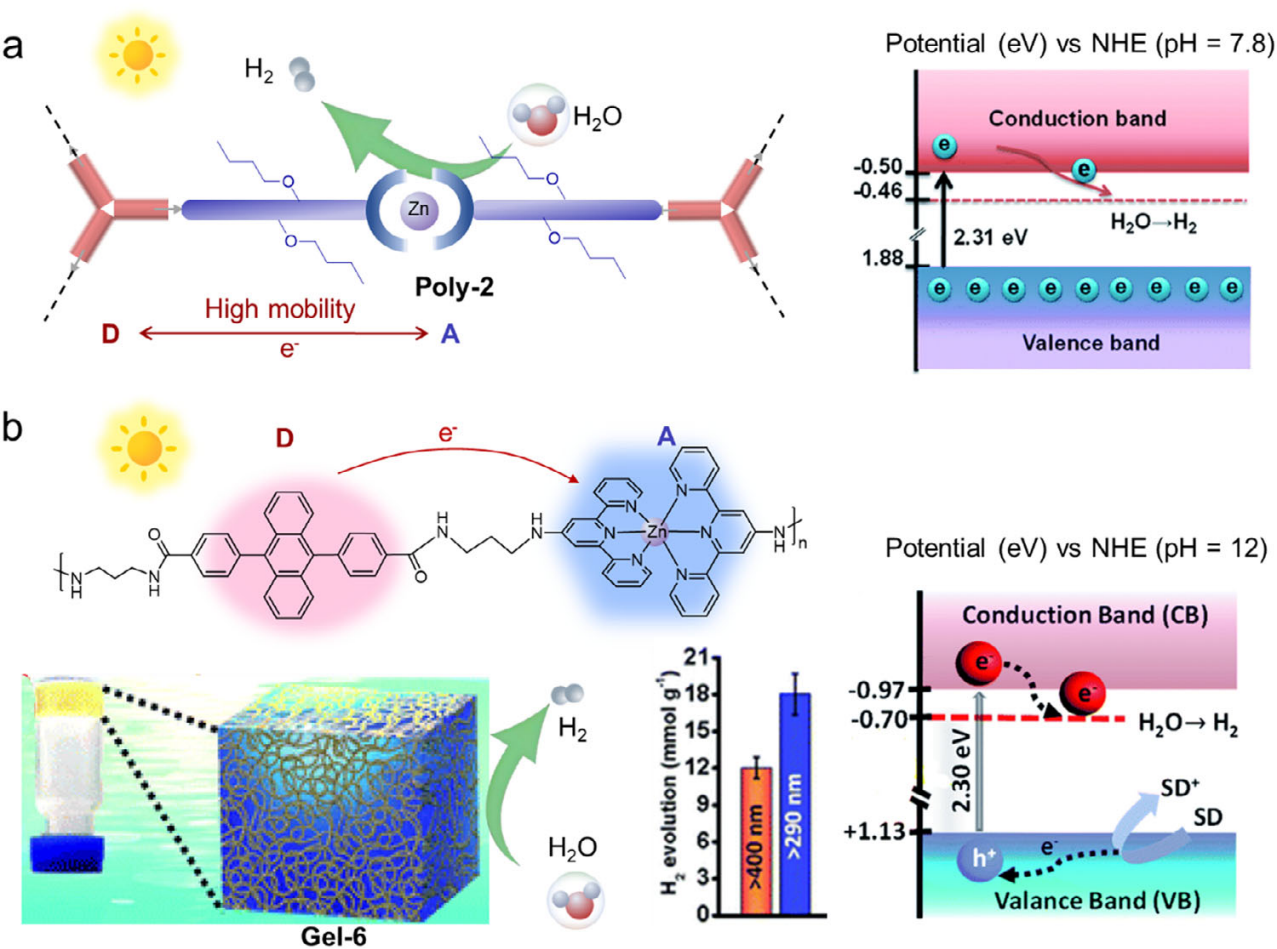
(a) Structure and photocatalytic hydrogen evolution of D–π–A type coordination polymer **Poly–2** under visible light. Reproduced with permission [81]. Copyright 2020, Royal Society of Chemistry. (b) Structure and photocatalytic performance of Tpy–Zn based **Gel–6**, including light–induced electron transfer process, gel morphology, and H_2_ production under different light ranges. Reproduced with permission [154]. Copyright 2021, Royal Society of Chemistry.

In 2020, Ghorai and co–workers developed a D–π–A–type Tpy–Zn coordination polymer, **Poly–2** (Figure 41a), which features a porous and fibrous architecture with a surface area of 234.5 m^2^ g^−1^, enabling efficient light harvesting, charge separation, and reactant diffusion [81]. Under visible–light irradiation, **Poly–2** absorbs a broad spectral range and delivers notable hydrogen evolution activity (27.1 mmol g^−1^ in 9 h) with a quantum efficiency of 2.9% at 400 nm. Importantly, the energy band analysis (Figure 40a, right) shows that the conduction band potential of **Poly–2** lies above the H_2_/H_2_O redox level, providing sufficient driving force for proton reduction to H_2_. This band alignment confirms that **Poly–2** is thermodynamically eligible of catalyzing water splitting. This study underscores the promise of Tpy–Zn coordination polymers as efficient photocatalysts for solar–driven water splitting using earth–abundant metal centers.

Using the same principle, Maji and co–workers reported a supramolecular coordination polymer gel (**Gel–6**, Figure 41b) constructed from Zn(II) ions and an anthracene–based Tpy ligand [154]. In this system, the anthracene unit acts as an efficient visible–light harvester, while the [Tpy–Zn–Tpy]^2+^ node provides the catalytic center. The resulting 1D nanofibrous **Gel–6** exhibits robust soft–matter characteristics and enables visible–light–driven hydrogen evolution (400–750 nm), achieving a production rate of 12.02 mmol g^−1^ within 22 h. Under full–spectrum irradiation (290–750 nm), the H_2_ yield further increases to 18.03 mmol g^−1^. Moreover, the energy band analysis (Figure 41b, right) reveals that the conduction band of **Gel–6** lies above the H_2_/H_2_O redox potential, providing a thermodynamically favorable driving force for proton reduction and confirming the feasibility of water splitting. Compared with the more crystalline **Poly–2**, **Gel–6** benefits from soft supramolecular self–assembly, which ensures intimate spatial colocalization of chromophore and catalytic units and promotes efficient exciton migration and charge transfer in aqueous media. This study demonstrates a complementary strategy that integrates D–π–A molecular design with supramolecular gelation to develop recyclable, earth–abundant photocatalysts for solar–driven water splitting.

**FIGURE 42:**
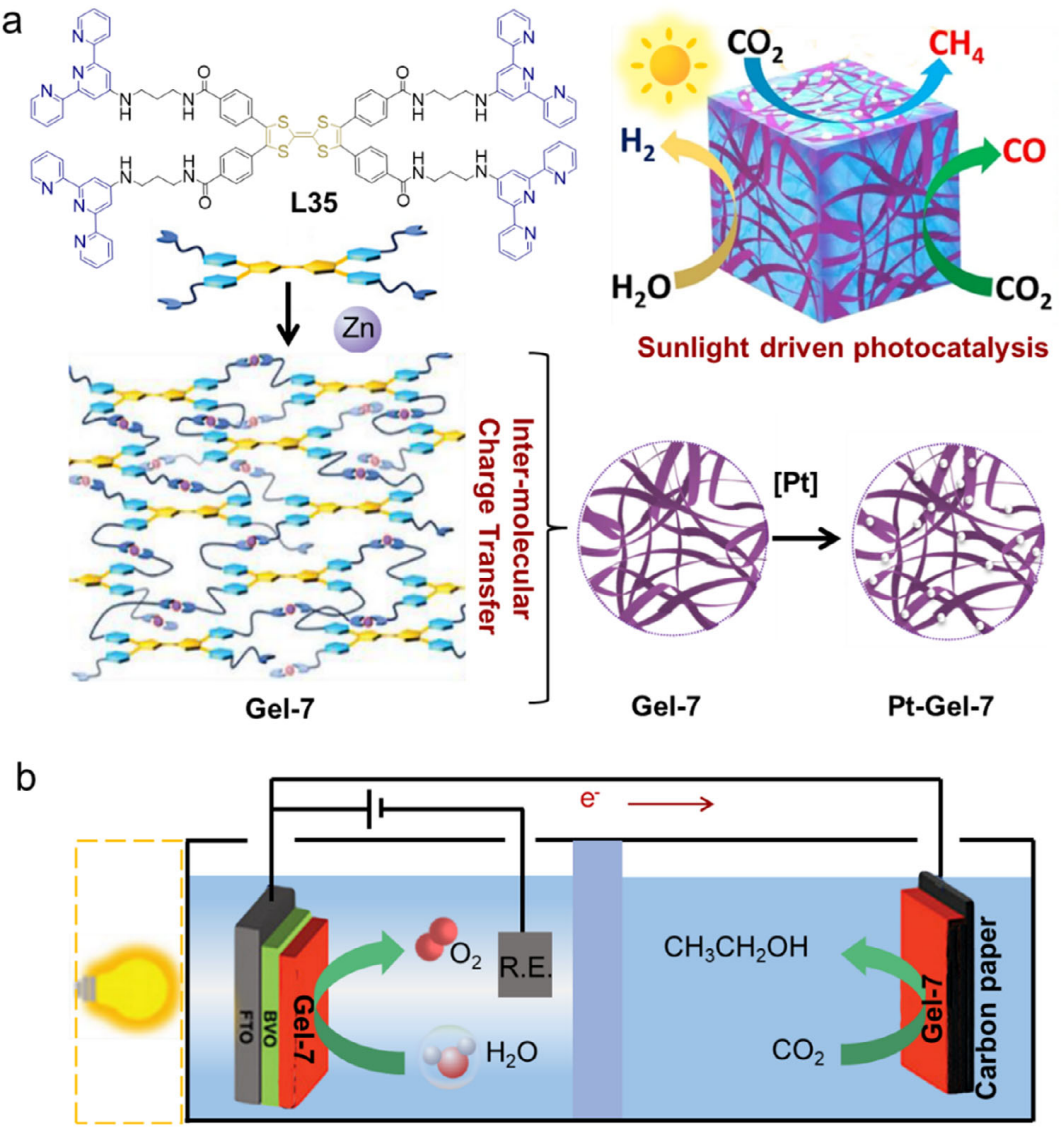
(a) Schematic illustration of the **Gel–7** formed by the self–assembly of a TTF based Tpy ligand **L35** and Zn(II) salts, and its visible–light–driven multifunctional catalytic performance for simultaneous H_2_ evolution and CO_2_ reduction to CO and CH_4_. Reproduced with permission [155]. Copyright 2021, Springer Nature. (b) PEC cell configuration using **Gel–7** as both photoanode and cathode material. The hybrid photoanode (**Gel–7**/BVO/FTO) facilitates water oxidation, while the cathode (**Gel–7**/carbon paper) enables CO_2_ reduction to CH_3_CH_2_OH. Reproduced with permission [156]. Copyright 2025, Wiley.

Subsequently, Maji et al. further developed a sulfur–rich tetrathiafulvalene (TTF)–Tpy based ligand **L35** that readily assembles with Zn(II) to form a soft coordination polymer gel (**Gel–7**, Figure 42a) [155]. Unlike previous D–π–A systems, the TTF core introduces strong electron–donating and redox–active properties, enhancing inter–molecular charge transfer interactions throughout the supramolecular matrix. The resulting **Gel–7** demonstrated dual photocatalytic functionality under visible light, achieving hydrogen evolution rates of 530 µmol g^−1^ h^−1^ and CO_2_–to–CO reduction selectivity >99%. Furthermore, in situ deposition of Pt nanoparticles onto the gel matrix yielded **Pt–Gel–7**, which significantly amplified hydrogen production (14,727 µmol g^−1^ h^−1^) and enabled selective CO_2_–to–CH_4_ conversion (292 µmol g^−1^ h^−1^, >97% selectivity). This outcome highlights the effectiveness of integrating redox–active sulfur motifs into soft coordination networks, offering an alternative design paradigm to traditional D–π–A frameworks with enhanced photoredox catalytic activity.

In a further development of **L28**–based systems, the **Gel–7** was readily integrated into a photoelectrochemical (PEC) device, expanding its functionality in photosynthesis (Figure 42b) [156]. Maji and co–workers fabricated a dual–functional PEC cell wherein **Gel–7** served simultaneously as the light–harvesting photoanode material and as the dark cathode catalyst. Specifically, the photoanode was constructed by forming a heterojunction between BiVO_4_ (BVO) nanostructures and the **Gel–7**, resulting in a significant enhancement of PEC water oxidation activity—a fourfold increase in photocurrent density (at 0.6 V vs. Ag/AgCl) compared with bare BVO. At the cathode side, **Gel–7** was immobilized on carbon paper and demonstrated excellent CO_2_–to–CH_3_CH_2_OH conversion with a Faradaic efficiency of 43% under bias–driven conditions. This integrated PEC system highlights the versatility of Tpy–Zn coordination gels not only in stand–alone photocatalysis but also as multifunctional components in solar–driven electrochemical platforms. This study indicates a strategic shift from simple molecular photocatalysts to soft–material–based PEC assemblies, offering new directions for sustainable solar fuel generation.

Recent advances underscore the versatile potential of Tpy–Zn systems in photocatalysis, encompassing reduction, oxidation, and even water splitting. Ligand engineering and coordination–driven assembly have proven effective in modulating electronic structures, promoting charge separation, and tailoring porous morphologies to enhance catalytic activity and selectivity. In CO_2_ reduction, shifting redox activity to the ligand framework has overcome the intrinsic limitation of the d^10^ configuration of Zn(II), while supramolecular assembly has enabled morphology–controlled reactivity. In the case of oxidation, embedding Tpy–Zn motifs into MOFs scaffolds creates confined microenvironments that facilitate substrate transformation under visible light. In water splitting, D–π–A structured polymers and redox–active soft gels have enabled hydrogen evolution without noble–metal co–catalysts and even integration into PEC systems. Despite these advances, challenges remain regarding long–term stability, quantum efficiency, and scalability. Continued innovations in molecular design and hybrid material integration are anticipated to extend the application of Tpy–Zn systems in solar–to–chemical energy conversion.

## 5 Conclusion and Outlook

Over the past decade, Tpy–Zn complexes have emerged as a cornerstone in the field of functional materials, benefiting from the modular ligand design, unique photophysical properties, and well–defined coordination geometry of the Zn(II) center. These complexes have proven to be a robust, highly tunable and functionally versatile, enabling the construction of diverse functional materials such as supramolecular gels, cages, coordination polymers, MOFs, and complicated composites. Their broad feasibility spans molecular recognition, stimuli– responsive and/or luminescent materials, as well as photocatalysis, showcasing their potential as a transformative platform in materials science.

In molecular recognition, Tpy–Zn complexes have been applied in phase–selective gel transformation, colorimetric and fluorescence modulation, providing a simple yet highly effective platform for rapid identification of biologically or environmentally relevant targets by naked eyes. In the realm of luminescent materials, the integration of AIE effects, photoisomerization, white–light tuning, CPL, and bioimaging functionalities has further extended their utility, paving a new way for high–performance sensors, anti–counterfeiting systems, and advanced theranostic technologies. In photocatalysis, rational ligand design and structural assembly have enabled precise control over excited–state dynamics, charge separation, and substrate interaction, facilating challenging reactions such as CO_2_ photoreduction, selective oxidation, and even water splitting.

Despite these remarkable advances, several challenges remain. The synthetic complexity and structural diversity of supramolecular assemblies, such as cages and frameworks, often hindered their scalability, reproducibility, and integration with other systems. The redox–inert nature of Zn(II) necessitates highly tailored ligand to enable efficient electron transfer, requiring further systematic studies to establish reliable structure–function relationships. Additionally, the relatively weak coordination bond between Tpy ligand and Zn(II) cetner may lead to reduced structural stability during catalytic process, especially under harsh conditions. Moreover, the limited solubility of some Tpy–Zn materials in water restricts their feasibility toward aqueous and biologically relevant scenarios, posing a significant barrier to their broader utilization.

To address these challenges and unlock the full potential of Tpy–Zn complexes, future research should focus on the following strategic directions:

1. Molecular simplification with enhanced functionality: By employing rational design strategies such as D–π–A architectures or supramolecular assembly approaches, simple molecular units can be endowed with enhanced optoelectronic and catalytic properties. This approach not only mitigates the synthetic and structural complexity but also retains diverse functionalities, making Tpy–Zn complexes more accessible for practical applications.
2. Biocompatibility and green synthesis: Designing Tpy ligands from bio–derived or degradable building blocks can promote environmental sustainability, aligning with the global shift toward green chemistry and reducing the ecological footprint of functional materials.
3. Smart device development: Incorporating Tpy–Zn molecules and corresponding materials into microfluidic sensors, solid–state lighting, or photoelectrochemical systems can bridge the gap between fundamental research and real–world applications, enabling the creation of next–generation intelligent devices.
4. Mechanistic understanding: Employing in situ spectroscopy and theoretical modeling to elucidate insights into coordination dynamics, excited–state behavior, and cooperative effects in assembled states will provide critical insights for optimizing material performance and guiding future design strategies.

In conclusion, Tpy–Zn complexes represent a powerful molecular toolbox that bridges fundamental coordination chemistry and advanced functional materials. With continued innovations in molecular design, supramolecular assembly, and fabrication strategies, this family is poised to play a pivotal role in the development of next–generation intelligent materials and technologies. Their versatility, tunability, and broad applicability make them a cornerstone for addressing some of the most pressing challenges in materials science, catalysis, and beyond, offering exciting opportunities for both academic research and industrial applications.

## Author Contributions

**Caiyun Fang**: validation, visualization. **Qingshu Zheng**: validation. **Yifan Fan**: funding acquisition, visualization. **Lixin Duan**: investigation, writing – original draft. **Tao Tu**: writing – review and editing, project administration, validation, investigation.

## Conflicts of Interest

The authors declare no conflict of interest.

## Data Availability

Data sharing is not applicable to this article, as no new datasets were generated or analyzed during the current study. The discussed in the manuscript were derived from previously published studies and publicly available sources, as cited in the manuscript.
